# Removal of the Micropollutants Propranolol Hydrochloride and 2-Naphthol From Water by Pyridine-Functionalized Polymers

**DOI:** 10.3389/fchem.2021.793870

**Published:** 2022-01-21

**Authors:** Qixuan Zheng, Daniel K. Unruh, Kristin M. Hutchins

**Affiliations:** Department of Chemistry and Biochemistry, Texas Tech University, Lubbock, TX, United States

**Keywords:** copolymer, micropollutants, pharmaceuticals, polymeric sorbents, supramolecular

## Abstract

The number and concentration of micropollutants in aqueous environments are increasing. Two such micropollutants include the pharmaceutical, propranolol hydrochloride, and dye intermediate, 2-naphthol. Here, we describe the synthesis of both linear and crosslinked pyridine-functionalized copolymers that bind and remove propranolol hydrochloride and 2-naphthol from water solutions. Propranolol hydrochloride and 2-naphthol both contain hydrogen-bond-donor groups, and the pyridine moiety on the polymer acts as a hydrogen-bond acceptor to facilitate removal. Copolymers with different amounts of pyridine comonomer are synthesized, and as the amount of the pyridine comonomer is increased, the ability of the polymer to bind and remove the contaminant also increases. The concentrations of propranolol hydrochloride and 2-naphthol decreased by approximately 20–40% and 60–88%, respectively, depending on the polymer type that is used in the binding experiment. A control polymer was synthesized by using styrene in place of the pyridine monomer. In analogous binding experiments, the styrene polymer decreases the concentration of propranolol hydrochloride by 2% and 2-naphthol by 26%. Thus, the binding effectiveness is significantly reduced when the hydrogen-bond-acceptor group is not present on the polymer. We also show that the best performing crosslinked pyridine-functionalized polymer is reusable. Overall, these polymer adsorbents demonstrate the potential for removal of micropollutants from water.

## Introduction

The fast-growing global consumption of products such as pharmaceuticals, pesticides, and personal-care products has led to the presence of organic micropollutants in the environment, especially in aquatic systems (Schwarzenbach et al., 2006). Although the effects of such micropollutants on human, animal, and plant health remain mostly unknown, there are concerns about negative impacts on aquatic life and human health (Alsbaiee et al., 2016; Weber et al., 2016). Most wastewater treatment plants are not equipped to remove micropollutants that enter the water supply (Bui et al., 2016). Activated carbon absorption has attracted significant attention for its effectiveness; however, the material shows poor affinity for polar compounds (Kovalova et al., 2013; Bui et al., 2016). Advanced oxidation processes have also shown promise in reducing micropollutants, but some oxidation products remain toxic (Hollender et al., 2009; Zimmermann et al., 2011; Prasse et al., 2012). Methods and/or materials that target and remove contaminants without generating byproducts (Xiao et al., 2019) will aid in addressing contamination problems. Recently, adsorbent materials including macrocycles (Ji et al., 2020; Klemes et al., 2020; Dai et al., 2021), covalent organic frameworks (Skorjanc et al., 2021), assembled molecular rings (Liu et al., 2021), and crosslinked polymers (Wang et al., 2021; Yang L. P et al., 2021) have been designed and synthesized for removal of micropollutants from water.

**FIGURE 1:**
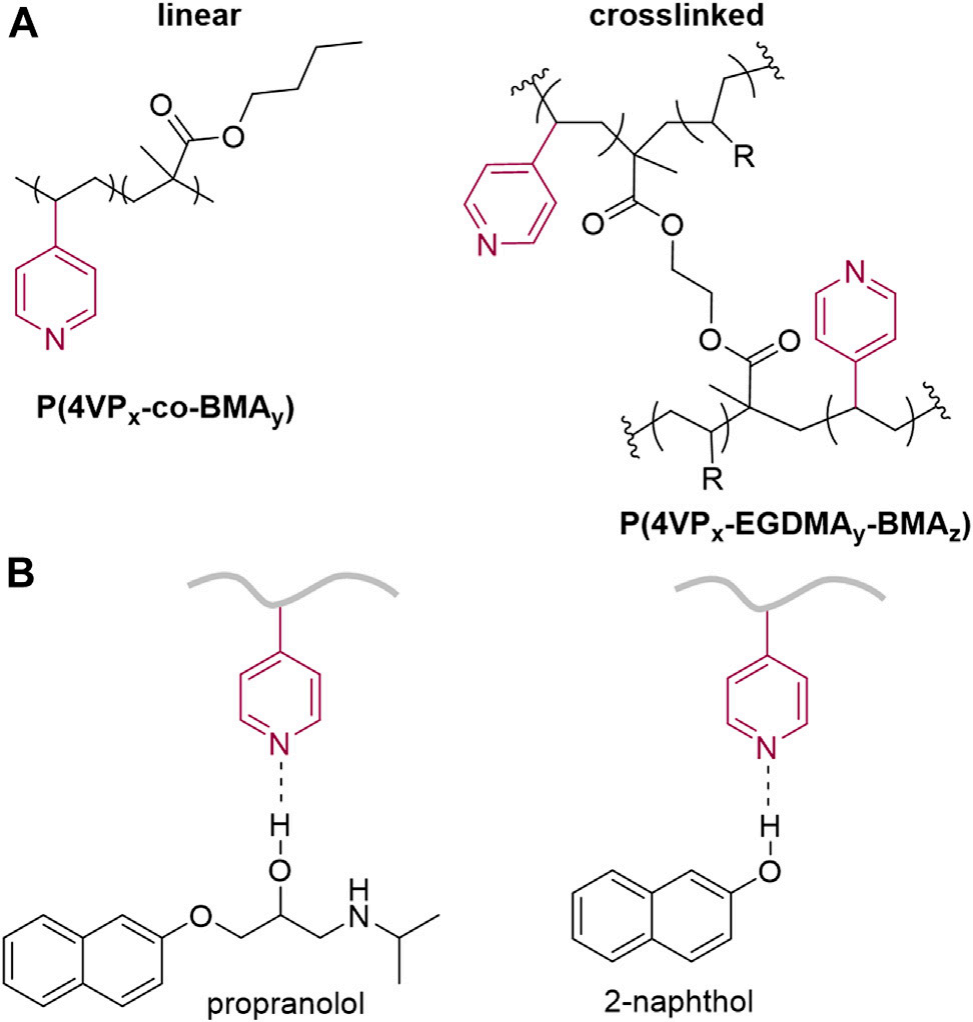
Chemical structures of **(A)** linear and crosslinked pyridine-functionalized polymers and **(B)** hydrogen-bonding interactions between contaminants and polymer.

Propranolol hydrochloride (**PPL-HCl**) is a beta blocker that has been found in aquatic environments, and typical wastewater treatment plant removal is less than 30% (Margot et al., 2015). 2-Naphthol (**2NO**) is an intermediate in the production of dyes and other compounds (e.g., BINOL) and has also been found as a water contaminant (Wang et al., 2006; Zang and Lian, 2009). Both **PPL-HCl** and **2NO** feature aromatic and hydroxyl groups. Polymers and porous materials have been designed and synthesized to remove these two micropollutants from water (Table 1). In 2016, Dichtel et al. reported a porous *β*-cyclodextrin polymer that can remove 96% of **PPL-HCl** or 91% **2NO** from aqueous solution *via* flow-through adsorption in 30 min (Alsbaiee et al., 2016). In 2018, Trabolsi et al. reported porous polycalix [4]arenes that can remove around 85% of **PPL-HCl** or 75% **2NO** from aqueous solutions (Shetty et al., 2018). In 2021, Song et al. reported a *β*-cyclodextrin-polyacrylamide hydrogel that can remove 54% of **PPL-HCl** or 60% of **2NO** from aqueous solution through host-guest interactions in a period of 8 h (Song et al., 2021). We postulated that polymers functionalized with aromatic groups and hydrogen-bond-acceptor moieties (Díez-Pascual et al., 2016) could form noncovalent interactions with the contaminants to facilitate adsorption. Here, we demonstrate both linear and crosslinked pyridine-functionalized polymers can bind and remove **PPL-HCl** and **2NO** from water solutions. Both the linear and crosslinked polymers contain *n*-butylmethacrylate (**BMA**) and 4-vinylpyridine (**4-VP**) as comonomers, and the crosslinked polymers also include ethylene glycol dimethacrylate (**EGDMA**) as the crosslinker (Figure 1A). The **4-VP**:**BMA** comonomer ratio was systematically increased to afford polymers with different binding abilities. Overall, polymers with higher **4-VP**:**BMA** ratios exhibit greater binding and removal ability. The concentrations of **PPL-HCl** and **2NO** in aqueous solutions decreased by approximately 20–40% and 60–88%, respectively, depending on the type of polymer used. All the polymers contain pyridine rings and carbonyl functional groups, which are both capable of acting as hydrogen-bond acceptor sites (Figure 1B). Small molecule cocrystallization experiments with **2NO** and methyl isonicotinate (**MI**), which contains pyridyl and ester functional groups and mimics the polymer functional groups, demonstrate that hydrogen bonding likely supports binding. Moreover, a control polymer synthesized using styrene in place of the pyridine monomer exhibited significantly reduced binding ability.

**TABLE 1:** The removal performance of reported materials and polymers in this work for **PPL-HCl** and **2NO**.

Material	Concentration of PPL-HCl water solution (mM)	Concentration decrease for PPL-HCl water solution (%)	Concentration of 2NO water solution (mM)	Concentration decrease for 2NO water solution (%)	Reference
porous *β*-cyclodextrin polymer	0.09	96	0.1	91	Alsbaiee et al. (2016)
β-cyclodextrin-polyacrylamide hydrogel	0.035–1.0	54	0.035–1.0	60	Song et al. (2021)
porous polycalix [4]arenes	0.1	85	0.1	75	Shetty et al. (2018)
biomass alginate	1.0	50–90	—	—	Coelho et al. (2020)
carbon nanotube-based composite adsorbent	—	—	0.035	60–70	Xu et al. (2018)
pyridine-functionalized copolymers	0.4	40	0.1	88	this work

## Materials and Methods

### Materials

Methyl isonicotinate (**MI**), and propranolol hydrochloride (**PPL-HCl**) were purchased from Oakwood Chemical. 4-vinylpyridine (**4-VP**) and ethylene glycol dimethlacrylate (**EGDMA**) were purchased from Alfa Aesar. *n*-Butylmethacrylate (**BMA**) and styrene (**STY**) were purchased from ACROS Organics. Benzoyl peroxide (**BPO**) and azobisisobutyronitrile (**AIBN**) were purchased from Sigma-Aldrich Chemical. 2-Naphthol (**2NO**) was purchased from Tokyo Chemical Industry Co., LTD. Diethyl ether, methanol, toluene, tetrahydrofuran (THF), and basic alumina were purchased from Fisher Scientific. Inhibitors in commercial **4-VP**, **EGDMA**, **BMA**, and **STY** were removed by passage through basic alumina. All other chemicals and solvents were used as received. Full details for all experiments are available in the supporting information.

### General Synthesis of Linear Polymers: Poly(4-Vinyl Pyridine-co-butylmethacrylate) [P(4VP_x_-co-BMA_y_)]

The monomers **4-VP** (variable, see Table 2) and **BMA** (4.2660 g, 30 mmol) were added to a 100 ml round bottom flask containing THF (30 ml) followed by addition of **AIBN** (0.1641 g, 0.9 mmol) to initiate polymerization. The mixture was heated to 60°C in an oil bath under a nitrogen atmosphere. Polymerization was conducted for 18 h or 36 h. The reaction was cooled to room temperature and approximately two-thirds of the THF was removed using a rotavap. Diethyl ether was added to precipitate the copolymer. The product was then dissolved in methanol and the undissolved compounds were removed by filtration (homopolymer **P-BMA** is insoluble in methanol). The polymer was re-precipitated with diethyl ether. The precipitate was dissolved in toluene and the undissolved compounds were removed by filtration (homopolymer **P-4VP** is insoluble in toluene). The polymer was re-precipitated with diethyl ether and isolated. The last two steps (dissolving in methanol and toluene anti-solvents and precipitating) were repeated twice to ensure all unreacted monomers, homopolymers and initiator were removed. The copolymer was dried under vacuum overnight then dried under vacuum with heating at 125°C overnight.

**TABLE 2:** Linear polymers: theoretical/synthetic feed ratios of monomers used, obtained ratios, and characterization data.

Linear polymer	Theoretical and synthetic feed ratio 4VP:BMA	Reaction time (h)	Obtained ratio^a^	M_n_ (kDa)	M_w_ (kDa)	*Đ*
P(4VP_1_-co-BMA_1_)	1:1 (30:30 mmol)	18	1:0.82	1.2	5.8	4.6
P(4VP_2_-co-BMA_1_)	2:1 (60:30 mmol)	18	2:0.77	13.3	23.4	1.8
P(4VP_3_-co-BMA_1_)	3:1 (90:30 mmol)	18	3:0.84	11.1	18.1	1.6
P(4VP_3_-co-BMA_1_)_t_	3:1 (90:30 mmol)	36	3:0.88	17.0	28.7	1.7

a Based on ^1^H NMR, spectroscopy.

### General Synthesis of Crosslinked Polymers: Poly(4-Vinyl Pyridine-Ethylene Glycol Dimethlacrylate-Butylmethacrylate) [P(4VP_x_-EGDMA_y_-BMA_z_)]

Deionized water (110 ml), Mowiol 40–88 (PVA, 0.25 g), **BMA** (22.041 g, 155 mmol), **4-VP** (variable, see Table 3), **EGDMA** (variable, see Table 3), and **BPO** (0.50 g, 2.1 mmol) were added to a 300 ml three-neck flask. The mixture was stirred at 240 rpm using an IKA 20 digital mechanical stirrer, purged with nitrogen for 15 min, and heated to 70°C for 12 h. The polymer particles were isolated *via* centrifugation at 3,000 rpm for 3 min. The particles were washed five times using THF (30 ml) and EtOH (70 ml) and centrifuged at 3,000 rpm for 3 min and dried under vacuum to give white solid beads. The polymer beads were purified by Soxhlet extraction using THF at 110°C overnight and subsequently dried under vacuum heating at 120°C overnight. To confirm the crosslinked polymers were dry and free of trapped solvent or synthetic byproducts, a portion of polymer beads was stirred and heated at 95°C in water for 2 h. The solution was filtered to remove the polymer beads and characterized by UV-Vis spectroscopy, which demonstrated minimal signal (Supplementary Figure S18).

**TABLE 3:** Synthetic feed of monomers used to synthesize crosslinked polymers.

Crosslinked polymer	4-VP (mmol)	EGDMA (mmol)	BMA (mmol)
P(4VP_1_-EGDMA_0.5_-BMA)	9.8	0.75	155
P(4VP_1_-EGDMA_1.0_-BMA)	9.8	1.5	155
P(4VP_2_-EGDMA_1.0_-BMA)	19.6	1.5	155
P(4VP_3_-EGDMA_1.0_-BMA)	29.4	1.5	155
P(4VP_1_-EGDMA_2.0_-BMA)	9.8	3.0	155

### Synthesis of a Control Crosslinked Polymer: Poly(Styrene_3_-Ethylene Glycol Dimethlacrylate_1.0_-Butylmethacrylate)

Deionized water (110 ml), Mowiol 40–88 (PVA, 0.25 g), **BMA** (22.041 g, 155 mmol), **STY** (3.06 g, 29.4 mmol), **EGDMA** (0.2973 g, 1.5 mmol), and **BPO** (0.50 g, 2.1 mmol) were added to a 300 ml three-neck flask. The mixture was stirred at 240 rpm using an IKA 20 digital mechanical stirrer, purged with nitrogen for 15 min, and heated to 70°C for 12 h. The polymer particles were isolated *via* centrifugation at 3,000 rpm for 3 min. The particles were washed five times using THF (30 ml) and EtOH (70 ml) and centrifuged at 3,000 rpm for 3 min and dried under vacuum to give white solid beads. The polymer beads were purified by Soxhlet extraction using THF at 110°C overnight and subsequently dried under vacuum heating at 120°C overnight.

### Solution Binding Experiments

Binding experiments for the linear polymers were conducting by adding 10 mg of the polymer to a vial. Then, 2.5 ml of a **PPL-HCl** solution in water (0.4 mmol L^−1^) or a **2NO** solution in water (0.1 mmol L^−1^) was added to the same vial. Seven individual samples were prepared, and each mixture was gently stirred for a different period of time at room temperature. The time periods were 15, 30, 45 min, 1, 2, 3, or 4 h. The resulting solution in each vial was filtered through cotton to remove the polymer. The filtrates were diluted with water using a micropipette and volumetric flask, and the concentrations after dilution were measured by UV-Vis (Supplementary Figures S19–S34). Aqueous solutions of **PPL-HCl** and **2NO** were colorless before and after binding experiments.

Binding experiments for the crosslinked polymers were conducting by adding 200 mg of polymer beads to a vial. Then, 2.5 ml of a **PPL-HCl** solution in water (0.4 mmol L^−1^) or a **2NO** solution in water (0.1 mmol L^−1^) was added to the same vial. Eight individual samples were prepared, and each mixture was gently stirred for a different period of time at room temperature. The time periods were 15, 30, 45 min, 1, 2, 3, or 4 h, or overnight (17 h). The resulting solution in each vial was filtered through cotton to remove the polymer. The filtrates were diluted with water using a micropipette and volumetric flask, and the concentrations after dilution were measured by UV-Vis (Supplementary Figures S35–S56). HPLC also confirmed that the decrease in the signal observed by UV-Vis correlates to a decrease in the signal at retention time corresponding to **2NO** (Supplementary Table S5).

Binding experiments for the control polymer **P(STY**_**3**_ **-EGDMA**_**1.0**_ **-BMA)** were conducting by adding 200 mg of polymer beads to a vial. Then, 2.5 ml of a **PPL-HCl** solution in water (0.4 mmol L^−1^) or a **2NO** solution in water (0.1 mmol L^−1^) was added to the same vial. Each mixture was gently stirred at room temperature. The stirring time was 2 h for the **PPL-HCl** solution and 4 h for the **2NO** solution, which correlate to the best binding times for the best performing crosslinked pyridine polymer **(P**(**4VP**_**3**_ **-EGDMA**_**1.0**_ **-BMA)**. The resulting solution in each vial was filtered through cotton to remove the polymer. The filtrates were diluted with water using a micropipette and volumetric flask, and the concentrations after dilution were measured by UV-Vis (Supplementary Figures S57–S58).

## Results and Discussion

### Synthesis and Characterization of Pyridine-Functionalized Polymers

**FIGURE 2:**
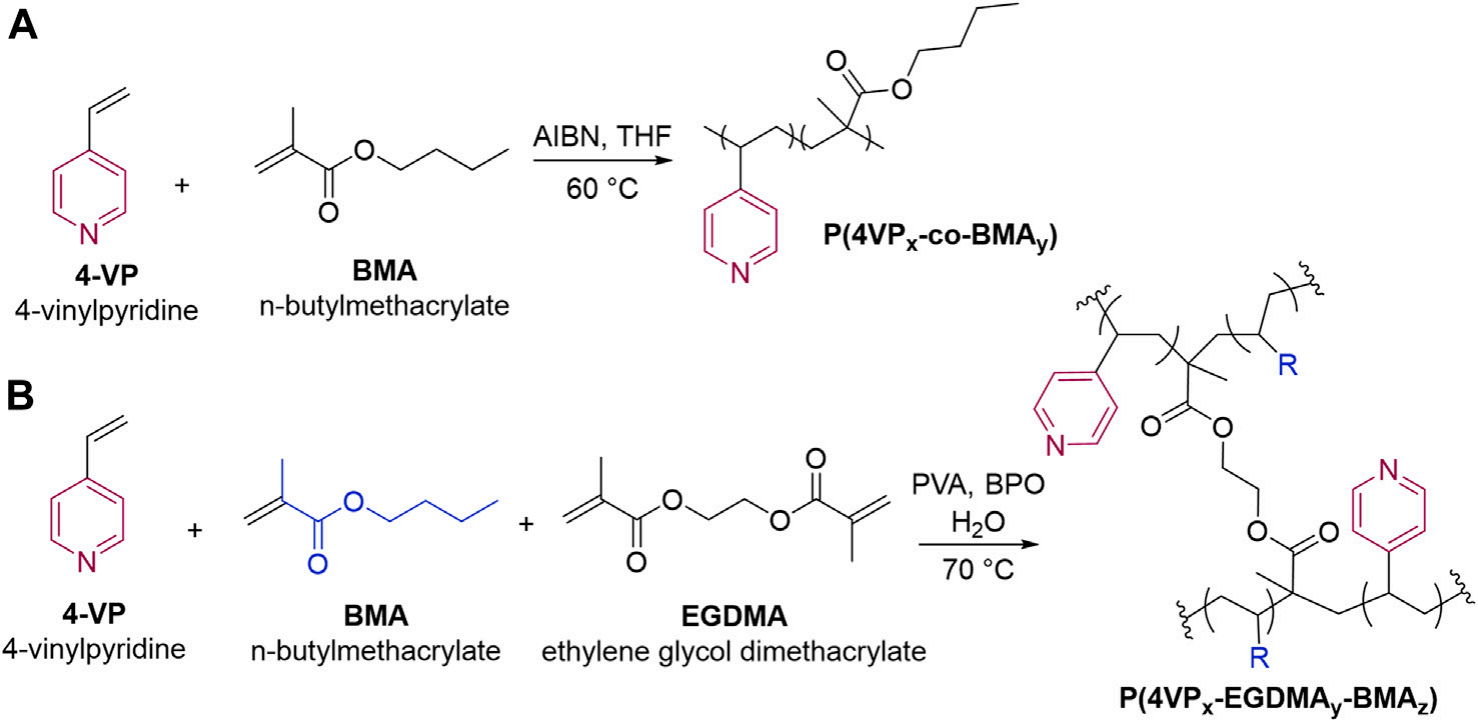
Synthetic outline for **(A)** linear and **(B)** crosslinked pyridine-functionalized polymers.

We set out to synthesize pyridine-functionalized polymers that could act as adsorbents for micropollutants containing hydroxyl groups. Linear, pyridine-functionalized polymers **P(4VP**_**x**_ **-co-BMA**_**y**_) were synthesized by free-radical solution polymerization, and crosslinked pyridine-functionalized polymers **P(4VP**_**x**_ **-EGDMA**_**y**_ **-BMA**_**z**_) were synthesized using suspension polymerization (Figure 2). **BMA** was chosen as a comonomer due to its ability to impart hydrophilicity onto the polymer (Ouellette and Rawn, 2014). Copolymers of **4VP** and **BMA** have been synthesized previously (Goswami and Dutta, 2013); however, to our knowledge, they have not been utilized as an adsorbent for removing pollutants.

The number of pyridine functional groups on the polymer backbone could have an impact on the binding ability of the polymers. Therefore, the **4-VP**:**BMA** comonomer ratio was systematically increased to yield a series of polymers. Four linear polymers were synthesized, namely, **P(4VP**_**1**_ **-co-BMA**_**1**_ **)**, **P(4VP**_**2**_ **-co-BMA**_**1**_ **)**, **P(4VP**_**3**_ **-co-BMA**_**1**_ **)**, and **P(4VP**_**3**_ **-co-BMA**_**1**_)_**t**_ (where subscript t denotes a doubled reaction time, see Table 2). The additional reaction time was used to increase the molecular weight (Table 2) and determine if there was any impact on binding ability. Five crosslinked polymers were synthesized, namely, **P(4VP**_**1**_ **-EGDMA**_**0.5**_ **-BMA)**, **P(4VP**_**1**_ **-EGDMA**_**1.0**_ **-BMA)**, **P(4VP**_**2**_ **-EGDMA**_**1.0**_ **-BMA)**, **P(4VP**_**3**_ **-EGDMA**_**1.0**_ **-BMA)**, and **P(4VP**_**1**_ **-EGDMA**_**2.0**_ **-BMA)**. The amount of **BMA** added to the reaction was held constant, while the **4-VP** and **EGDMA** crosslinker amounts were altered systematically (Table 3).

**FIGURE 3:**
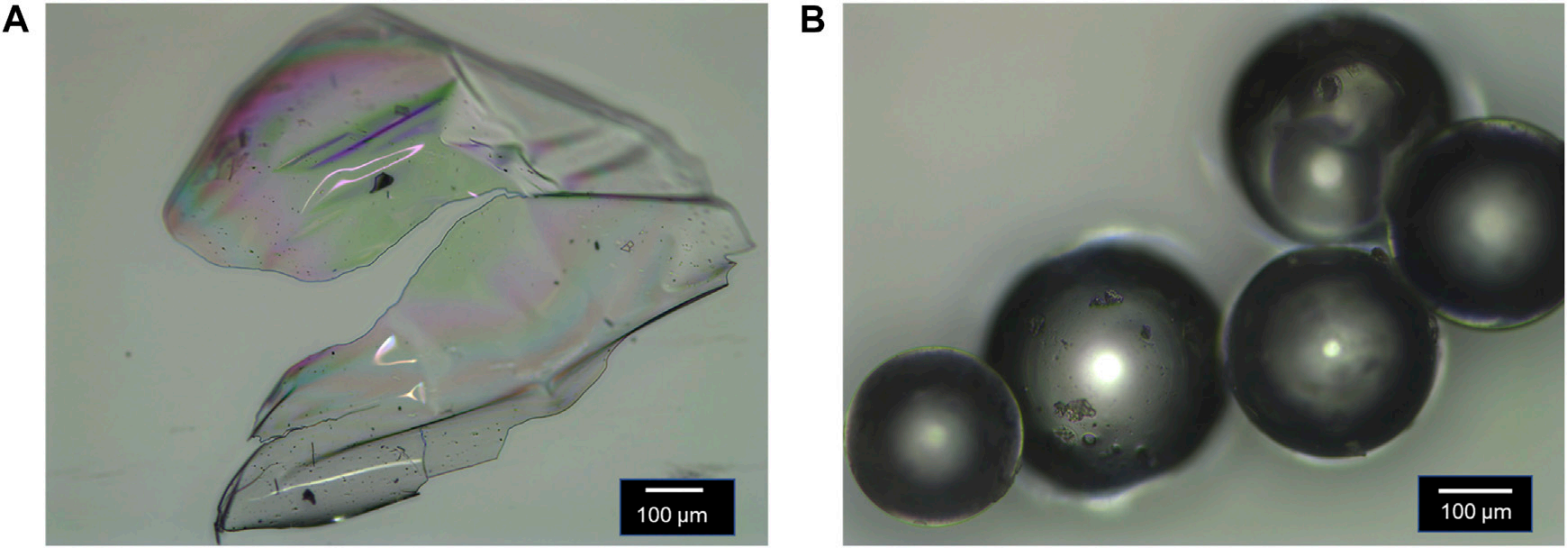
Morphology of: **(A)** linear polymer **P(4VP**_**1**_ **-co-BMA**_**1**_ **)** and **(B)** crosslinked polymer **P(4VP**_**1**_ **-EGDMA**_**1.0**_ **-BMA)**. Both images were collected after the polymer was dried under vacuum with heat.

The linear polymers exhibit a semitransparent, glassy morphology, and the crosslinked polymers are polydisperse and exhibit a spherical morphology as evidenced by optical microscopy (Figure 3). ^1^H NMR spectroscopy confirmed the obtained monomer ratios in the polymer are similar to synthetic feed of monomers used (Supplementary Figures S4–S7, Table 2). Gel permeation chromatography (GPC) confirmed high molecular weight for the linear polymers (Supplementary Figures S14–S17, Table 2). ^13^C NMR spectroscopy confirmed the chemical functionality of the crosslinked polymers (Supplementary Figures S8–S12).

### Binding Ability of the Linear Polymers

**FIGURE 4:**
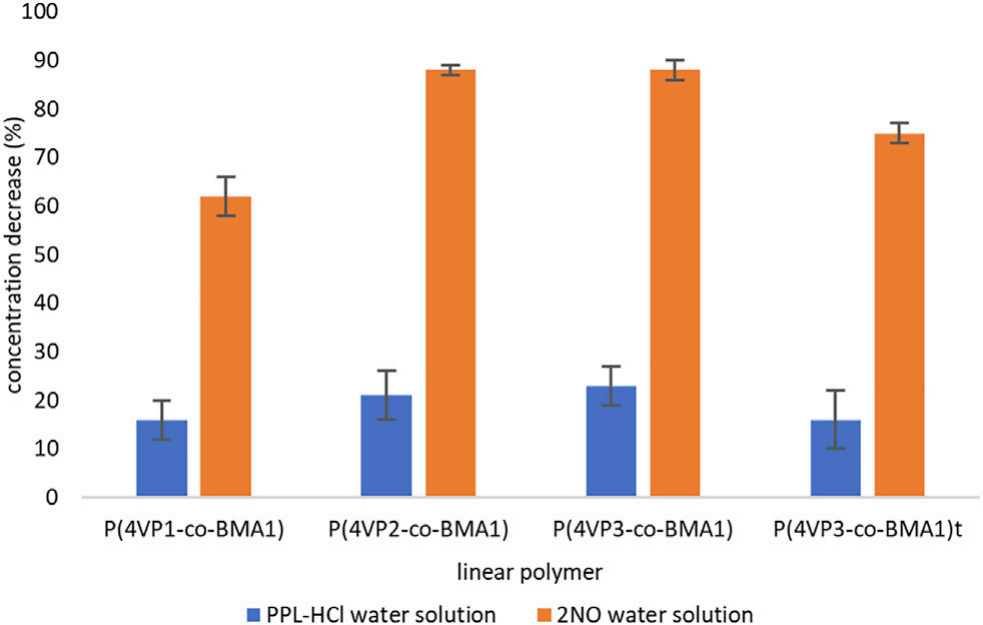
Decreases in concentrations of contaminants following binding experiments for linear polymers.

To determine the capability of the pyridine-functionalized polymers for binding **PPL-HCl** or **2NO** from water, binding experiments were conducted, and solutions after binding were characterized by UV-Vis spectroscopy. Binding experiments were conducted for periods of 15, 30, 45 min, 1, 2, 3, or 4 h at room temperature. All four synthesized linear polymers successfully bound **PPL-HCl** and **2NO** from solutions of water. Generally, binding experiments conducted for a period of approximately 4 h afforded the highest removals of **PPL-HCl** and **2NO** (Supplementary Table S2, Figure 4). However, a decrease in concentration was observed in as little as 15 min for each sample (Supplementary Figures S19–S34). After determining the optimal binding time (i.e. the time at which the concentrations of **PPL-HCl** or **2NO** water solution decreased most), three additional trials with the same binding time were conducted to calculate standard deviations (Figure 4).

**FIGURE 5:**
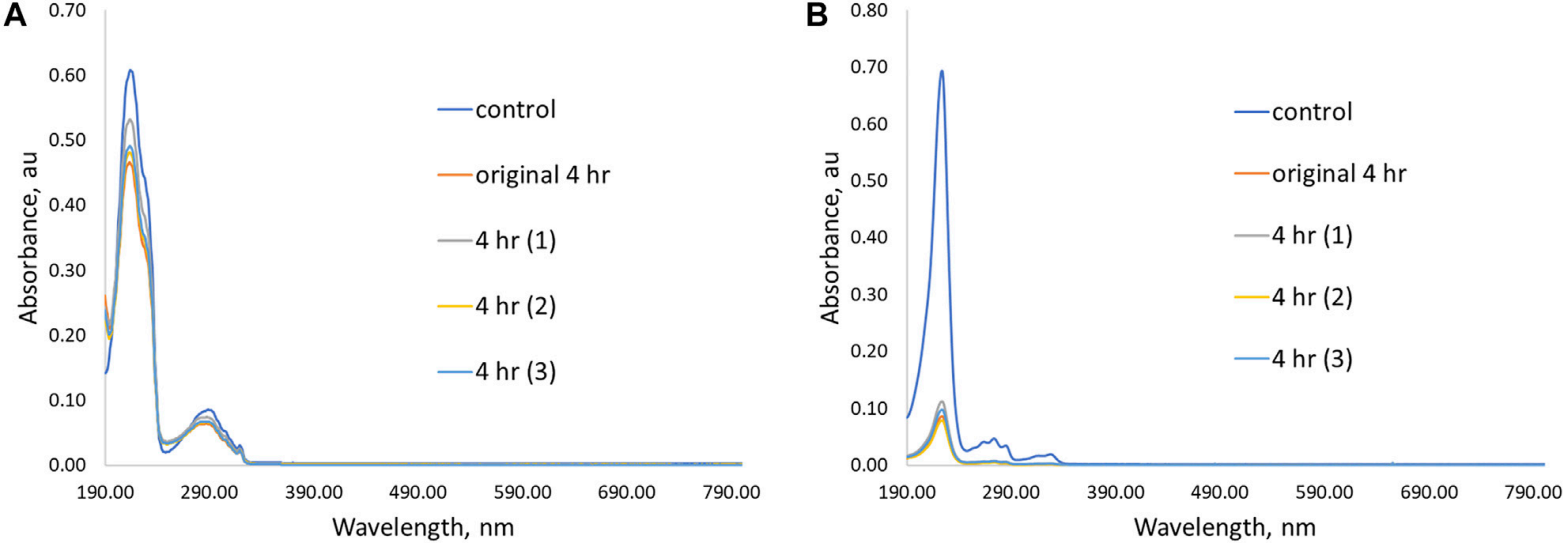
UV-Vis spectra showing initial removal ability and three additional trial experiments for **P(4VP**_**3**_ **-co-BMA**_**1**_ **)** in aqueous solutions of **(A) PPL-HCl** for 4 h and **(B) 2NO** for 4 h.

For the **PPL-HCl** experiments, all four linear polymers removed ca. 20% of the drug at their optimal binding time. On the other hand, higher amounts of **4-VP** in the linear polymers resulted in significantly better binding ability for the **2NO** contaminant. The best performing linear polymer overall was **P(4VP**_**3**_ **-co-BMA**_**1**_ **)**, which decreased the concentration of **PPL-HCl** in water by 23% and decreased the concentration of **2NO** in water by 88% (Figure 5). The binding performance of these polymers with **PPL-HCl** is lower than reported *β*-cyclodextrin materials; however binding performance with **2NO** is on par with the reported *β*-cyclodextrin materials (Table 1, Alsbaiee et al., 2016; Song et al., 2021). The polymer **P(4VP**_**2**_ **-co-BMA**_**1**_ **)** also bound **2NO** just as effectively as **P(4VP**_**3**_ **-co-BMA**_**1**_ **)**. The higher molecular weight polymer **P(4VP**_**3**_ **-co-BMA**_**1**_)_**t**_ did not demonstrate enhanced ability to bind either contaminant. The standard deviations for the **PPL-HCl** binding experiments are higher than for the **2NO** experiments. As removal efficiency for **PPL-HCl** is lower than **2NO**, this indicates that binding between the two species is likely not as strong.

### Binding Ability of the Crosslinked Polymers

Akin to linear polymers, binding experiments with the same **PPL-HCl** or **2NO** solutions in water were conducted using the crosslinked polymers as adsorbents. The same binding experiment times were used as the linear polymers. The UV-Vis spectra demonstrated that all five crosslinked polymers also successfully removed **PPL-HCl** and **2NO** from solutions of water. Generally, a binding period of 1–2 h for **PPL-HCl** or 4 h for **2NO** resulted in the highest removal percentages (Supplementary Table S3, Figure 6). A decrease in concentration was observed in as little as 15 min for each sample as well (Supplementary Figures S35–S45, S46–S56). After determining the time at which concentrations of **PPL-HCl** or **2NO** decreased most, three additional trials with the same binding time were conducted to calculate standard deviations (Figure 6).

**FIGURE 6:**
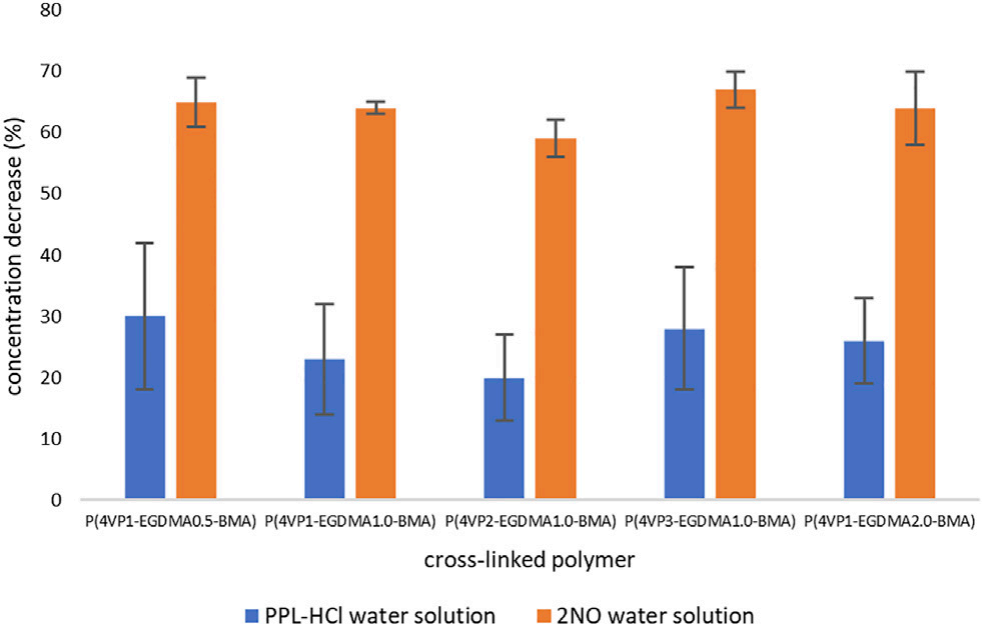
Decreases in concentrations of contaminants following binding experiments for crosslinked polymers.

**FIGURE 7:**
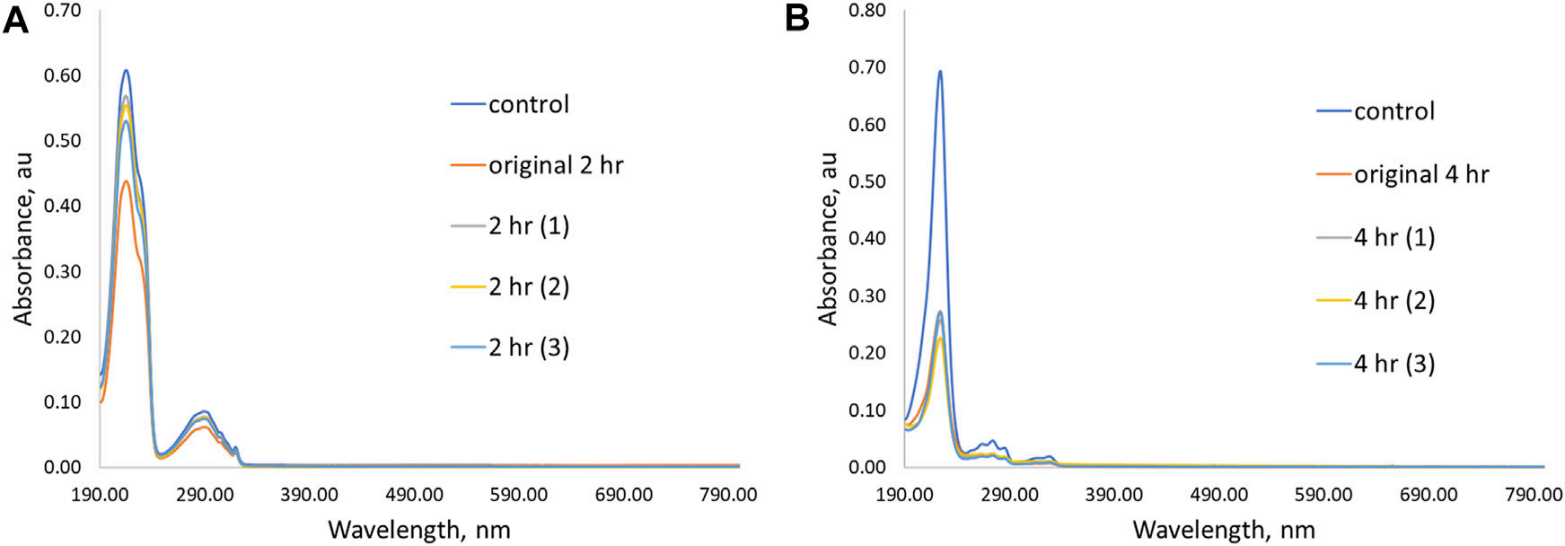
UV-Vis spectra showing initial removal ability and three additional trial experiments for **P(4VP**_**3**_ **-EGDMA**_**1.0**_ **-BMA)** in aqueous solutions of **(A) PPL-HCl** for 2 h and **(B) 2NO** for 4 h.

For the **PPL-HCl** experiments, all five crosslinked polymers removed ca. 20–30% of the drug at their optimal binding time. On the other hand, removal of **2NO** was significantly higher at about 65%. The amount of **EGDMA** crosslinker and the amount of **4-VP** in the polymer play an important role in binding ability. When the amount of **EGDMA** is held constant at 1 mol% and the amount of **4-VP** is systematically increased to afford the three polymers **P(4VP**_**1**_ **-EGDMA**_**1.0**_ **-BMA)**, **P(4VP**_**2**_ **-EGDMA**_**1.0**_ **-BMA)**, and **P(4VP**_**3**_ **-EGDMA**_**1.0**_ **-BMA)**, the binding ability is highest for the polymer with the largest amount of pyridine functionalization, namely, **P(4VP**_**3**_ **-EGDMA**_**1.0**_ **-BMA)** (Figure 7). Interestingly, **P(4VP**_**2**_ **-EGDMA**_**1.0**_ **-BMA)** does not exhibit increased binding ability for **PPL-HCl** or **2NO** compared to **P(4VP**_**1**_ **-EGDMA**_**1.0**_ **-BMA)**, as was the case for the linear polymers.

When the amount of **EGDMA** is increased from 0.5 to 1 and to 2 mol% while holding **4-VP** constant to afford the three polymers **P(4VP**_**1**_ **-EGDMA**_**0.5**_ **-BMA)**, **P(4VP**_**1**_ **-EGDMA**_**1.0**_ **-BMA)**, and **P(4VP**_**1**_ **-EGDMA**_**2.0**_ **-BMA)**, the average binding ability decreases for **PPL-HCl**, but remains similar for **2NO**. The lower amount of crosslinker allows the polymer bead to swell more readily in solution (Pulko and Krajnc, 2005; Nigmatullin et al., 2014), which results in increased binding ability. Particle swelling has also been demonstrated to influence the effectiveness of solid-phase synthesis, whereby swelling allows nearly all reactive sites within the particle to be chemically accessible (Engström and Helgee, 2006; Nakaie et al., 2011; Mane et al., 2014). As observed for the linear polymers, the standard deviations for the **PPL-HCl** binding experiments with crosslinked polymers are also higher than for the **2NO** experiments, indicating lower binding effectiveness.

**FIGURE 8:**
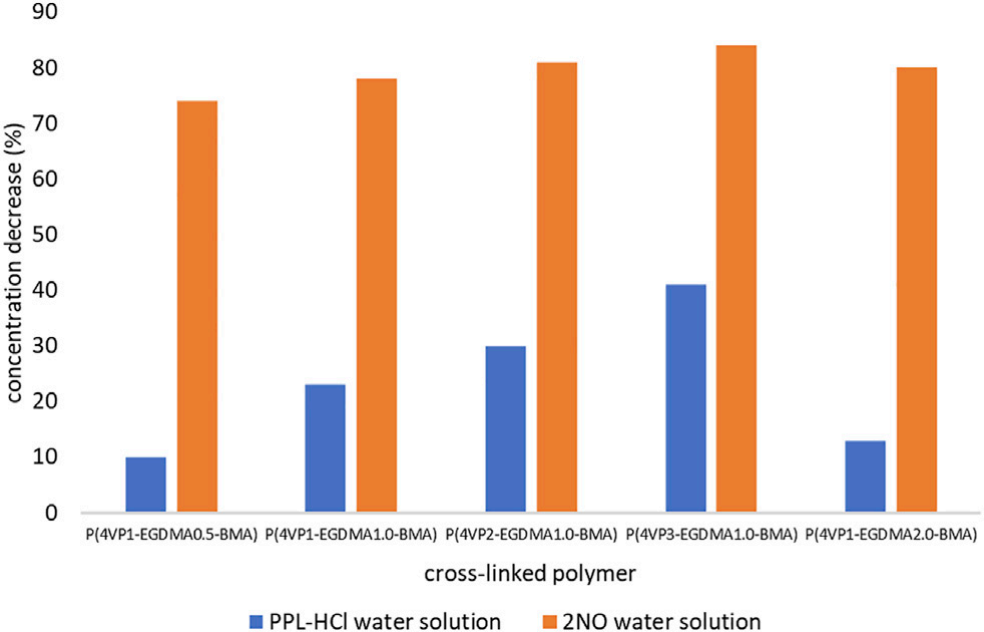
Decreases in concentrations of contaminants following overnight binding experiments (17 h) for crosslinked polymers.

**FIGURE 9:**
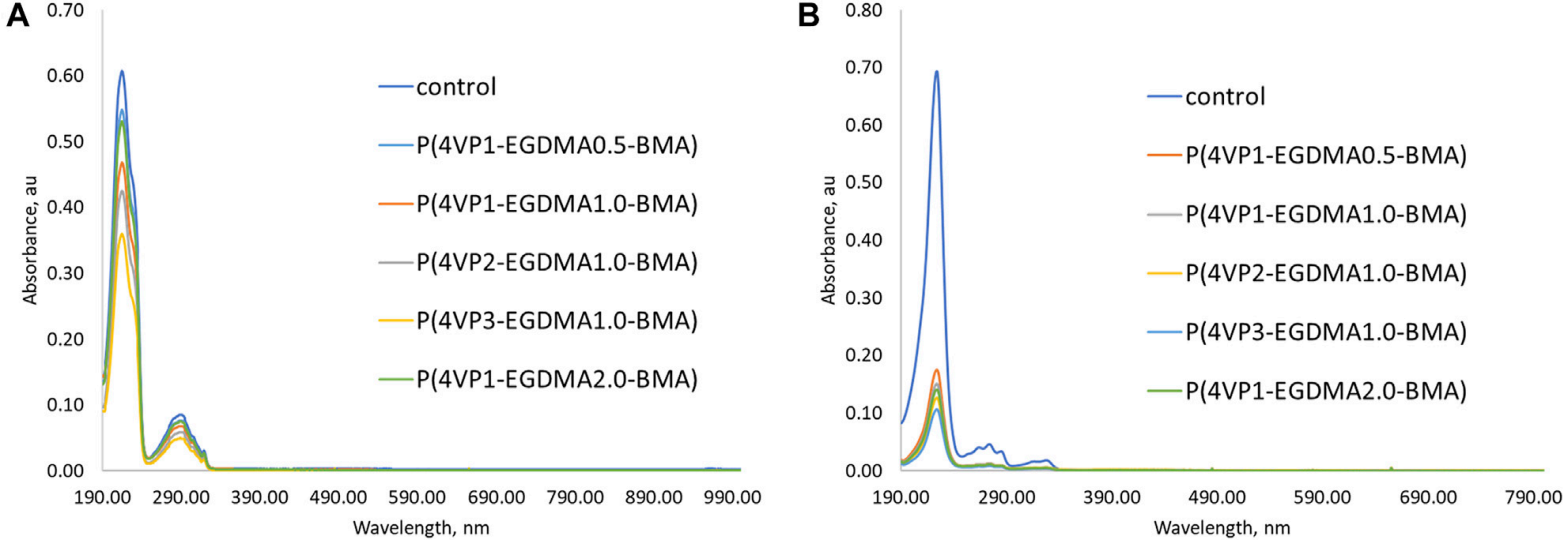
UV-Vis spectra for the five crosslinked polymer beads following overnight (17 h) binding experiment in aqueous solutions of **(A) PPL-HCl** and **(B) 2NO**.

In addition to the time experiments ranging from 15 min to 4 h, we also conducted an overnight (17 h) binding experiment for the crosslinked polymer beads (Figure 8, Supplementary Table S4). The longer time allows the beads to swell in solution and could result in increased removal abilities. Only two polymers exhibited enhanced binding of **PPL-HCl** with increased binding time, namely **P(4VP**_**2**_ **-EGDMA**_**1.0**_ **-BMA)** and **P(4VP**_**3**_ **-EGDMA**_**1.0**_ **-BMA)**. On the other hand, binding of **2NO** increased to ca. 80% with increased binding time for all the crosslinked polymers. Overall, the best performing polymer was **P(4VP**_**3**_ **-EGDMA**_**1.0**_ **-BMA)**, which decreased the concentration of **PPL-HCl** in water by 41% and decreased the concentration of **2NO** in water by 84% (Figure 9).

### Reusability of the Highest Performing Crosslinked Polymer

Reusability is an essential performance indicator of a given adsorbent (Yang W. et al., 2021). Thus, after determining binding ability of the synthesized polymers, we were interested in evaluating the reusability. The crosslinked polymers are not soluble in organic solvents, whereas the linear polymers are. Thus, we chose to examine reusability for the best performing crosslinked polymer, **P(4VP**_**3**_ **-EGDMA**_**1.0**_ **-BMA)** with **2NO**. After the first 4 h binding experiment, the beads were purified by a Soxhlet extraction using THF at 110°C overnight and subsequently dried under vacuum heating at 120°C overnight. A second 4 h binding experiment was conducted, and the polymer beads remove **2NO** at the same level, demonstrating good reusability (Supplementary Figure S59).

### Binding Ability of the Crosslinked Control Polymer

Increasing the amount of pyridine functional groups in the polymers increases binding ability. To support that the binding and removal mechanism is facilitated by hydrogen bonding involving the pyridine groups, a control polymer was synthesized by using **STY** in place of **4VP**. A crosslinked polymer analogous to best performing crosslinked pyridine polymer was synthesized, namely **P(STY**_**3**_ **-EGDMA**_**1.0**_ **-BMA)**. The **STY** control polymer removed minimal **PPL-HCl** (2%, Supplementary Figure S57) and some **2NO** (26%, Supplementary Figure S58). The ability of the **STY** control polymer to bind the contaminants to a lesser extent could be due to favourable hydrophobic interactions. However, the binding ability is significantly reduced when the pyridine groups are absent.

## Small Molecule Cocrystallization

**FIGURE 10:**
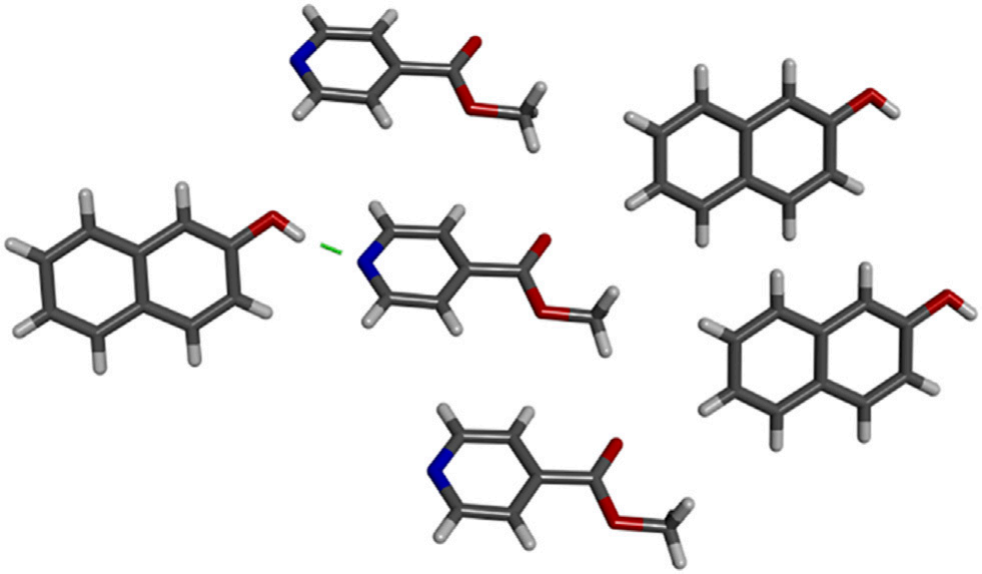
Crystal structure of **2NO·MI** grown from methanol. Hydrogen bond shown with green dashed line.

To further understand the recognition between the contaminants and copolymer that leads to effective removal, cocrystallization of the small molecules **2NO** and methyl isonicotinate (**MI**) was conducted. **MI** contains pyridine and ester functional groups and mimics the functional groups in the polymer backbone. Cocrystallization of **2NO** and **MI** in methanol yielded **2NO‧MI**, and the two components interact through O-H‧‧‧N hydrogen bonds between the phenol of **2NO** and pyridine of **MI**. There was no O-H‧‧‧O (ester) hydrogen bonding observed in the structure. In fact, the carbonyl groups of **MI** are surrounded by either **MI** molecules or the C-H groups of **2NO** (Figure 10 and Supplementary Figure S2). This further supports the idea that hydrogen bonding between the contaminant and pyridine-functionalized polymer is a favorable intermolecular interaction and provides a pathway for increased removal ability. In addition to hydrogen bonding, hydrophobic interactions between the contaminant and polymer backbone could also support binding of these two micropollutants.

## Conclusion

Herein, we described the synthesis of pyridine-functionalized linear and crosslinked polymers that bind and remove **PPL-HCl** and **2NO** from water solutions. The amount of pyridine functional groups in the polymer influences binding abilities. Overall, higher synthetic feeds of **4-VP** lead to improved binding abilities for the polymers. Approximately 20–40% of **PPL-HCl** could be removed from water solutions, and binding abilities for **2NO** from water were much higher at 60–88% over a time frame of 1–4 h. For the contaminant **2NO**, the removal ability of the polymers described here is similar to or higher than other polymer-based materials. A control polymer lacking pyridine groups exhibits significantly lower binding ability than the pyridine polymers. Additionally, small-molecule cocrystallization experiments with a polymer backbone mimic, **MI**, demonstrate that O-H‧‧‧N hydrogen bonding between the phenol of **2NO** and pyridine in **MI** is a favourable intermolecular interaction and supports the high binding ability observed with the pyridine-functionalized polymers. Adsorbents that engage in supramolecular interactions with contaminants have demonstrated promise as micropollutant remediation materials. Rational design of adsorbent materials can help develop design criteria for new adsorbents with high selectivity and rapid removal times.

## Data Availability

The original contributions presented in the study are included in the article/Supplementary Material, further inquiries can be directed to the corresponding author.
