# Supplementary material for: Removal of the Micropollutants Propranolol Hydrochloride and 2-Naphthol From Water by Pyridine-Functionalized Polymers
Source: Front Chem. 2022 Jan 21;9:793870. doi: 10.3389/fchem.2021.793870 (PMC8815703; doi:10.3389/fchem.2021.793870)
Supplement: Supplementary file 1 [file DataSheet1.PDF]

# Supporting Information

**Removal of the Micropollutants Propranolol Hydrochloride and 2-Naphthol from Water by Pyridine-Functionalized Polymers**

Qixuan Zheng, Daniel K. Unruh, and Kristin M. Hutchins*

Department of Chemistry and Biochemistry, Texas Tech University, 1204 Boston Avenue, Lubbock, TX 79409, USA.

*Email: kristin.hutchins@ttu.edu

1. Experimental details Page S1-S6

2. X-ray diffraction information, data table, and structures Page S7-S8

3. NMR data Page S9-S15

4. GPC data Page S16-S17

5. Washing experiment data Page S18

6. Binding experiment data Page S19-S41

7. Reusability of the highest performing crosslinked polymer Page S42

8. HPLC data Page S43-S44

9. Binding experiment results for other micropollutants Page S45

10. References Page S46

## 1. Experimental details

### Materials

Methyl isonicotinate (**MI**), and propranolol hydrochloride (**PPL-HCl**) were purchased from Oakwood Chemical (Columbia Hwy N. Estill, SC, USA). 4-vinylpyridine (**4-VP**) and ethylene glycol dimethylacrylate (**EGDMA**) were purchased from Alfa Aesar (30 Bond Street, Ward Hill, MA 01835, USA). *n*-Butylmethacrylate (**BMA**, 99%, stabilized) and styrene (**STY**, extra pure, stabilized) were purchased from ACROS Organics (New Jersey, USA). Benzoyl peroxide (**BPO**, regent grade, ≥98%), and azobisisobutyronitrile (**AIBN**) were purchased from Sigma-Aldrich Chemical (St. Louis, MO, USA). 2-Naphthol (**2NO**) was purchased from Tokyo Chemical Industry Co., LTD (Toshima, Kita, Tokyo, Japan). Diethyl ether, methanol, toluene, tetrahydrofuran (**THF**), and basic alumina were purchased from Fisher Scientific (Lenexa, KS, USA). Inhibitors in commercial **4-VP**, **EGDMA**, **BMA**, and **STY** were removed by running the commercial compound through a pipette column filled with basic alumina before using them in synthesis. All other chemicals and solvents were used as received.

### Cocrystallization

### Cocrystallization of 2NO and MI

Cocrystals of **2NO·MI** were synthesized by dissolving **2NO** (30 mg, 0.208 mmol) and **MI** (28.5 mg, 0.208 mmol) in methanol. Slow evaporation of the solution was allowed for a period of 12-14 days until single crystals were formed that were suitable for X-ray diffraction.

### Procedures

**<u>Synthesis of linear polymers</u>**[1]:

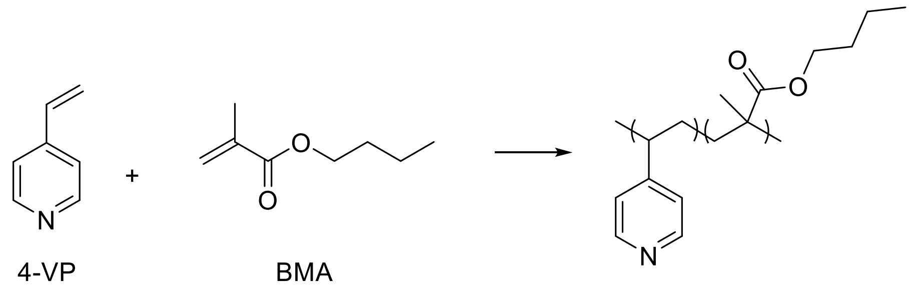


**1:1 4-VP**:**BMA copolymer**: **P(4VP$_1$-co-BMA$_1$)** [poly(4-vinyl pyridine$_1$-co-butylmethacrylate$_1$)]

The linear polymers were synthesized by following a literature procedure,[1] and the specific details are included here. The monomers **4-VP** (3.1542 g, 30 mmol) and **BMA** (4.2660 g, 30 mmol) were added to a 100-mL round bottom flask containing **THF** (30 mL). Then, **AIBN** (0.1641 g, 0.9 mmol) was added to initiate polymerization. The mixture was heated to 60 °C in an oil bath under a nitrogen atmosphere. Polymerization was conducted for 18 hr. The reaction was cooled to room temperature and approximately two-thirds of the **THF** was removed using a rotavap. Diethyl ether was added to

precipitate the copolymer. The copolymer **P(4VP$_1$-co-BMA$_1$)** was then dissolved in methanol (homopolymer **P-BMA** is insoluble in methanol), and the undissolved compounds were removed by filtration. The polymer was re-precipitated with diethyl ether. The precipitate was dissolved in toluene (homopolymer **P-4VP** is insoluble in toluene), and the undissolved compounds were removed by filtration. The polymer was re-precipitated with diethyl ether and isolated. The last two steps (dissolving in methanol and toluene anti-solvents and precipitating) were repeated twice to ensure all unreacted monomers, homopolymers and initiator were removed completely. The product was dried under vacuum overnight to obtain a dry, solid product. Finally, the product was dried under vacuum with heating at 125 °C overnight (5.806 g, 78.2% yield). $^1$H NMR (400 MHz, $CDCl_3$) δ= 8.33 (d, 2H), 6.71, 6.53 (m, 2H), 4.0-2.6 (m, 2H), 2.6-0 (m, 15H). The obtained polymer ratio is 1:0.82 as evidenced by $^1$H NMR spectroscopy. MW results: $M_n$=1248; $M_w$=5776; *Đ*=4.626.

**2:1 4-VP:BMA copolymer**: **P(4VP$_2$-co-BMA$_1$)** [poly(4-vinyl pyridine$_2$-co-butylmethacrylate$_1$)]

The same procedure was used to synthesize the 2:1 copolymer as the 1:1 copolymer; however, the amount of **4-VP** added to the reaction was doubled (**4-VP** = 6.3048 g, 60 mmol). The overall reaction yield was 5.327 g, 71.8% yield. $^1$H NMR (400 MHz, $CDCl_3$) δ=8.36 (d, 4H), 6.59, 6.52, 6.36, 6.23 (m, 4H), 4.0-2.6 (m, 2H), 2.6-0 (m, 15H). The obtained polymer ratio is 2:0.77 as evidenced by $^1$H NMR spectroscopy. MW results $M_n$=13309; $M_w$=23387; *Đ*=1.757.

**3:1 4-VP:BMA copolymer**: **P(4VP$_3$-co-BMA$_1$)** [poly(4-vinyl pyridine$_3$-co-butylmethacrylate$_1$)]

The same procedure was used to synthesize the 3:1 copolymer as the 1:1 copolymer; however, the amount of **4-VP** added to the reaction was tripled (**4-VP** = 9.4626 g, 90 mmol). The overall reaction yield was 5.063 g, 68.2% yield. $^1$H NMR (400 MHz, $CDCl_3$) δ=8.33, 8.26 (d, 6H), 6.66, 6.49, 6.34, 6.20 (m, 6H), 4.0-2.6 (m, 2H), 2.6-0 (m, 15H). The obtained polymer ratio is 3:0.84 as evidenced by $^1$H NMR spectroscopy. MW results $M_n$=11122; $M_w$=18077; *Đ*=1.625.

**3:1 4-VP:BMA copolymer** (increased time): **P(4VP$_3$-co-BMA$_1$)$_t$** [poly(4-vinyl pyridine$_3$-co-butylmethacrylate$_1$)$_t$]

The same procedure was used to synthesize the 3:1 copolymer; however, the reaction time was doubled to increase the molecular weight. The overall reaction yield was 6.153 g, 82.9% yield. $^1$H NMR (400 MHz, $CDCl_3$) δ= 8.33, 8.27 (d, 6H), 6.67, 6.44, 6.34, 6.23 (m, 6H), 4.0-2.6 (m, 2H), 2.6-0 (m, 15H). The obtained polymer ratio is 3:0.88 as evidenced by $^1$H NMR spectroscopy. MW results $M_n$=16973; $M_w$=28680; *Đ*=1.690.

**Synthesis of crosslinked polymers**[2]:

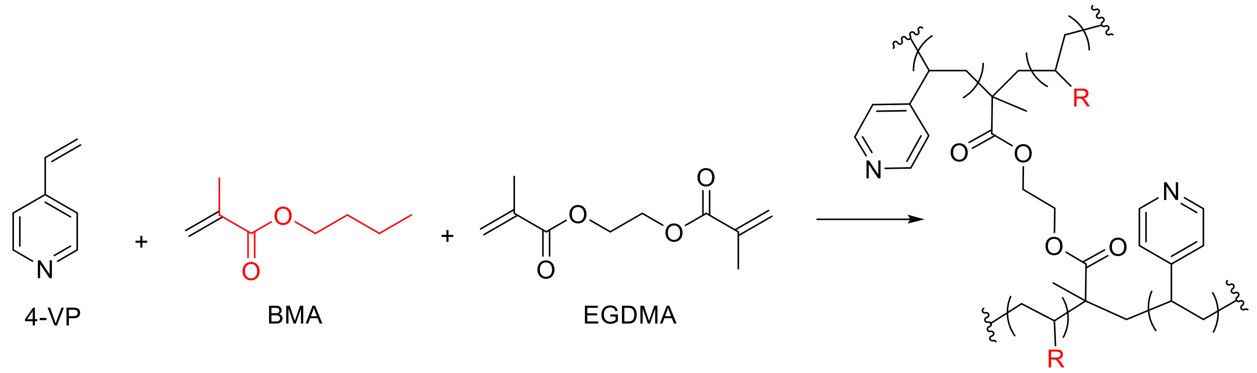


**General procedure:** Deionized water (110 mL), Mowiol 40-88 (0.25 g), **BMA** (22.041 g, 155 mmol), **4-VP** (1.03 g, 9.8 mmol), **EGDMA** (0.1487 g, 0.75 mmol), and **BPO** (Liperox, 0.50 g, 2.1 mmol) were added to a 300-mL three-neck flask. The mixture was stirred at 240 rpm using an IKA 20 digital mechanical stirrer, purged with nitrogen for 15 min, and heated to 70 °C for 12 hr. The particles were isolated via centrifugation at 3000 rpm for three min. The particles were washed using THF (30 mL) and EtOH (70 mL), and centrifuged at 3000 rpm for three min. The washing was repeated an additional four times. The particles were dried in vacuo to give white solid beads. The polymer beads were further purified by Soxhlet extraction using THF at 110 °C overnight. The polymer beads were dried under vacuum heating at 120 °C overnight.

**0.5% EGDMA and single amount of 4-VP crosslinked polymer**: **P(4VP$_1$-EGDMA$_{0.5}$-BMA)** [poly(4-vinyl pyridine$_1$-ethylene glycol dimethylacrylate$_{0.5}$-butylmethacrylate)]

The general procedure above was used to synthesize **P(4VP$_1$-EGDMA$_{0.5}$-BMA)**. The overall reaction yield was 16.482 g, 70.5% yield. $^{13}$C NMR (500 MHz, CDCl$_3$) δ= 177.92, 150.26, 129.21, 123.97, 65.07, 54.65, 45.20, 30.63, 19.76, 14.18.

**1.0% EGDMA and single amount of 4-VP crosslinked polymer**: **P(4VP$_1$-EGDMA$_{1.0}$-BMA)** [poly(4-vinyl pyridine$_1$-ethylene glycol dimethylacrylate$_{1.0}$-butylmethacrylate)]

The general procedure was used to synthesize **P(4VP$_1$-EGDMA$_{1.0}$-BMA)**; however, the amount of **EGDMA** added to the reaction was doubled (**EGDMA** = 0.2973 g, 1.5 mmol). The overall reaction yield was 18.346 g, 78.5% yield. $^{13}$C NMR (500 MHz, CDCl$_3$) δ= 177.54, 134.30, 129.80, 128.87, 64.71, 54.39, 44.70, 30.20, 19.33, 13.72.

**1.0% EGDMA and double amount of 4-VP crosslinked polymer**: **P(4VP$_2$-EGDMA$_{1.0}$-BMA)** [poly(4-vinyl pyridine$_2$-ethylene glycol dimethylacrylate$_{1.0}$-butylmethacrylate)]

The general procedure was used to synthesize **P(4VP$_2$-EGDMA$_{1.0}$-BMA)**; however, the amount of **EGDMA** added to the reaction was doubled (**EGDMA** = 0.2973 g, 1.5 mmol), and the amount of **4-VP** added to the reaction was doubled (2.06 g, 19.6 mmol). The overall reaction yield was 17.653 g, 75.5% yield. $^{13}$C NMR (500 MHz, $CDCl_3$) δ= 177.50, 149.74, 124.22, 123.69, 64.65, 54.13, 44.61, 30.12, 19.24, 13.65.

**1.0% EGDMA and triple amount of 4-VP crosslinked polymer**: **P(4VP$_3$-EGDMA$_{1.0}$-BMA)** [poly(4-vinyl pyridine$_3$-ethylene glycol dimethylacrylate$_{1.0}$-butylmethacrylate)]

The general procedure was used to synthesize **P(4VP$_3$-EGDMA$_{1.0}$-BMA)**. however, the amount of **EGDMA** added to the reaction was doubled (**EGDMA** = 0.2973 g, 1.5 mmol), and the amount of **4-VP** added to the reaction was tripled (3.09 g, 29.4 mmol). The overall reaction yield was 19.884 g, 85.1% yield. $^{13}$C NMR (500 MHz, $CDCl_3$) δ= 177.14, 150.00, 130.04, 123.67, 64.88, 54.43, 44.65, 30.15, 19.27, 13.69.

**2.0% EGDMA and single amount of 4-VP crosslinked polymer**: **P(4VP$_1$-EGDMA$_{2.0}$-BMA)** [poly(4-vinyl pyridine$_1$-ethylene glycol dimethylacrylate$_{2.0}$-butylmethacrylate)]

The general procedure was used to synthesize **P(4VP$_1$-EGDMA$_{2.0}$-BMA)**. However, the amount of **EGDMA** added to the reaction was quadrupled (**EGDMA** = 0.5964 g, 3.0 mmol). The overall reaction yield was 19.135 g, 81.9% yield. $^{13}$C NMR (500 MHz, $CDCl_3$) δ= 176.57, 134.30, 129.81, 128.87, 64.73, 54.37, 45.06, 30.22, 19.34, 13.76.

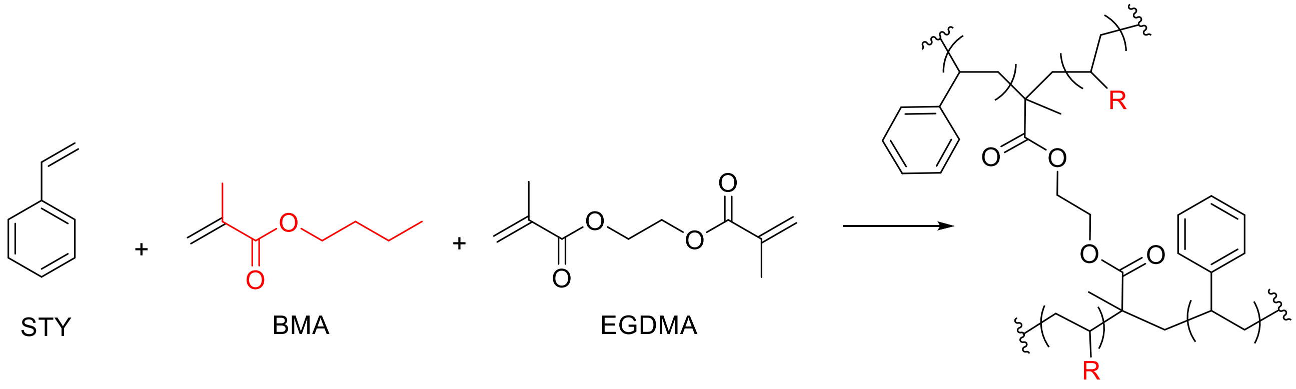


**1.0% EGDMA and triple amount of STY crosslinked polymer (control polymer)**: **P(STY$_3$-EGDMA$_{1.0}$-BMA)** [poly(styrene$_3$-ethylene glycol dimethylacrylate$_{1.0}$-butylmethacrylate)]

Deionized water (110 mL), Mowiol 40-88 (0.25 g), **BMA** (22.041 g, 155mmol), **STY** (3.06 g, 29.4 mmol), **EGDMA** (0.2973 g, 1.5 mmol), and **BPO** (Liperox, 0.50 g, 2.1 mmol) were added to a 300-mL three-neck flask. The mixture was stirred at 240 rpm using an IKA 20 digital mechanical stirrer, purged with nitrogen for 15 min, and heated to 70 °C for 12 hr. The particles were isolated via centrifugation at 3000 rpm for three min. The particles were washed using THF (30 mL) and EtOH (70 mL), and centrifuged at 3000 rpm for three min. The washing was repeated an additional four times.

The particles were dried in vacuo to give white solid beads. The polymer beads were further purified by Soxhlet extraction using THF at 110 °C overnight. The polymer beads were dried under vacuum heating at 120 °C overnight. The overall reaction yield was 21.026 g, 83.3% yield. $^{13}$C NMR (500 MHz, $CDCl_3$) δ= 177.48, 144.98, 128.26, 125.94, 64.65, 54.26, 44.62, 30.15, 19.28, 13.68.

**Washing experiment procedure**

To determine if all unreacted monomer and solvent was removed from the polymer beads, 200 mg of each polymer bead sample (after vacuum drying) and 10 mL of water were added to a vial. The vial was heated for 2 hours at approximately 95 °C while stirring. The solution was filtered to remove the polymer beads. The filtrate was characterized by UV-Vis spectroscopy (Figure S18).

**Binding experiment procedure**

**Linear polymer binding experiment procedure:**

10 mg of the polymer was added to a vial. Then, 2.5 mL propranolol-HCl water solution (0.4 $mmol\cdot L^{-1}$) or 2-naphthol water solution (0.1 $mmol\cdot L^{-1}$) was added to the same vial. The mixture was gently stirred for 15 min, 30 min, 45 min, 1 hr, 2 hr, 3 hr, or 4 hr at room temperature. The solution was filtered through cotton to remove the polymer. For the propranolol-HCl water solutions, 400 μL of filtrate was diluted to 10 mL using a micropipette and 10 mL volumetric flask, and the concentrations after dilution were measured by UV-Vis. For the 2-naphthol water solutions, 500 μL of filtrate was diluted to 5 mL via a micropipette and 5 mL volumetric flask, and the concentrations after dilution were measured by UV-Vis. Control experiments were conducted by filtering the propranolol-HCl water solution and 2-naphthol water solution through cotton at the same dilution. The control experiment is equal to a time of 0 min.

**Crosslinked polymer binding experiment procedure:**

Overnight binding procedure:

200 mg of polymer beads was added to a vial. Then, 2.5 mL propranolol-water solution (0.4 $mmol\cdot L^{-1}$) or 2-naphthol water solution (0.1 $mmol\cdot L^{-1}$) was added to the same vial. The mixture was gently stirred at room temperature for 17 hr. The solution was filtered through cotton to remove the polymer. For the propranolol-HCl water solutions, 400 μL of filtrate was diluted to 10 mL using a micropipette and 10 mL volumetric flask, and the concentrations after dilution were measured by UV-Vis. For the 2-naphthol water solutions, 500 μL of filtrate was diluted to 5 mL via a micropipette and 5 mL volumetric flask, and the concentrations after dilution were measured by UV-Vis. Control experiments were conducted by filtering the propranolol-HCl water solution and 2-naphthol water solution through cotton at the same dilution. The control experiment is equal to a time of 0 min.

Time-dependent binding procedure:

200 mg of polymer beads was added to a vial. Then, 2.5 mL propranolol-water solution (0.4 mmol·$L^{-1}$) or 2-naphthol water solution (0.1 mmol·$L^{-1}$) was added to the same vial. The mixture was gently stirred for 15 min, 30 min, 45 min, 1 hr, 2 hr, 3 hr, or 4 hr at room temperature. The solution was filtered through cotton to remove the polymer. For the propranolol-HCl water solutions, 400 μL of filtrate was diluted 10 mL using a micropipette and 10 mL volumetric flask, and the concentrations after dilution were measured by UV-Vis. For the 2-naphthol water solutions, 400 μL of filtrate was diluted to 5 mL via a micropipette and 5 mL volumetric flask, and the concentrations after dilution were measured by UV-Vis. Control experiments were conducted by filtering the propranolol-HCl water solution and 2-naphthol water solution through cotton at the same dilution. The control experiment is equal to a time of 0 min.

**Reusability of the highest performing crosslinked polymer procedure:**

200 mg of **P(4VP$_3$-EGDMA$_{1.0}$-BMA)** was added to a vial. Then, 2-naphthol water solution (0.1 mmol·$L^{-1}$) was added to the same vial. The mixture was gently stirred for 4 hr at room temperature (i.e. 4 h binding 1$^{st}$ cycle). The solution was filtered through cotton to remove the polymer. 400 μL of filtrate was diluted to 5 mL via a micropipette and 5 mL volumetric flask, and the concentrations after dilution were measured by UV-Vis. Control experiments were conducted by filtering the 2-naphthol water solution through cotton at the same dilution. The control experiment is equal to a time of 0 min. Next, the 200 mg of **P(4VP$_3$-EGDMA$_{1.0}$-BMA)** was collected and purified by Soxhlet extraction using THF at 110 ℃ overnight and subsequently dried under vacuum heating at 120 ℃ overnight. Then, 200 mg of dried **P(4VP$_3$-EGDMA$_{1.0}$-BMA)** was added to a vial. 2-Naphthol water solution (0.1 mmol·$L^{-1}$) was added to the same vial. The mixture was gently stirred for 4 hr at room temperature (i.e. 4 h binding 2$^{nd}$ cycle). The solution was filtered through cotton to remove the polymer. 400 μL of filtrate was diluted to 5 mL via a micropipette and 5 mL volumetric flask, and the concentrations after dilution were measured by UV-Vis. Finally, the UV-Vis data of the 4 h binding 1$^{st}$ and 2$^{nd}$ cycle were compared (Figure S59).

## 2. X-ray diffraction information, data table, and structures

X-ray data were collected on a Rigaku XtaLAB Synergy-i Kappa diffractometer equipped with a PhotonJet-i X-ray source operated at 50 W (50kV, 1 mA) to generate Cu Kα radiation (λ = 1.54178 Å) and a HyPix-6000HE HPC detector. Crystals were transferred from the vial and placed on a glass slide in polyisobutylene. A Zeiss Stemi 305 microscope was used to identify a suitable specimen for X-ray diffraction from a representative sample of the material. The crystal and a small amount of the oil were collected on a MīTiGen cryoloop and transferred to the instrument where it was placed under a cold nitrogen stream (Oxford 700 series) maintained at 100 K throughout the experiment. The sample was optically centered with the aid of a video camera to insure no translations were observed as the crystal was rotated through all positions. After data collection, the unit cell was re-determined using a subset of the full data collection. Intensity data were corrected for Lorentz, polarization, and background effects using the *CrysAlis^Pro^*.[3] A numerical absorption correction was applied based on a Gaussian integration over a multifaceted crystal and followed by a semi-empirical correction for adsorption applied using the program *SCALE3 ABSPACK*.[4] The *SHELX-2014*,[5] series of programs was used for the solution and refinement of the crystal structure. Hydrogen atoms bound to carbon, nitrogen, and oxygen atoms were located in the difference Fourier map and were geometrically constrained using the appropriate AFIX commands.

**Table S1**. X-ray data for cocrystal **2NO·MI**.

| | |
|---|---|
| chemical formula | $C_{17}H_{15}NO_3$ |
| formula mass | 281.30 |
| crystal system | triclinic |
| space group | $P\bar{1}$ |
| a/Å | 5.90540(10) |
| b/Å | 14.8019(2) |
| c/Å | 16.7093(2) |
| α/° | 107.1420(10) |
| β/° | 96.4240(10) |
| γ/° | 91.2630(10) |
| V/Å$^3$ | 1384.60(4) |
| $P_{calc}$/g cm$^{-3}$ | 1.349 |
| T/K | 100.0(3) |
| Z | 4 |
| radiation type | Cu Kα |
| absorption coefficient, | 0.758 |
| no. of reflections measured | 42874 |

| | |
|---|---|
| no. of independent | 4906 |
| no. of reflection (I>2σ(I)) | 4617 |
| $R_{int}$ | 0.0453 |
| $R_1$ ((I>2σ(I)) | 0.0348 |
| wR($F^2$) (I>2σ(I)) | 0.0903 |
| $R_1$ (all data) | 0.0368 |
| wR($F^2$) (all data) | 0.0920 |
| Goodness-of-fit | 1.021 |
| CCDC deposition number | 2103578 |

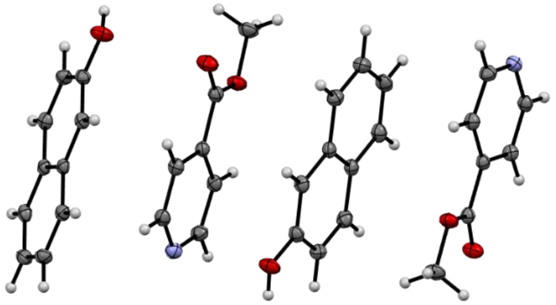

**Figure S1**. Asymmetric unit of **2NO·MI** at 100 K with thermal ellipsoids plotted at 50% probability. Carbon, hydrogen, oxygen, and nitrogen atoms are represented by gray, white, red, and light blue ellipsoids, respectively.

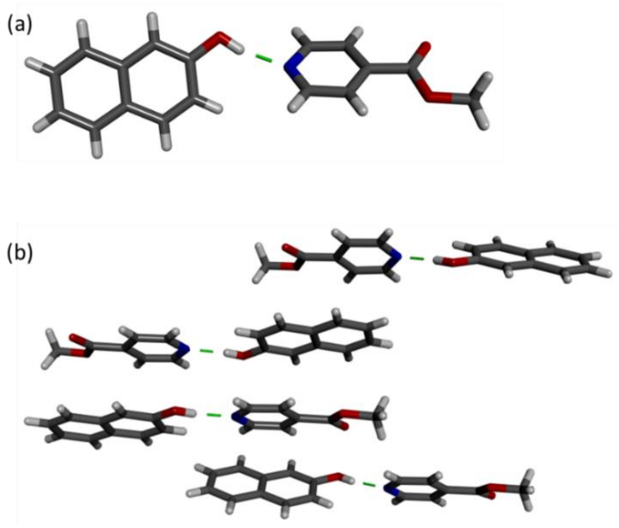


**Figure S2**. Crystal structures of **2NO·MI**: (a) hydrogen-bond interaction and (b) layered two-component hydrogen bonded assemblies. Hydrogen bonds shown with green dashed lines.

## 3. NMR data

**NMR data for cocrystals and linear polymers**: Single crystals from the cocrystallization experiments were removed from the vial and dissolved in $CDCl_3$ for NMR experiments. The linear polymers were dissolved in $CDCl_3$ for NMR experiments. NMR data was collected using a JOEL ECS 400 MHZ Spectrometer.

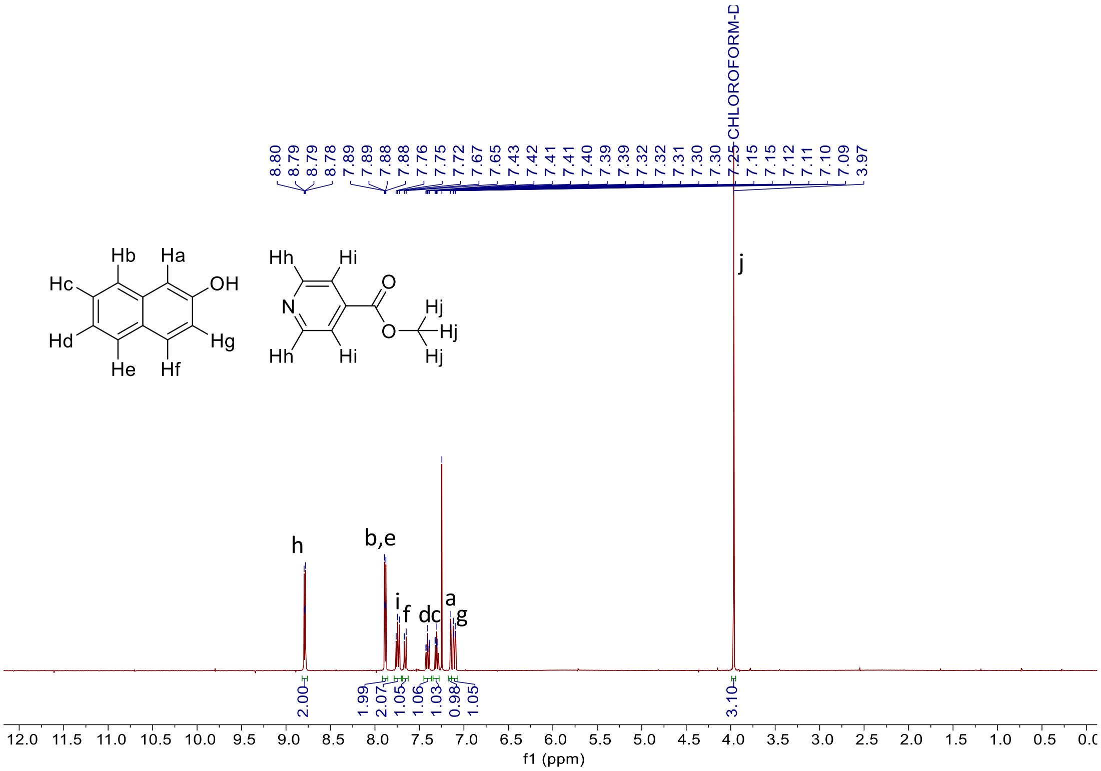


**Figure S3**. $^{1}$H NMR spectrum of cocrystal **2NO·MI**.

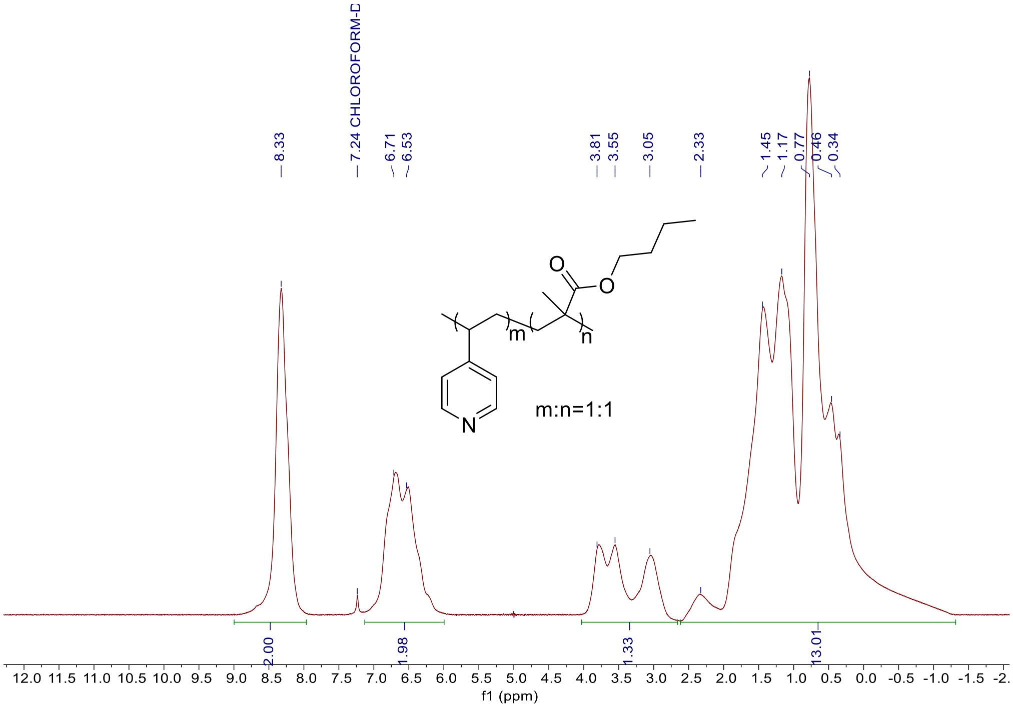


**Figure S4**. ¹H NMR spectrum of **P(4VP$_1$-co-BMA$_1$)**.

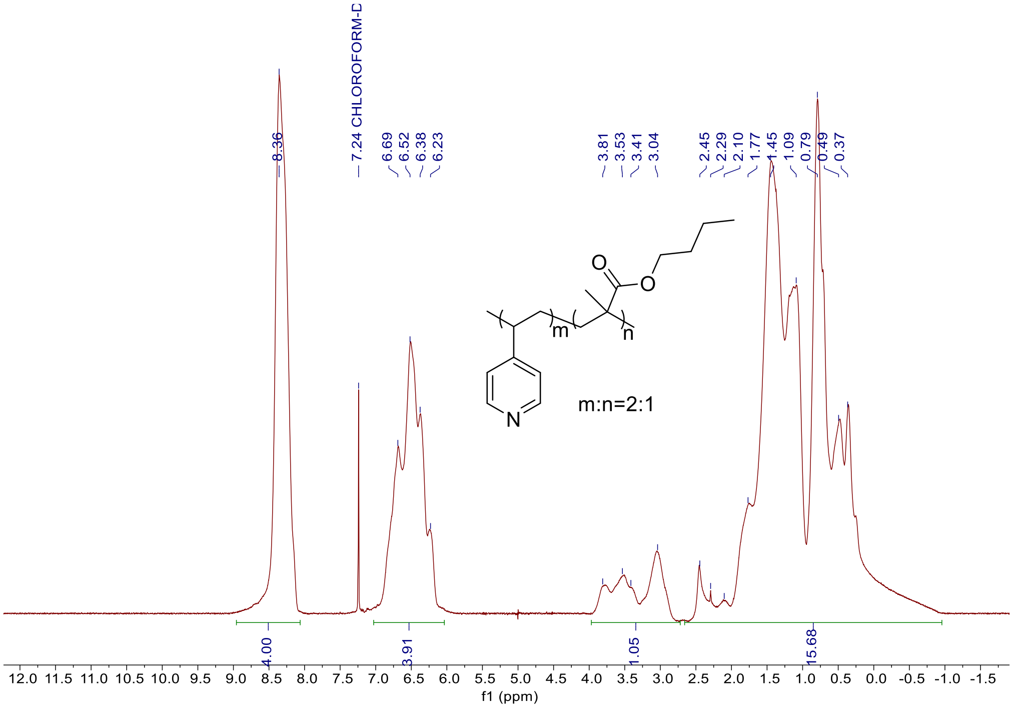


**Figure S5**. ¹H NMR spectrum of **P(4VP$_2$-co-BMA$_1$)**.

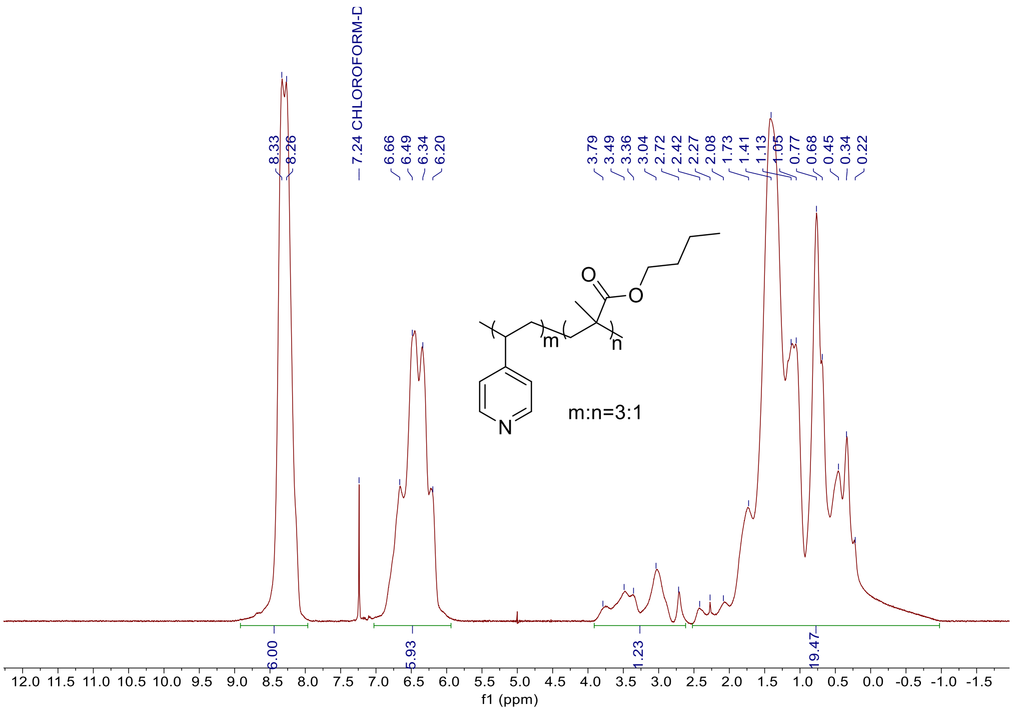


**Figure S6**. $^{1}$H NMR spectrum of **P(4VP$_3$-co-BMA$_1$)**.

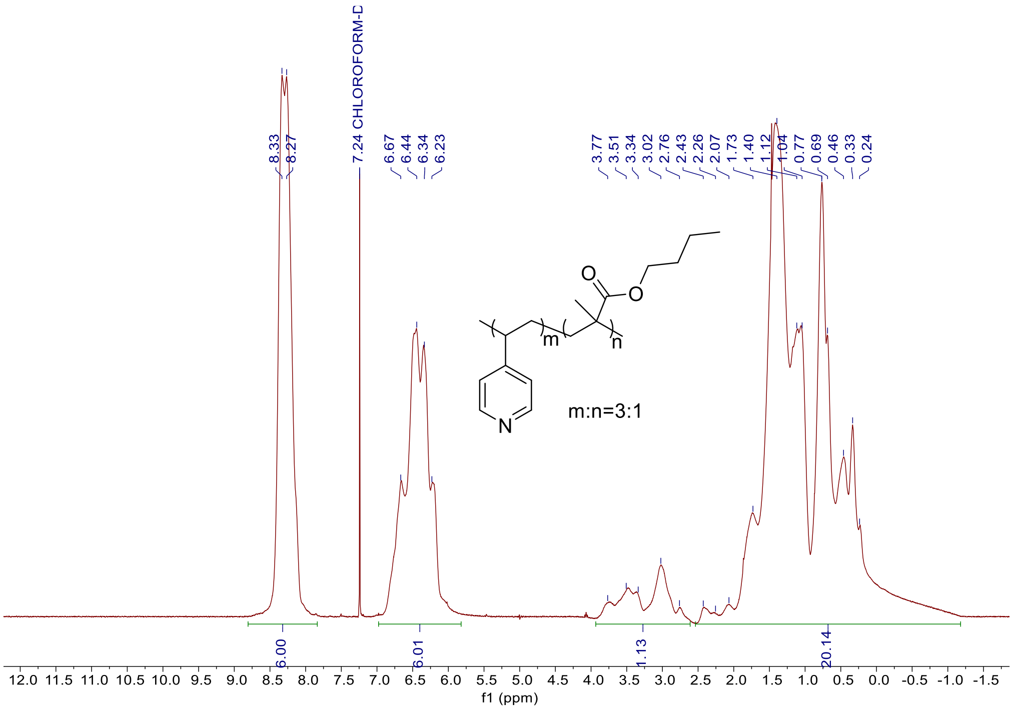


**Figure S7**. $^{1}$H NMR spectrum of **P(4VP$_3$-co-BMA$_1$)$_t$**.

**NMR data for crosslinked polymers**: The crosslinked polymers were swollen in $CDCl_3$ for NMR experiments. The $^{13}C$ NMR spectra with proton decoupling were collected in the gel phase using a Varian Unity Inova 500 MHz Spectrometer. The acquisition time was 0.543 s, and the relaxation delay was 1 s.

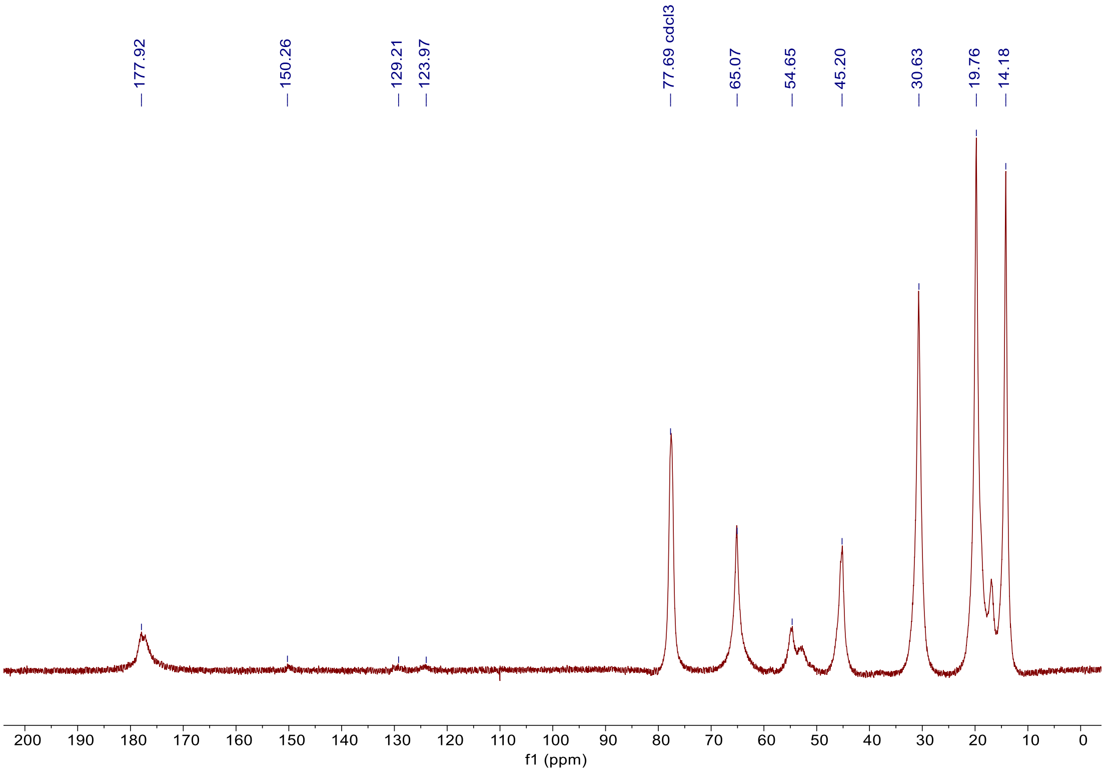


**Figure S8**. $^{13}C$ NMR spectrum of **P(4VP$_1$-EGDMA$_{0.5}$-BMA)**.

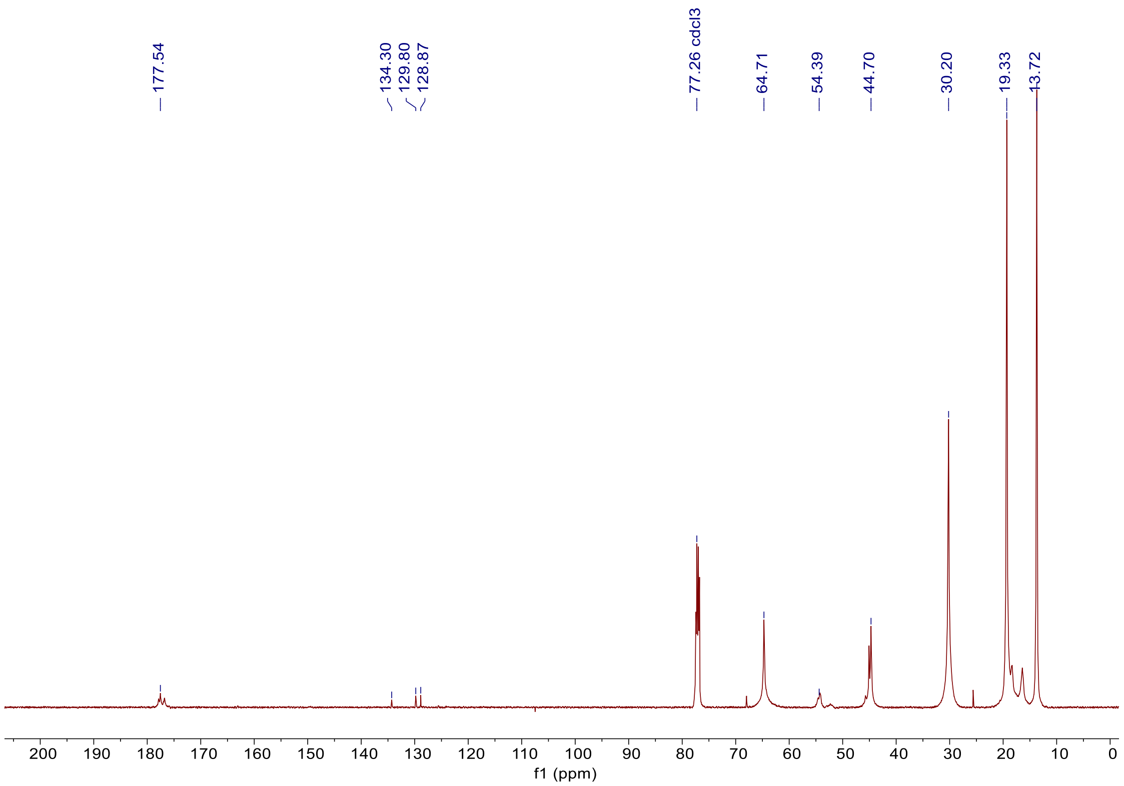


**Figure S9**. $^{13}$C NMR spectrum of **P(4VP$_1$-EGDMA$_{1.0}$-BMA)**.

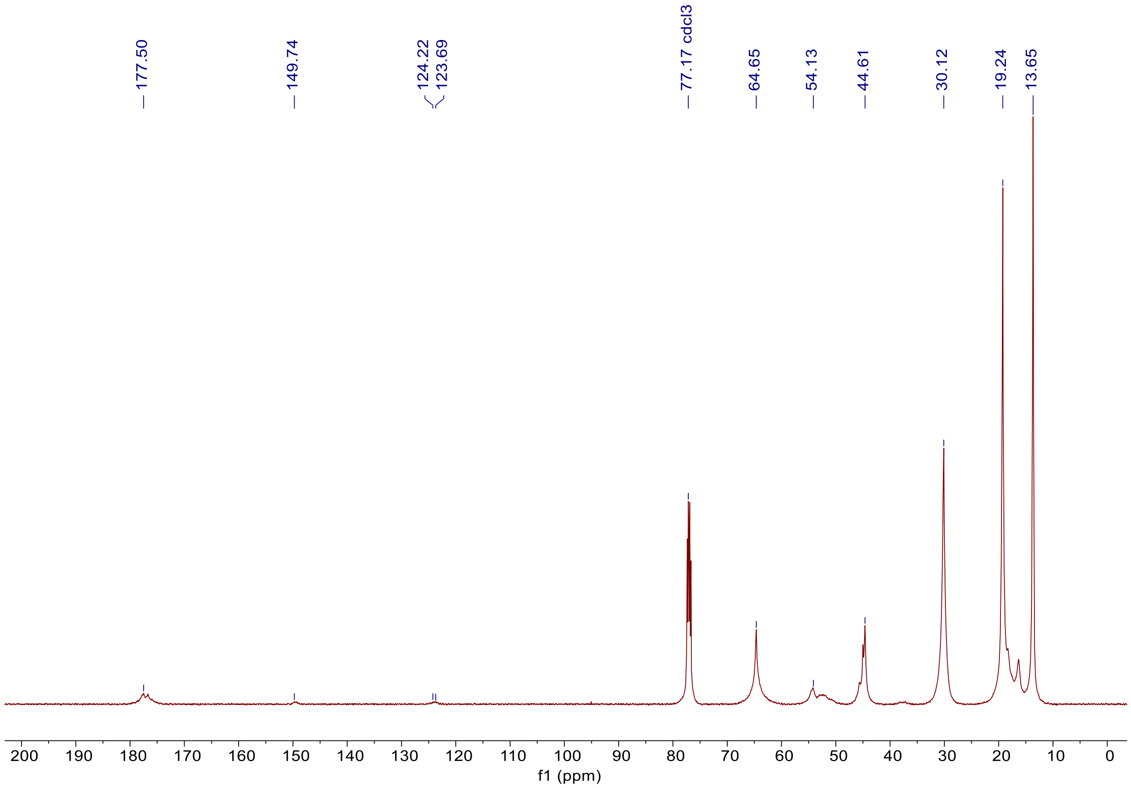


**Figure S10**. $^{13}$C NMR spectrum of **P(4VP$_2$-EGDMA$_{1.0}$-BMA)**.

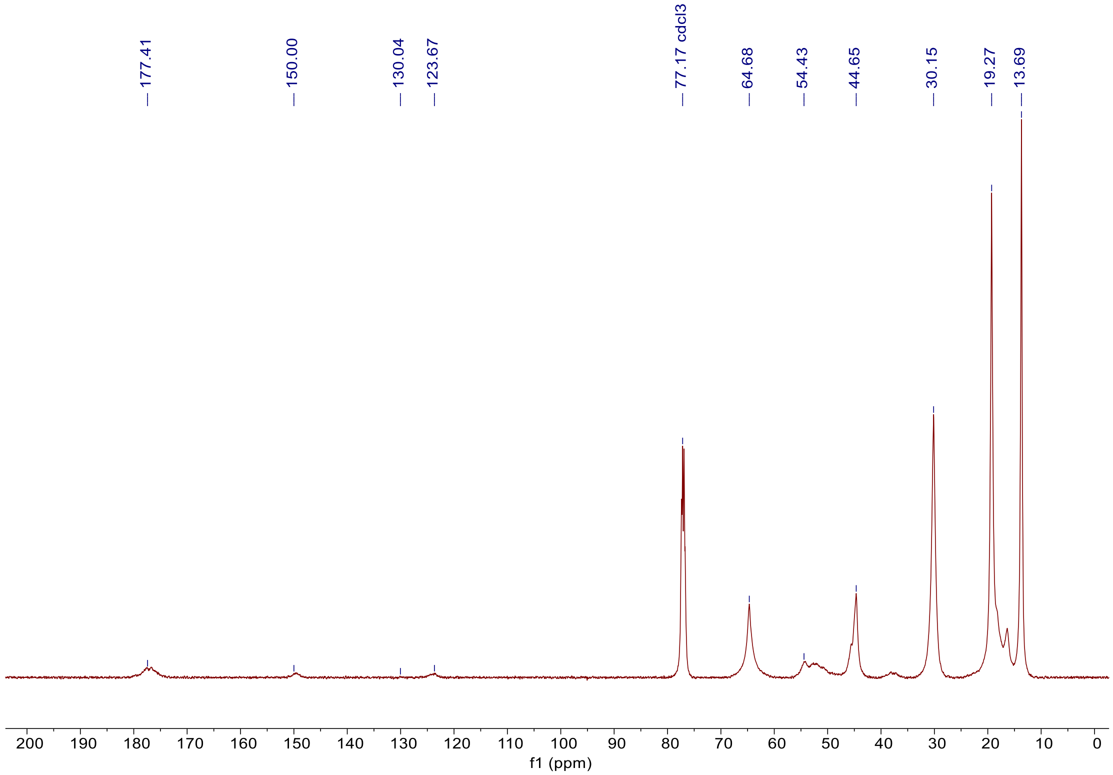


**Figure S11**. $^{13}C$ NMR spectrum of **P(4VP$_3$-EGDMA$_{1.0}$-BMA)**.

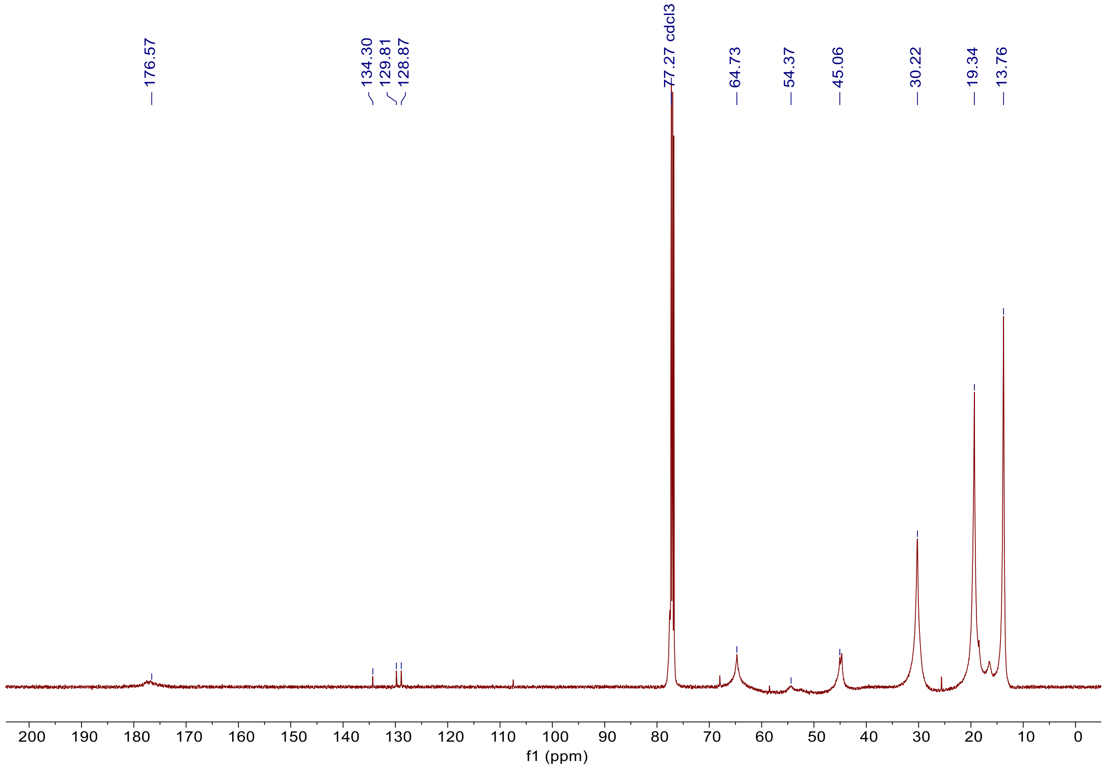


**Figure S12**. $^{13}C$ NMR spectrum of **P(4VP$_1$-EGDMA$_{2.0}$-BMA)**.

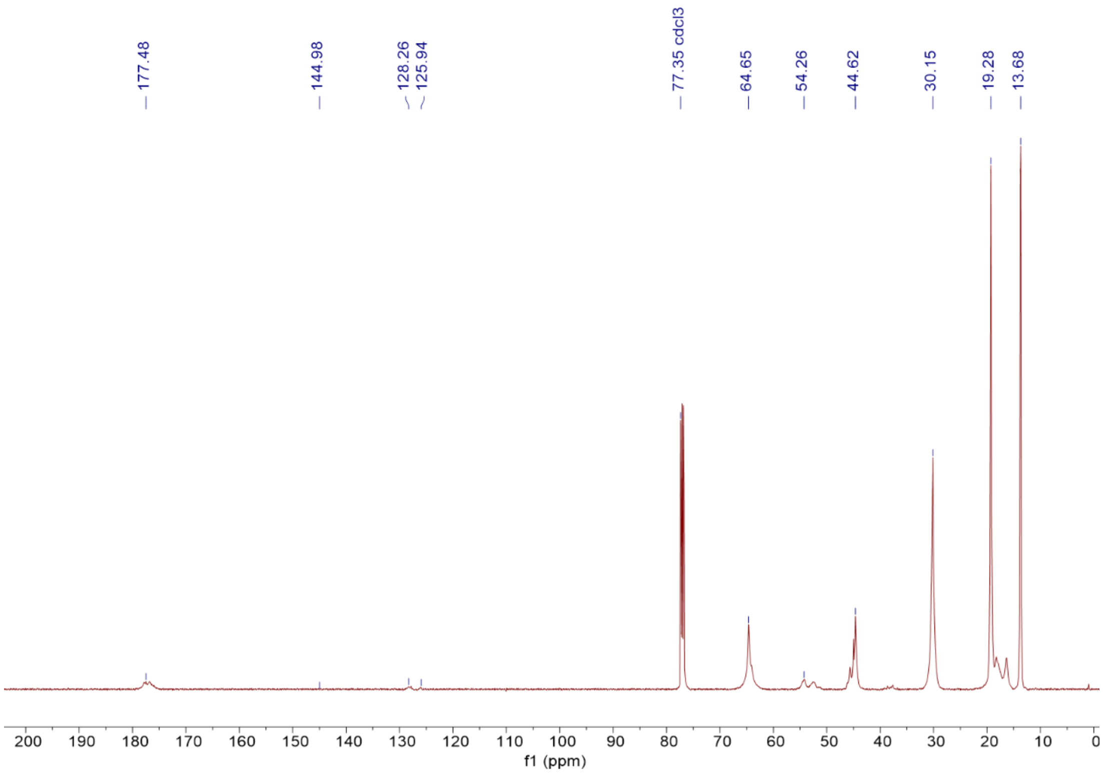


**Figure S13**. $^{13}$C NMR spectrum of control polymer **P(STY$_3$-EGDMA$_{1.0}$-BMA)**.

## 4. GPC data

GPC data was collected on a Tosoh EcoSEC HLC-8320GPC. HPLC grade THF was used as both the mobile phase in the GPC instrument, and the solvent to dissolve the linear polymers. Polystyrene standards were used for calibration.

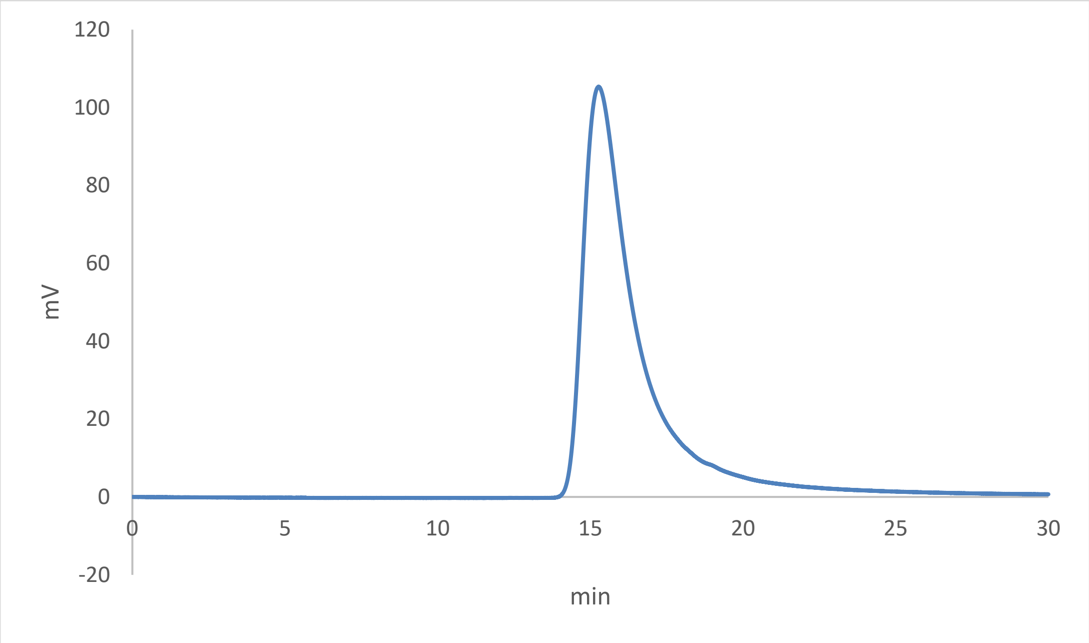


**Figure S14.** GPC chromatogram for **P(4VP$_1$-co-BMA$_1$)**.

$M_n$=1248; $M_w$=5776; *Đ*=4.626.

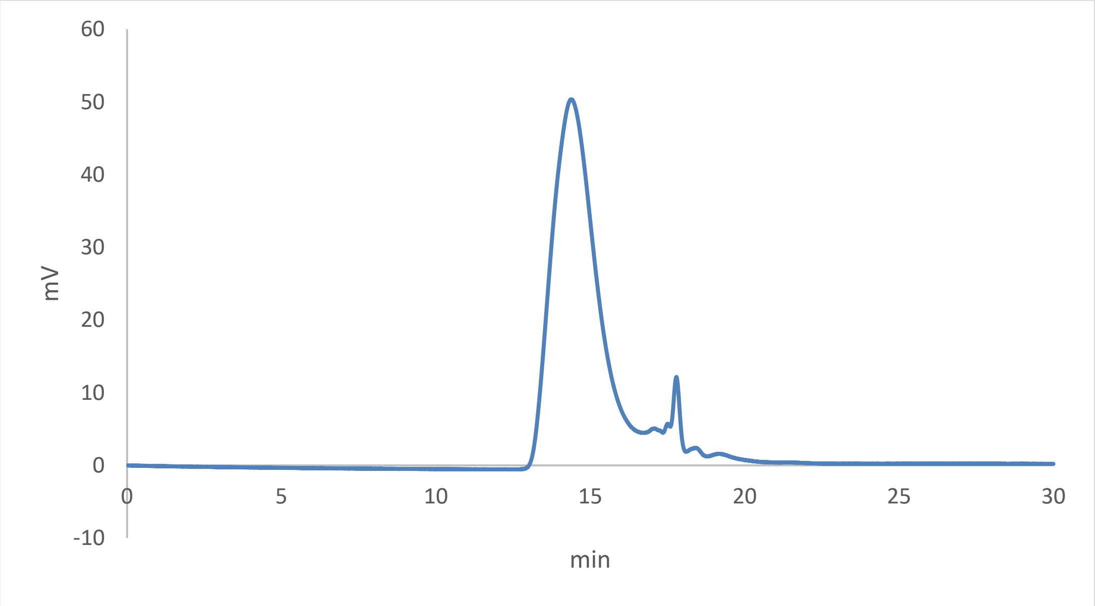


**Figure S15.** GPC chromatogram for **P(4VP$_2$-co-BMA$_1$)**.

$M_n$=13309; $M_w$=23387; *Đ*=1.757.

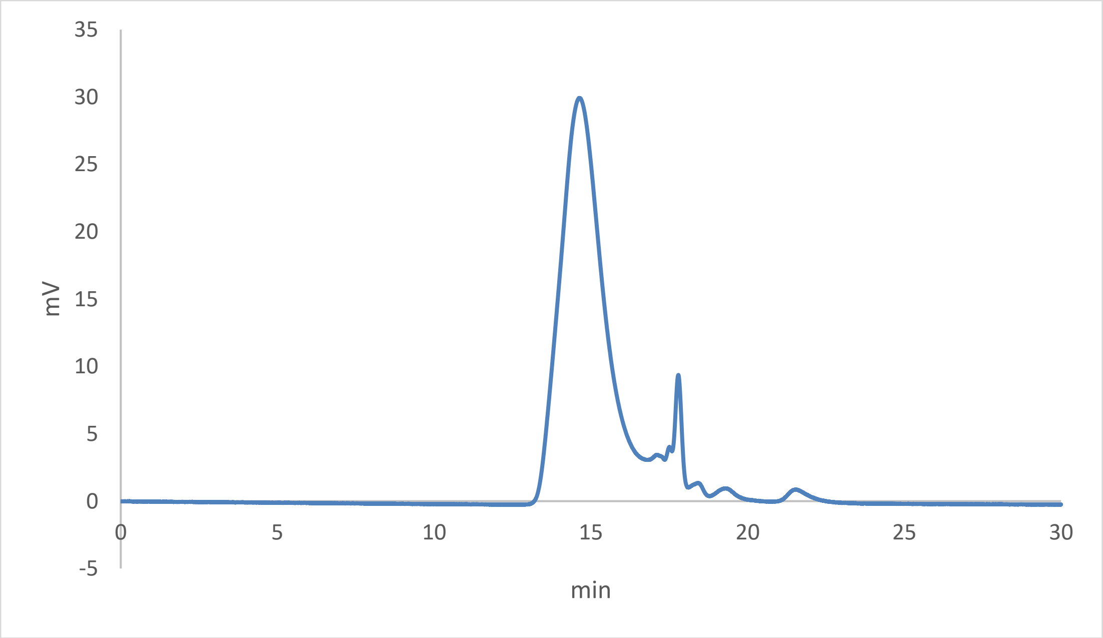


**Figure S16.** GPC chromatogram for **P(4VP$_3$-co-BMA$_1$)**.

$M_n$=11122; $M_w$=18077; $Đ$=1.625.

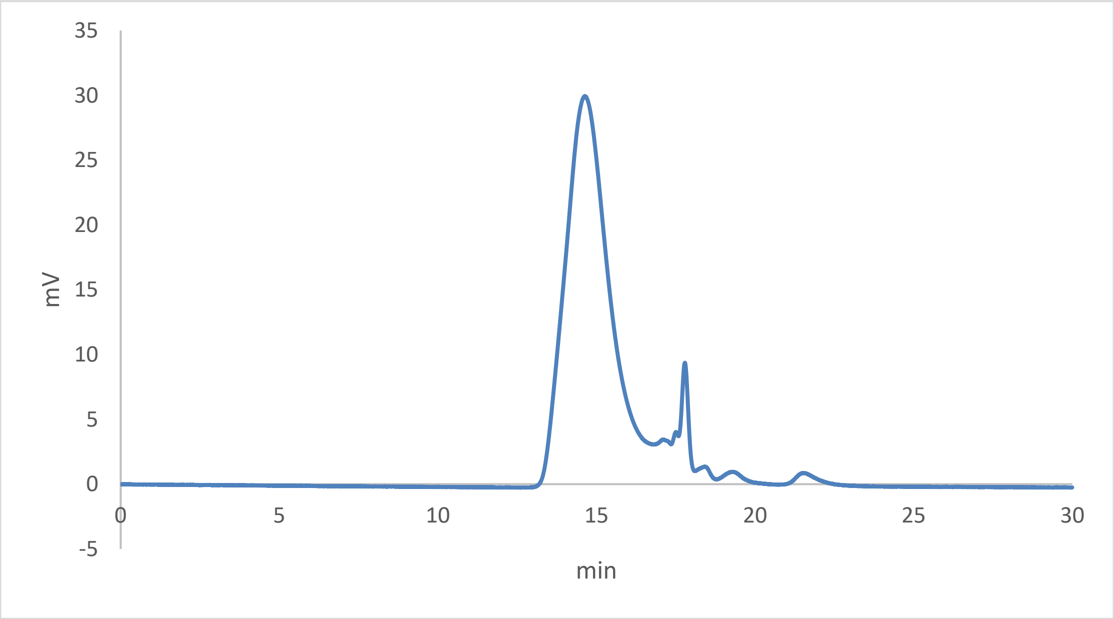


**Figure S17.** GPC chromatogram for **P(4VP$_3$-co-BMA$_1$)$_t$**.

$M_n$=16973; $M_w$=28680; $Đ$=1.690.

## 5. Washing experiments data

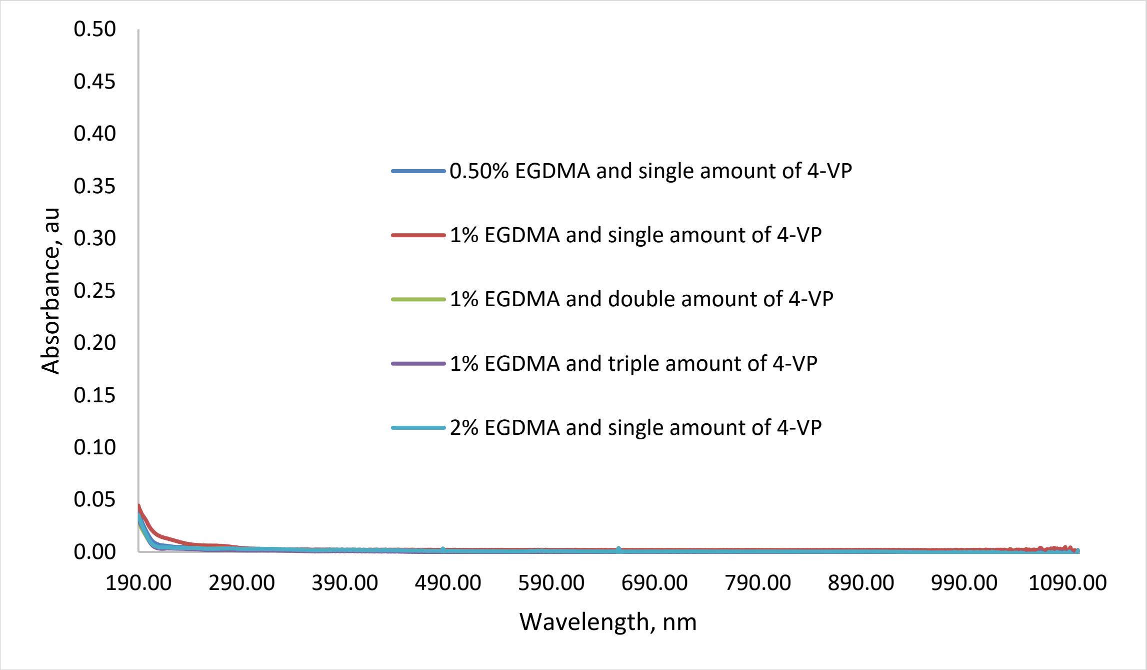


**Figure S18**. UV-Vis spectra following washing experiments for crosslinked polymers.

## 6. Binding experiments data

UV-Vis spectra were collected on an Agilent 8453 UV-Visible Spectroscopy system. The control experiment within each experiment is equal to a time of 0 min. To calculate the removal of the contaminants, the signal at 215 nm is used for **PPL-HCl**, and the signal at 224 nm is used for **2NO**.

**<u>Binding data for linear polymers with PPL-HCl water solution (Figures S19-S26)</u>:**

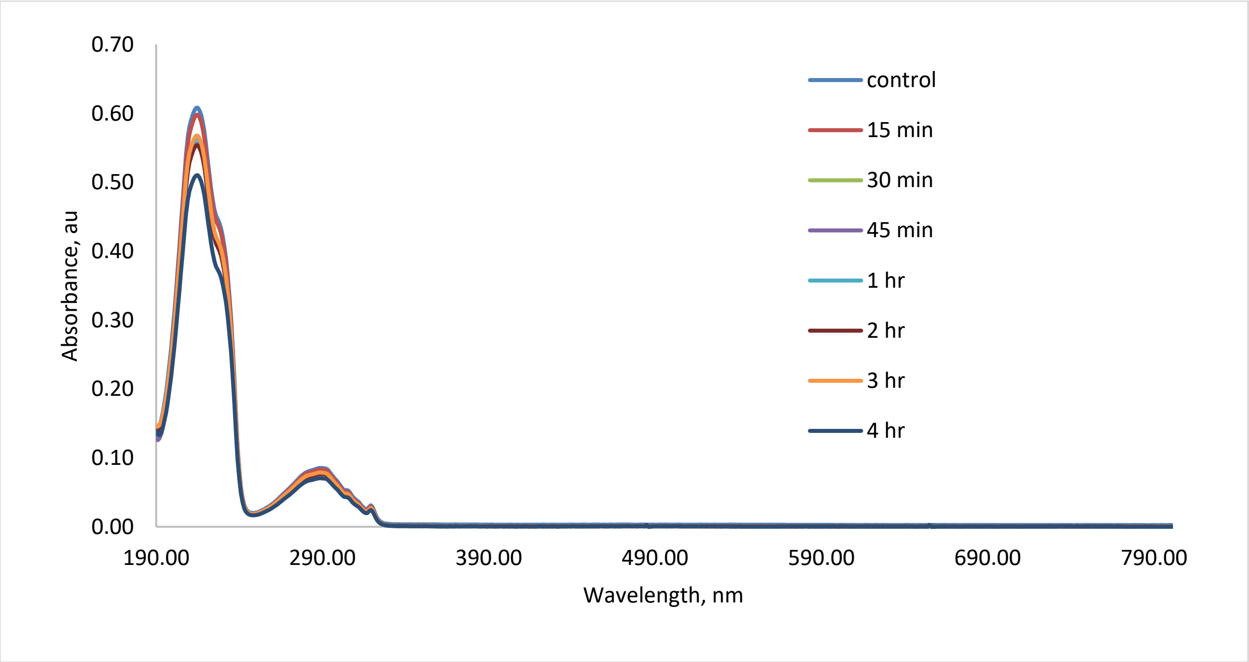


**Figure S19**. UV-Vis spectra of time-dependent binding experiment for **P(4VP$_1$-co-BMA$_1$)**.

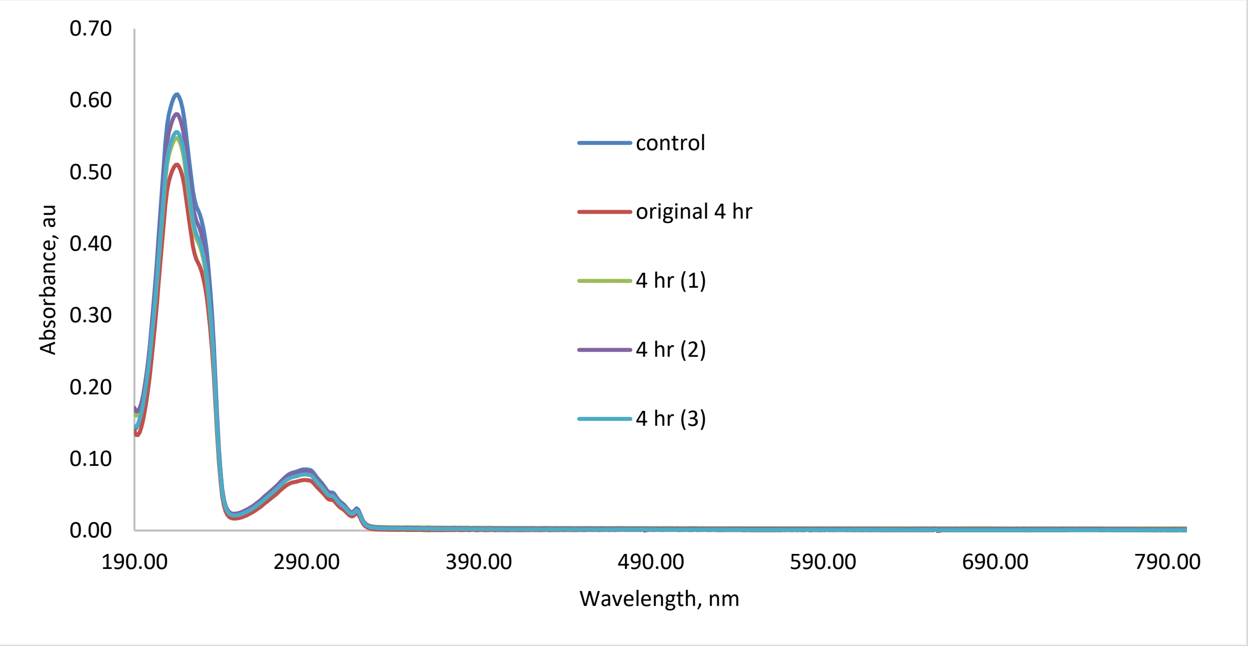


**Figure S20**. UV-Vis spectra for **P(4VP$_1$-co-BMA$_1$)** showing three additional trials at 4 hr binding time. Standard deviation: 0.04.

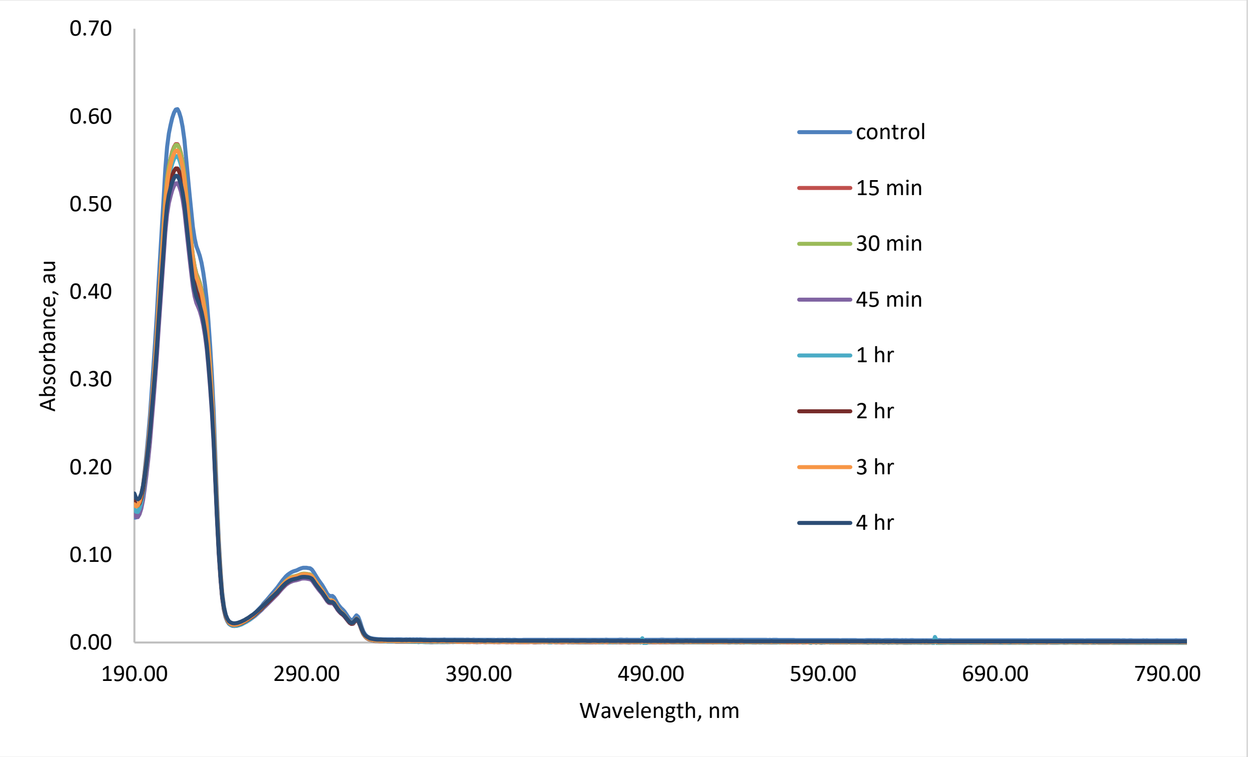


**Figure S21**. UV-Vis spectra of time-dependent binding experiment for **P(4VP$_2$-co-BMA$_1$)**.

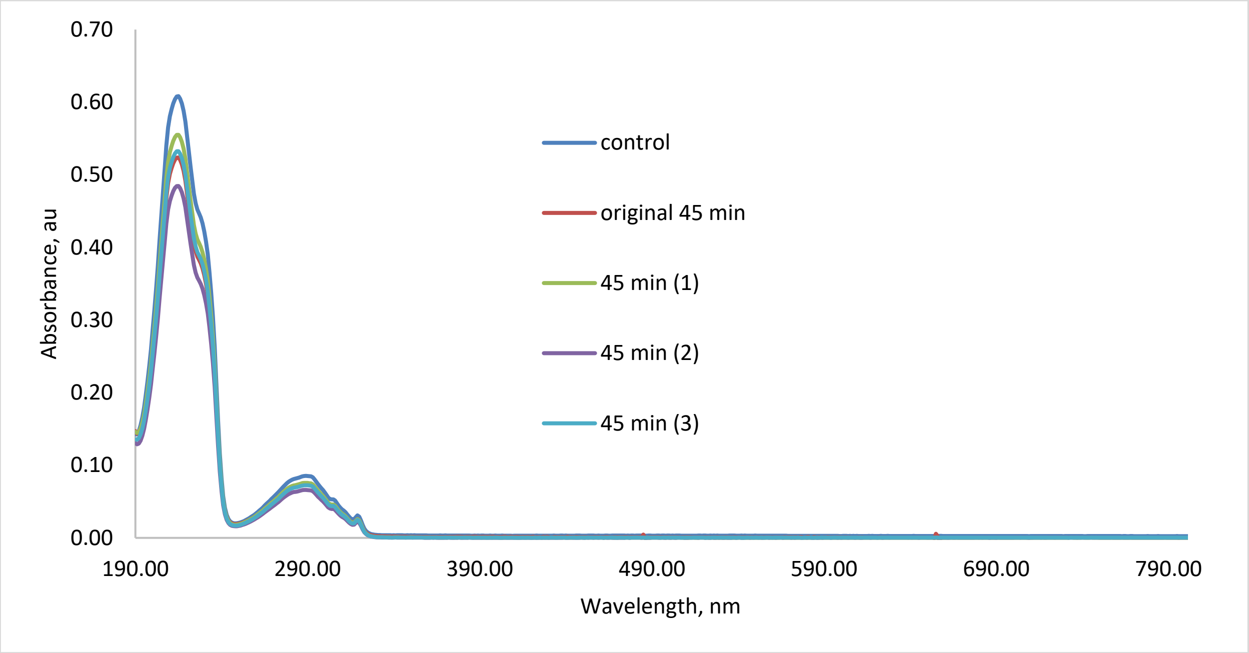


**Figure S22**. UV-Vis spectra for **P(4VP$_2$-co-BMA$_1$)** showing three additional trials at 45 min binding time. Standard deviation: 0.05.

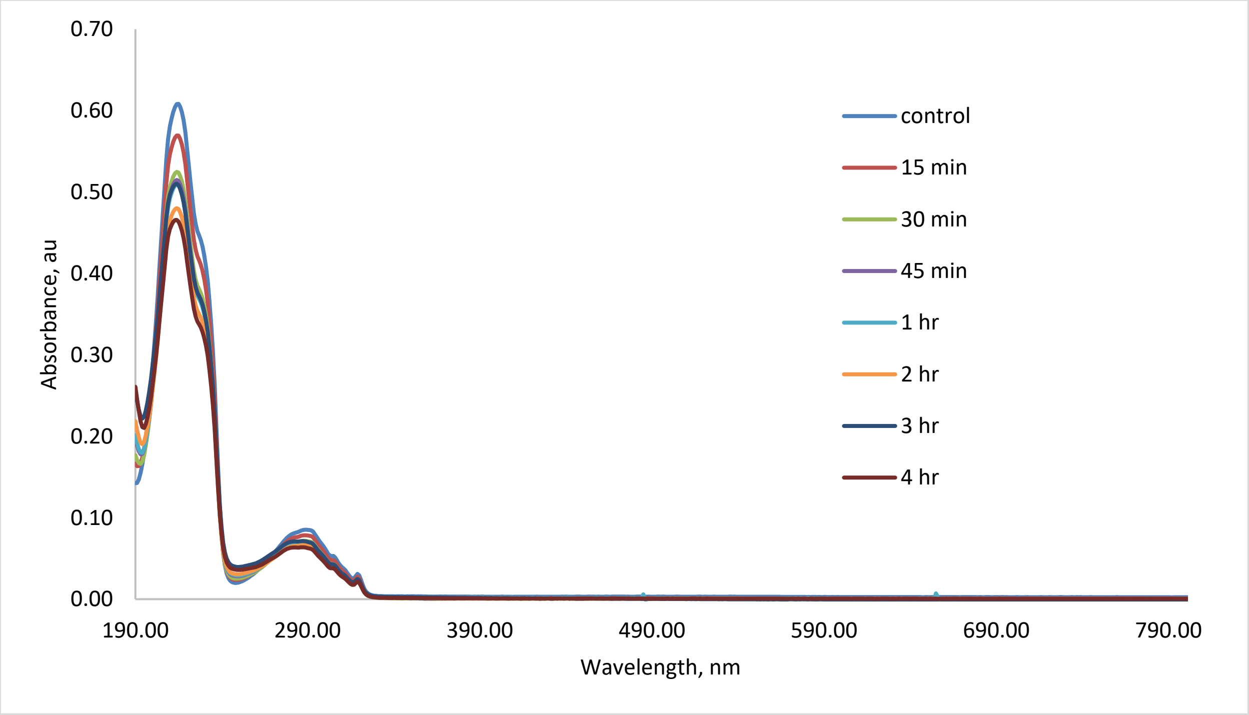


**Figure S23**. UV-Vis spectra of time-dependent binding experiment for **P(4VP$_3$-co-BMA$_1$)**.

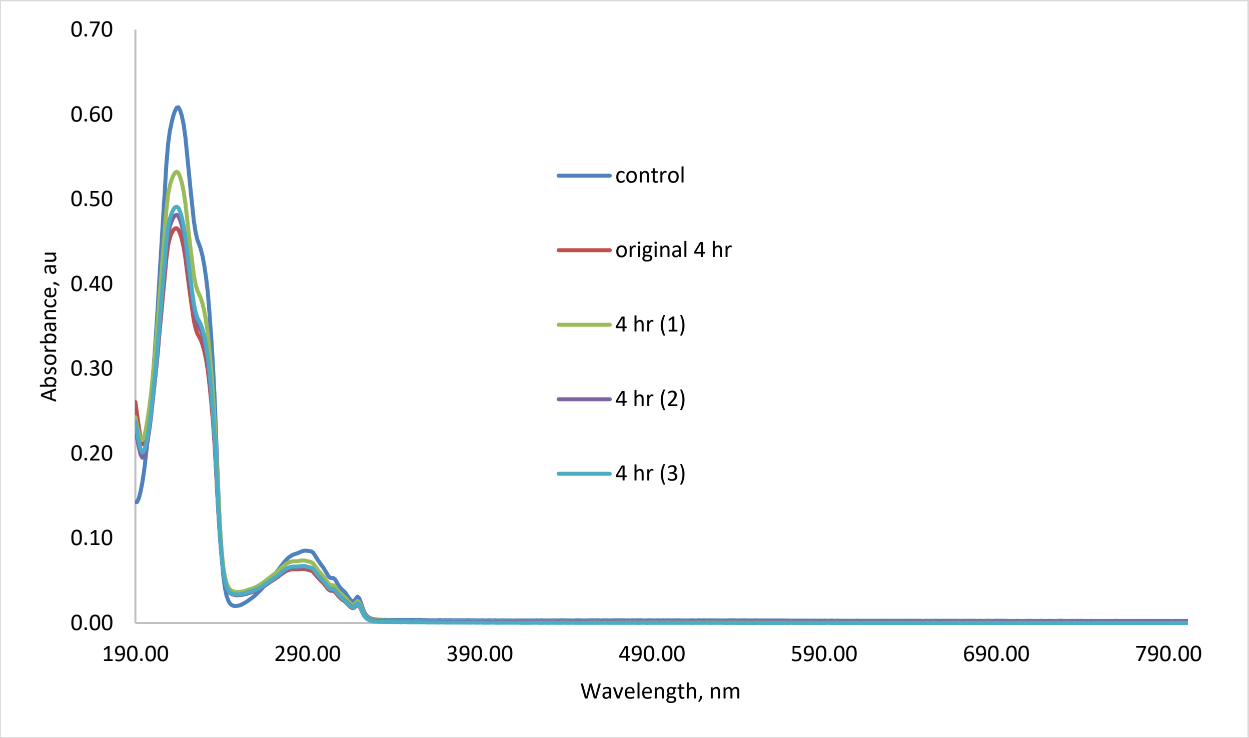


**Figure S24**. UV-Vis spectra for **P(4VP$_3$-co-BMA$_1$)** showing three additional trials at 4 hr binding time. Standard deviation: 0.04.

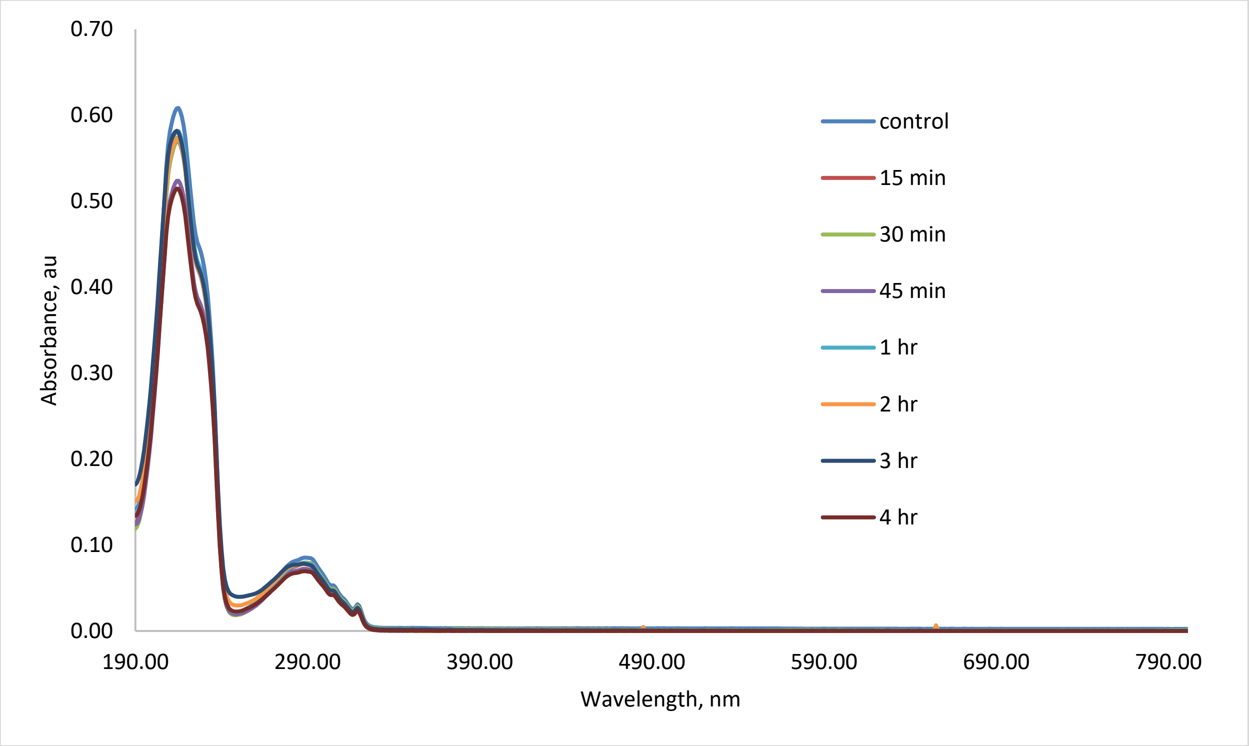


**Figure S25**. UV-Vis spectra of time-dependent binding experiment for **P(4VP$_3$-co-BMA$_1$)$_t$**.

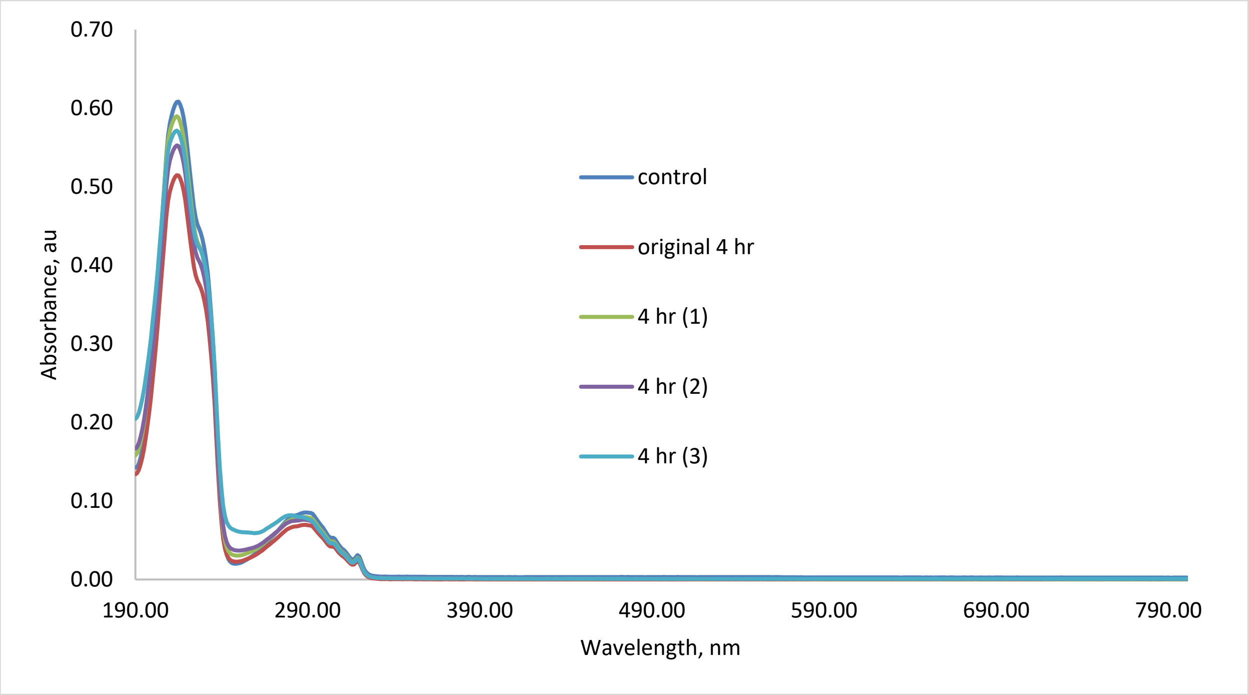


**Figure S26**. UV-Vis spectra for **P(4VP$_3$-co-BMA$_1$)$_t$** showing three additional trials at 4 hr binding time. Standard deviation: 0.06.

**<u>Binding data for linear polymers with 2NO water solution (Figures S27-S34)</u>:**

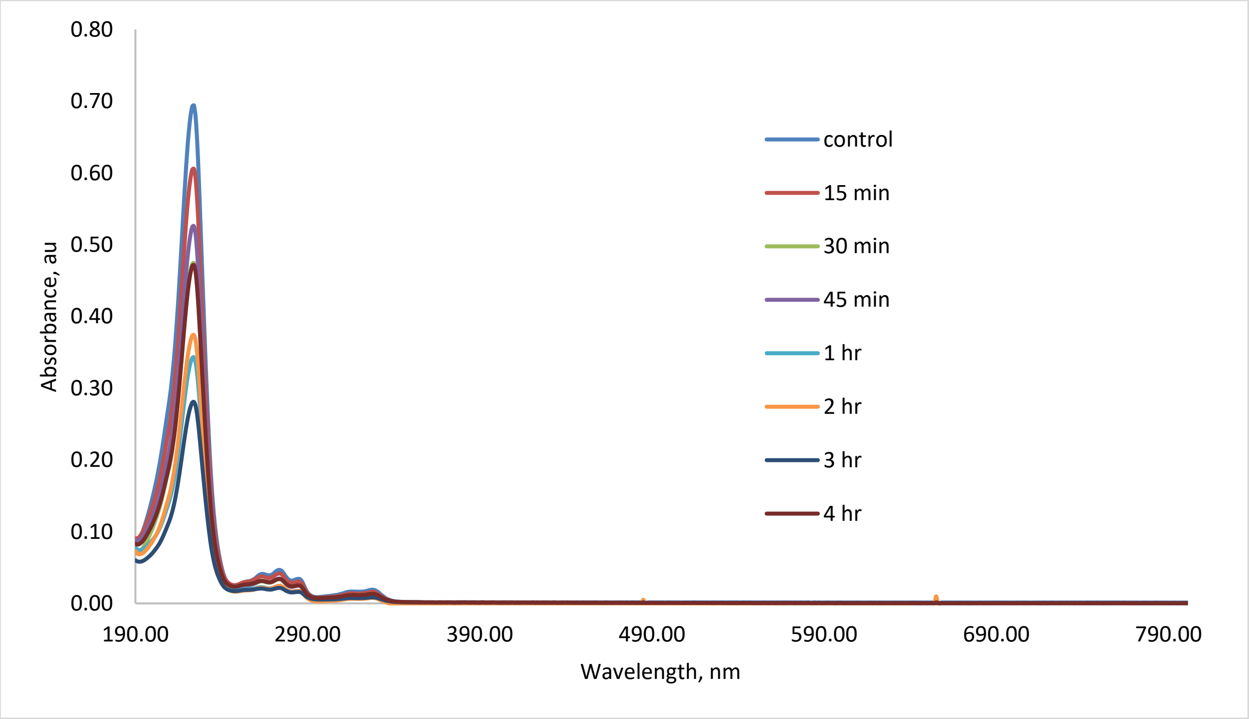


**Figure S27**. UV-Vis spectra of time-dependent binding experiment for **P(4VP$_1$-co-BMA$_1$)**.

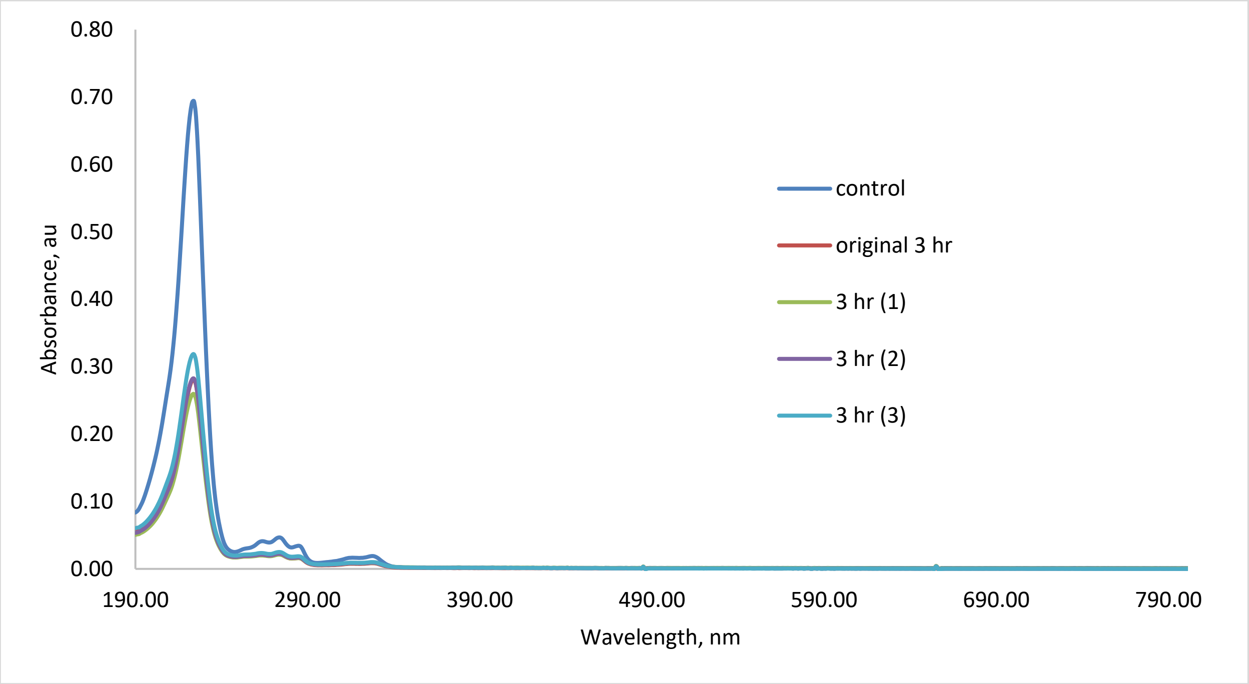


**Figure S28**. UV-Vis spectra for **P(4VP$_1$-co-BMA$_1$)** showing three additional trials at 3 hr binding time. Standard deviation: 0.04.

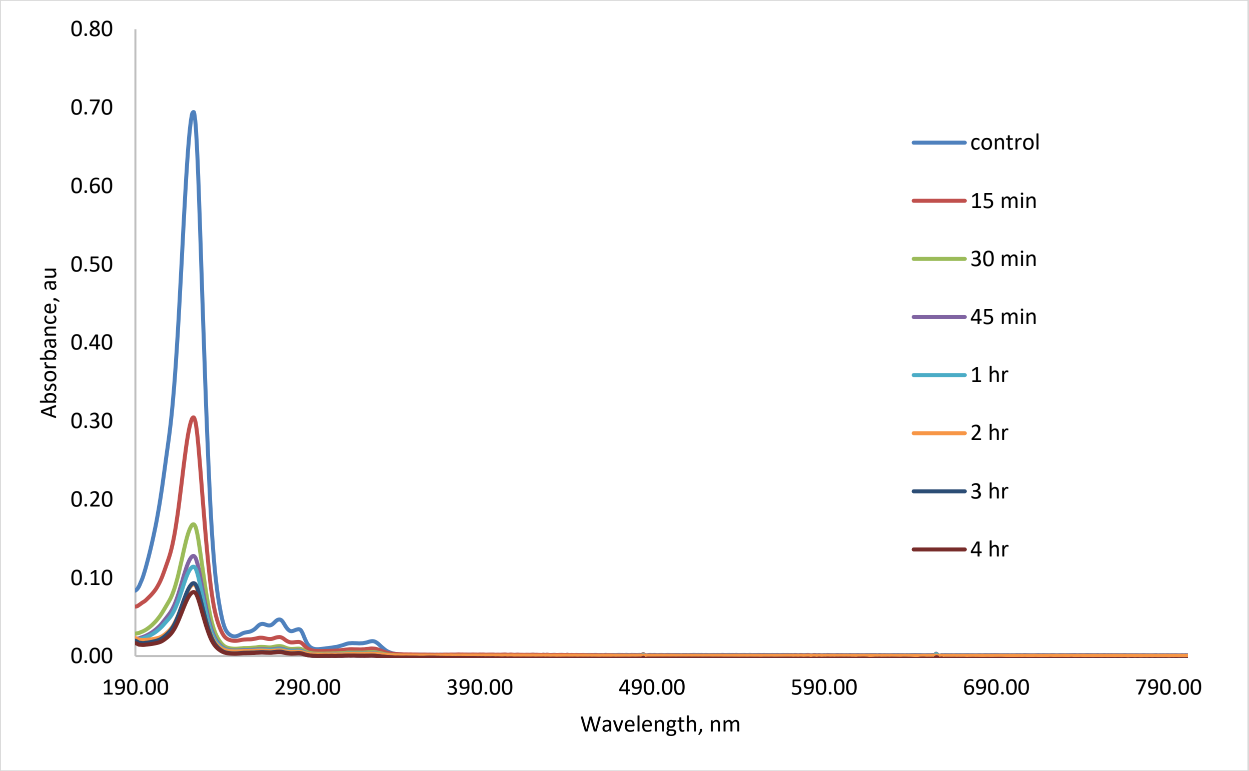


**Figure S29**. UV-Vis spectra of time-dependent binding experiment for **P(4VP$_2$-co-BMA$_1$)**.

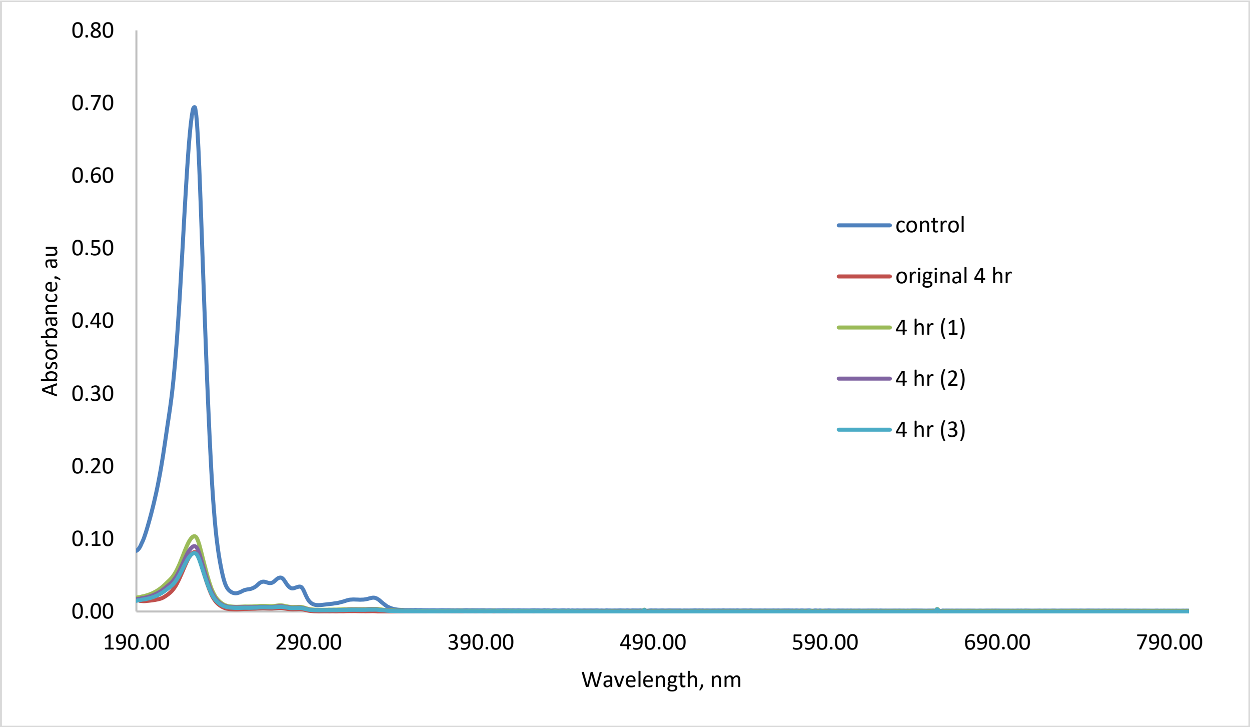


**Figure S30**. UV-Vis spectra for **P(4VP$_2$-co-BMA$_1$)** showing three additional trials at 4 hr binding time. Standard deviation: 0.01.

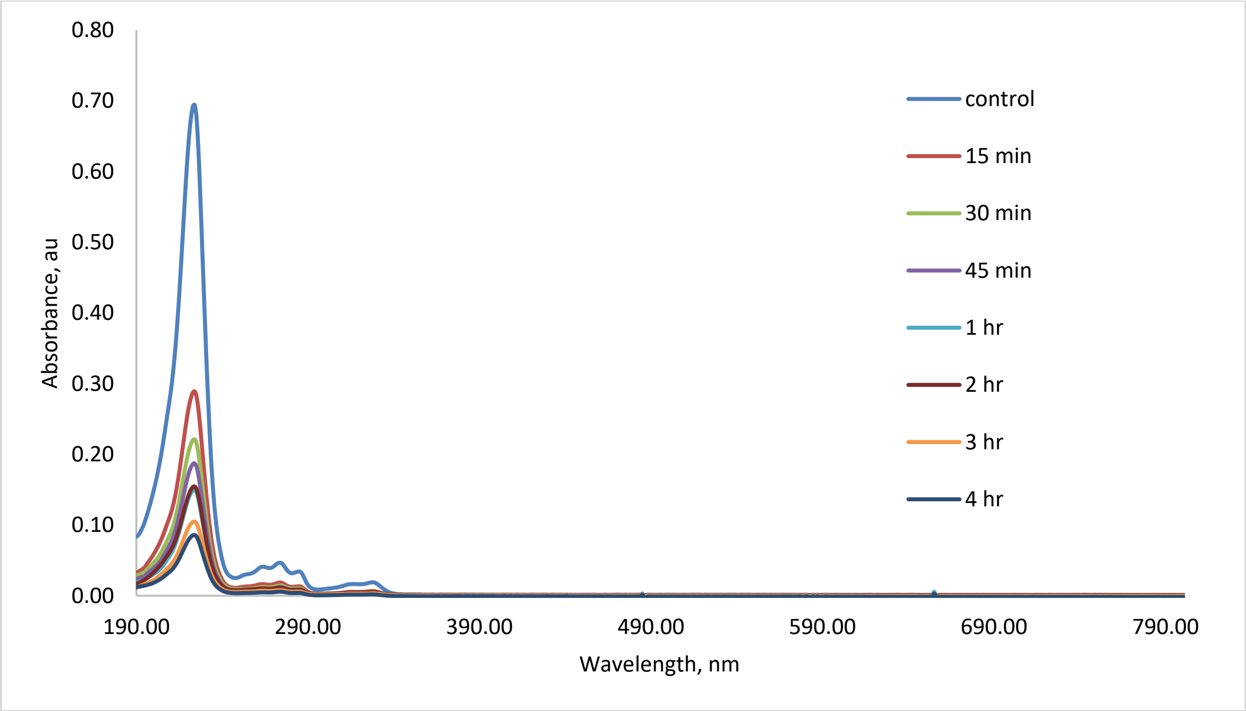


**Figure S31**. UV-Vis spectra of time-dependent binding experiment for **P(4VP$_3$-co-BMA$_1$)**.

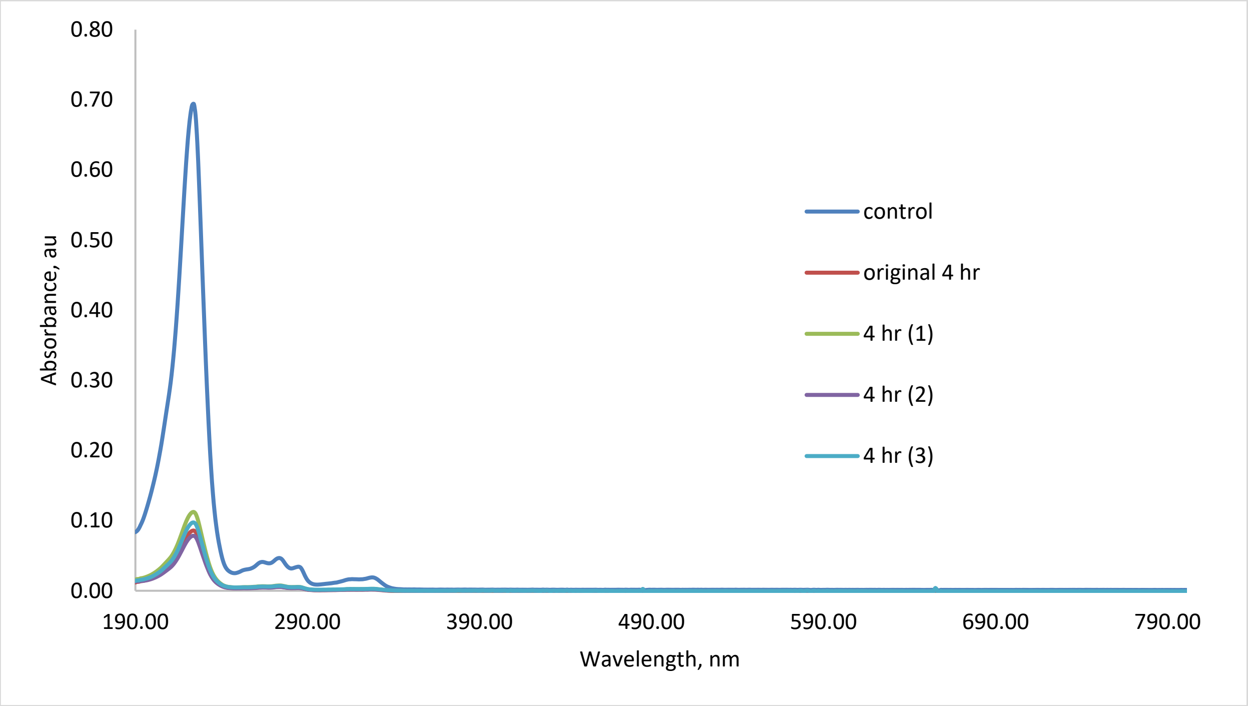


**Figure S32**. UV-Vis spectra for **P(4VP$_3$-co-BMA$_1$)** showing three additional trials at 4 hr binding time. Standard deviation: 0.02.

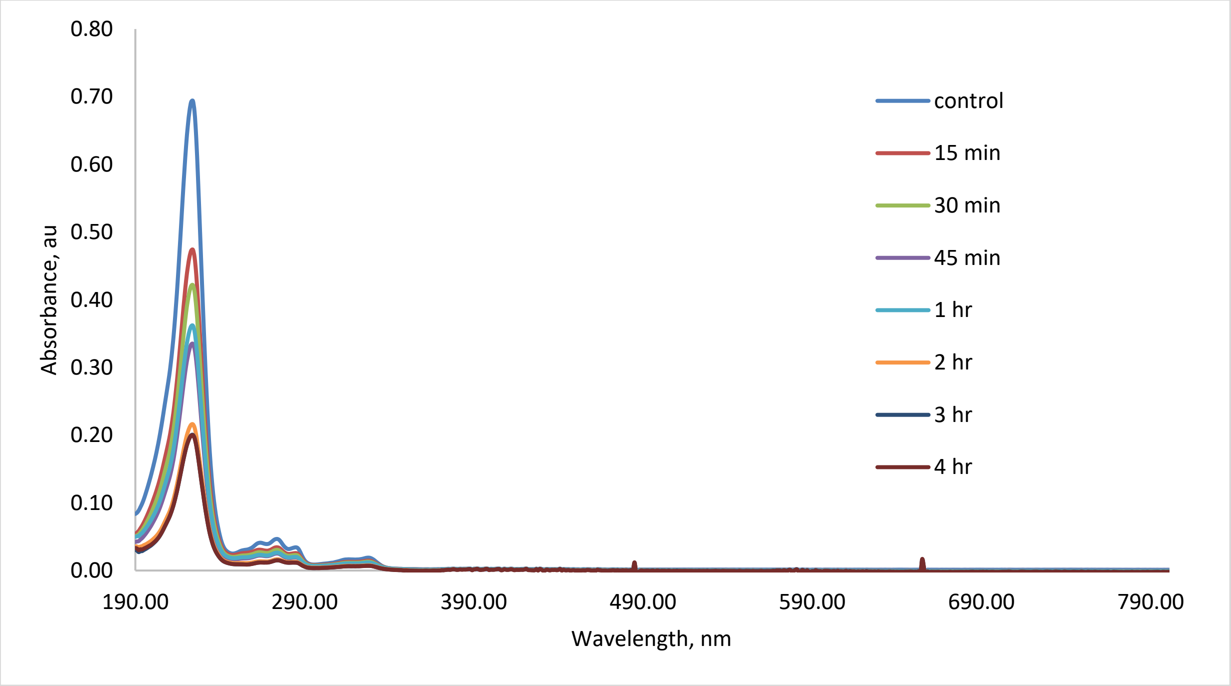


**Figure S33**. UV-Vis spectra of time-dependent binding experiment for **P(4VP$_3$-co-BMA$_1$)$_t$**.

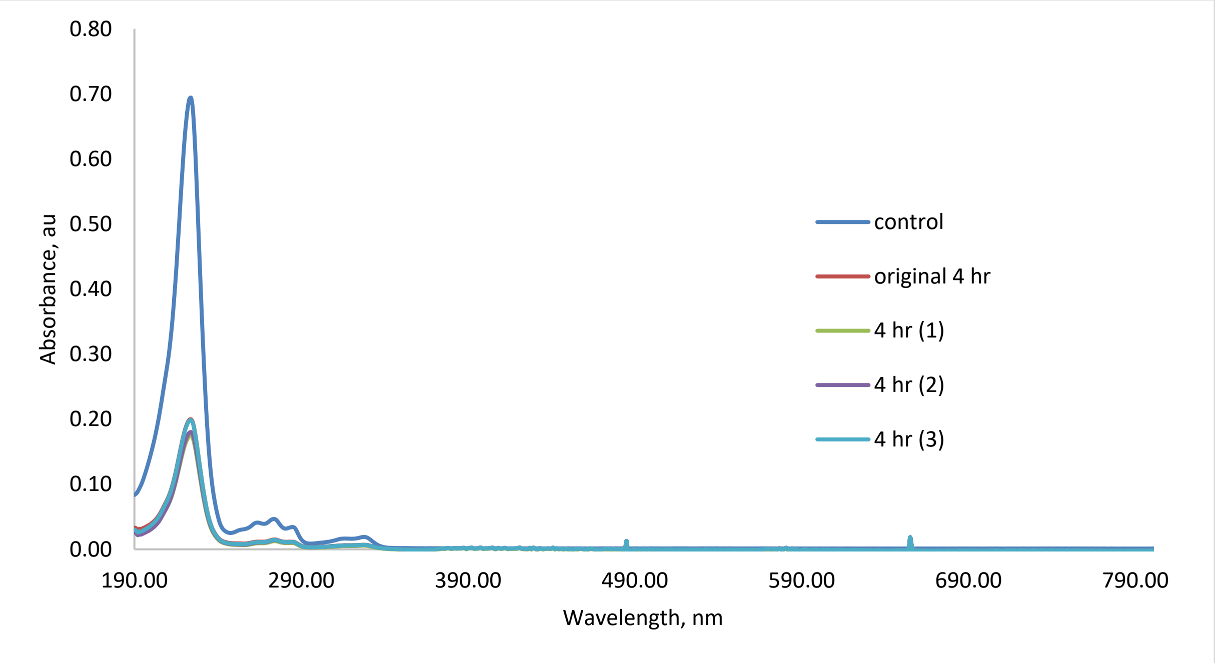


**Figure S34**. UV-Vis spectra for $\mathbf{P(4VP_3\text{-}co\text{-}BMA_1)_t}$ showing three additional trials at 4 hr binding time. Standard deviation: 0.02.

**<u>Binding data for crosslinked polymers with PPL-HCl water solution (Figures S35-S45)</u>:**

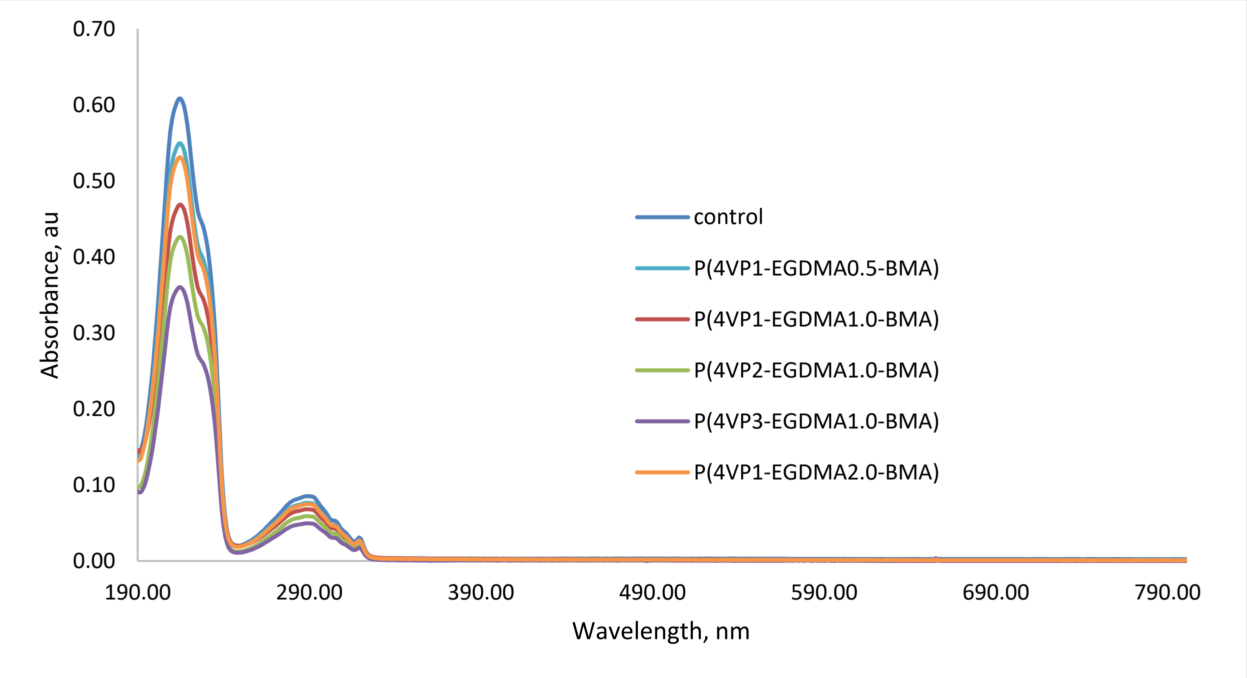


**Figure S35**. UV-Vis spectra of all crosslinked polymer beads following overnight (17 hr) binding experiment.

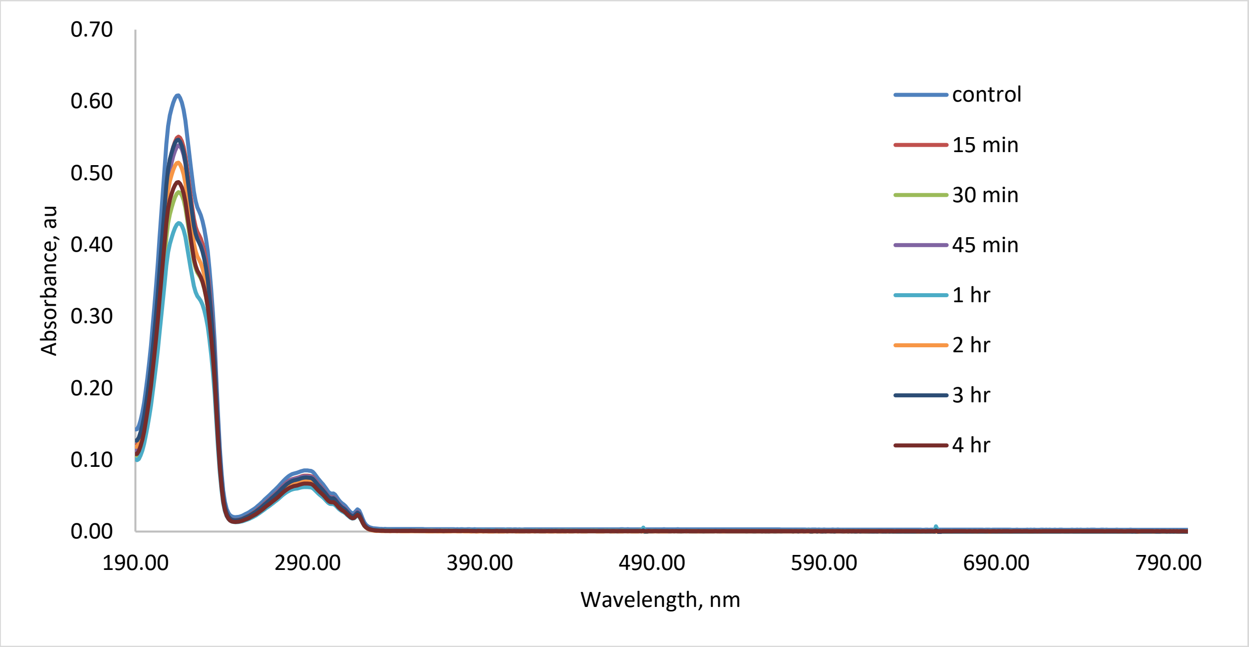


**Figure S36**. UV-Vis spectra of time-dependent binding experiment for **P(4VP$_1$-EGDMA$_{0.5}$-BMA)**.

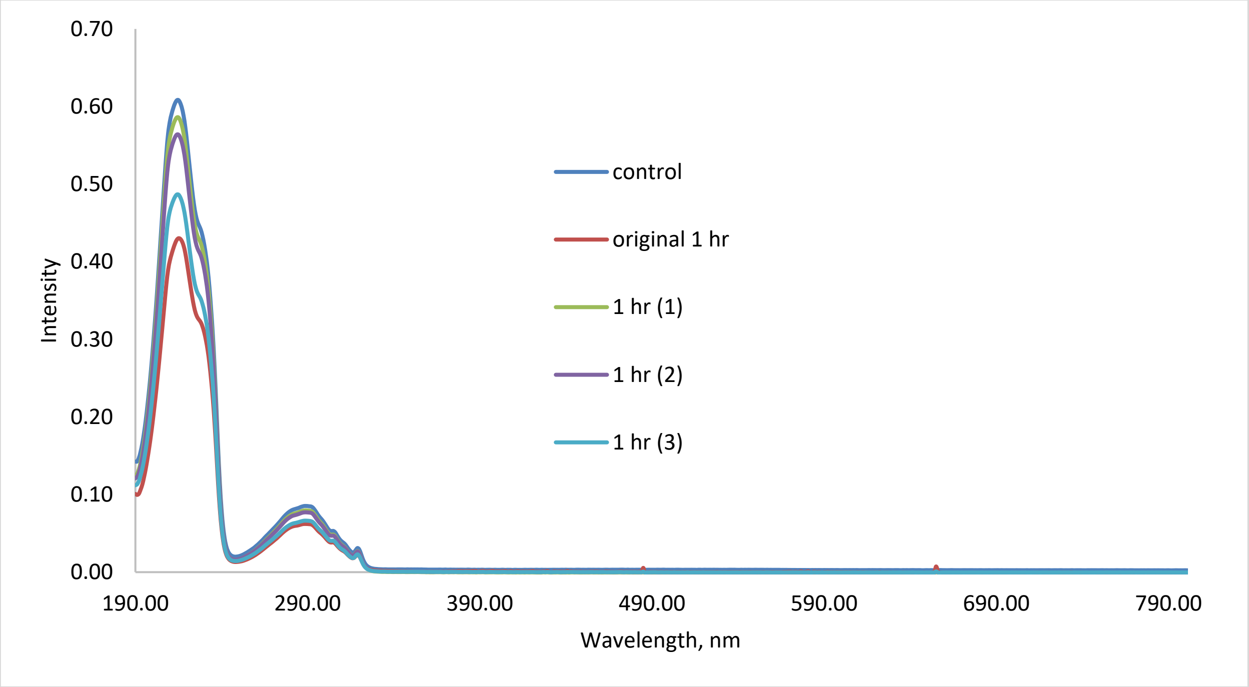


**Figure S37**. UV-Vis spectra for **P(4VP$_1$-EGDMA$_{0.5}$-BMA)** showing three additional trials at 1 hr binding time. Standard deviation: 0.12.

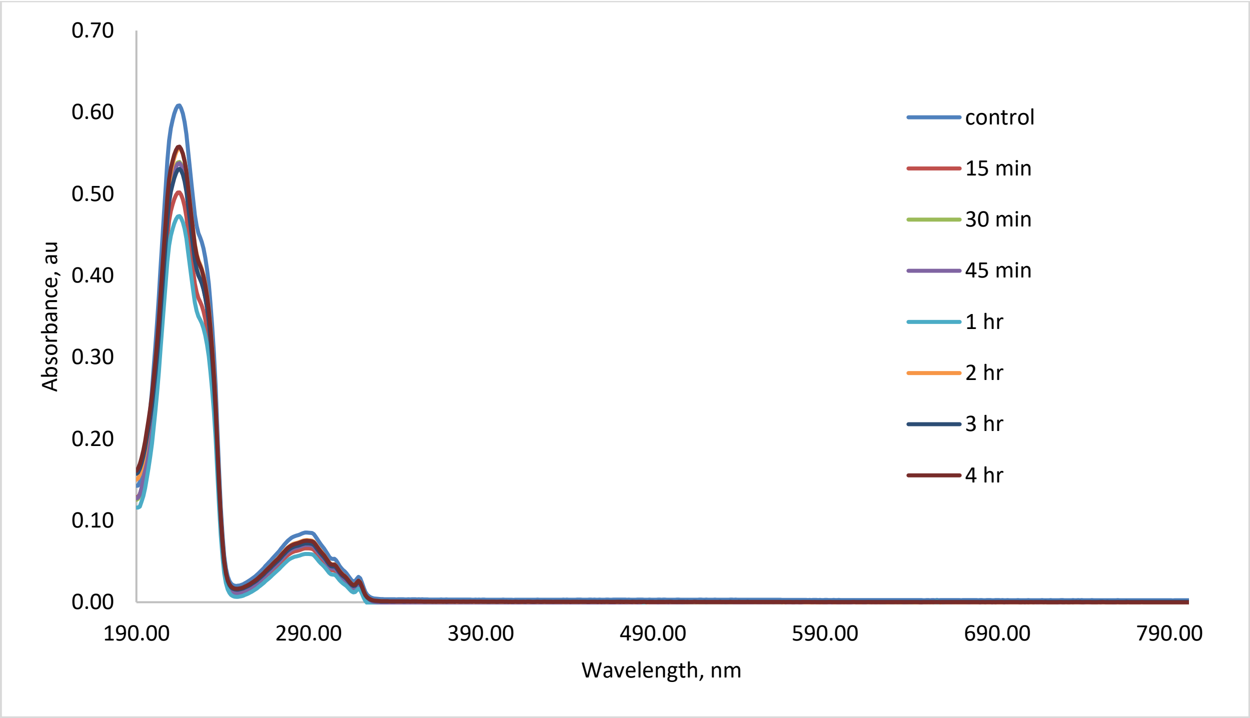


**Figure S38**. UV-Vis spectra of time-dependent binding experiment for **P(4VP$_1$-EGDMA$_{1.0}$-BMA)**.

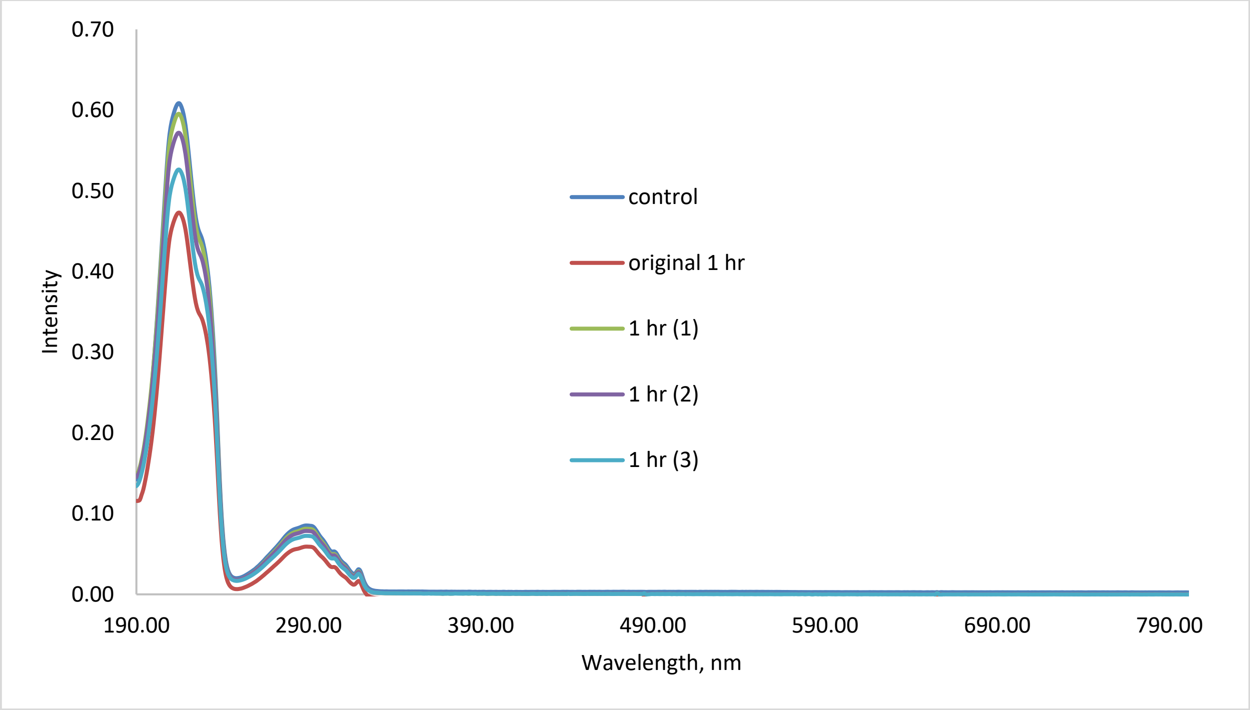


**Figure S39**. UV-Vis spectra for **P(4VP$_1$-EGDMA$_{1.0}$-BMA)** showing three additional trials at 1 hr binding time. Standard deviation: 0.09.

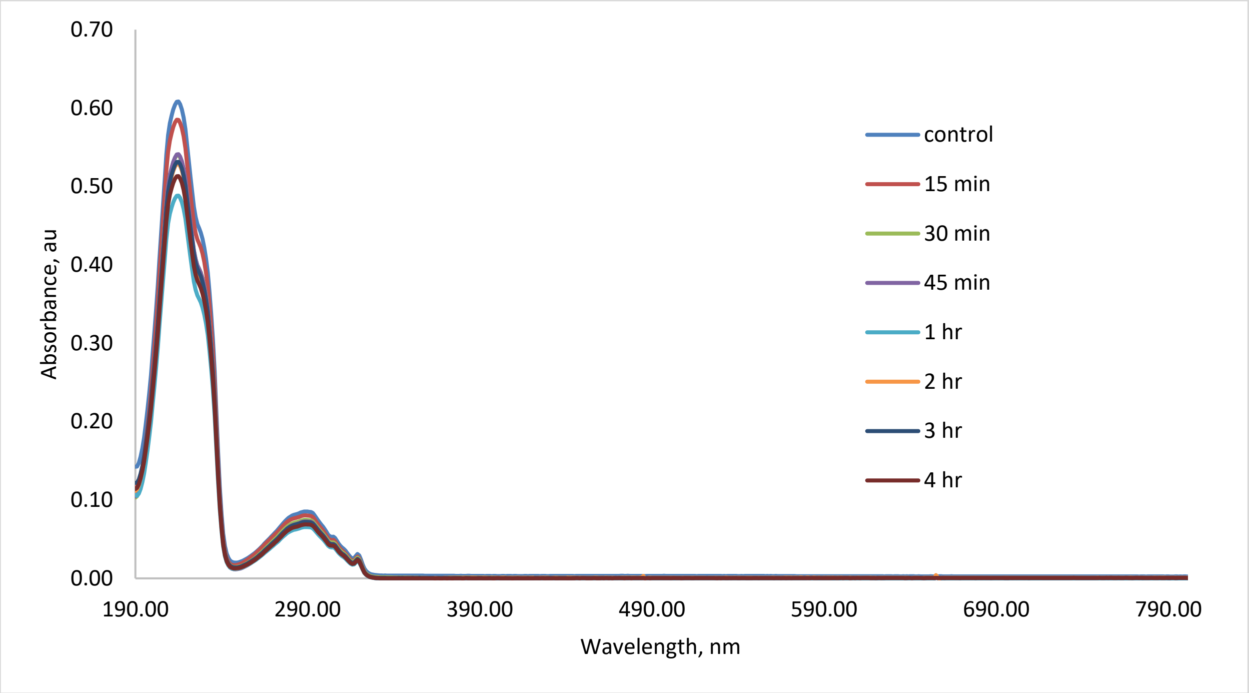


**Figure S40**. UV-Vis spectra of time-dependent binding experiment for **P(4VP$_2$-EGDMA$_{1.0}$-BMA**.

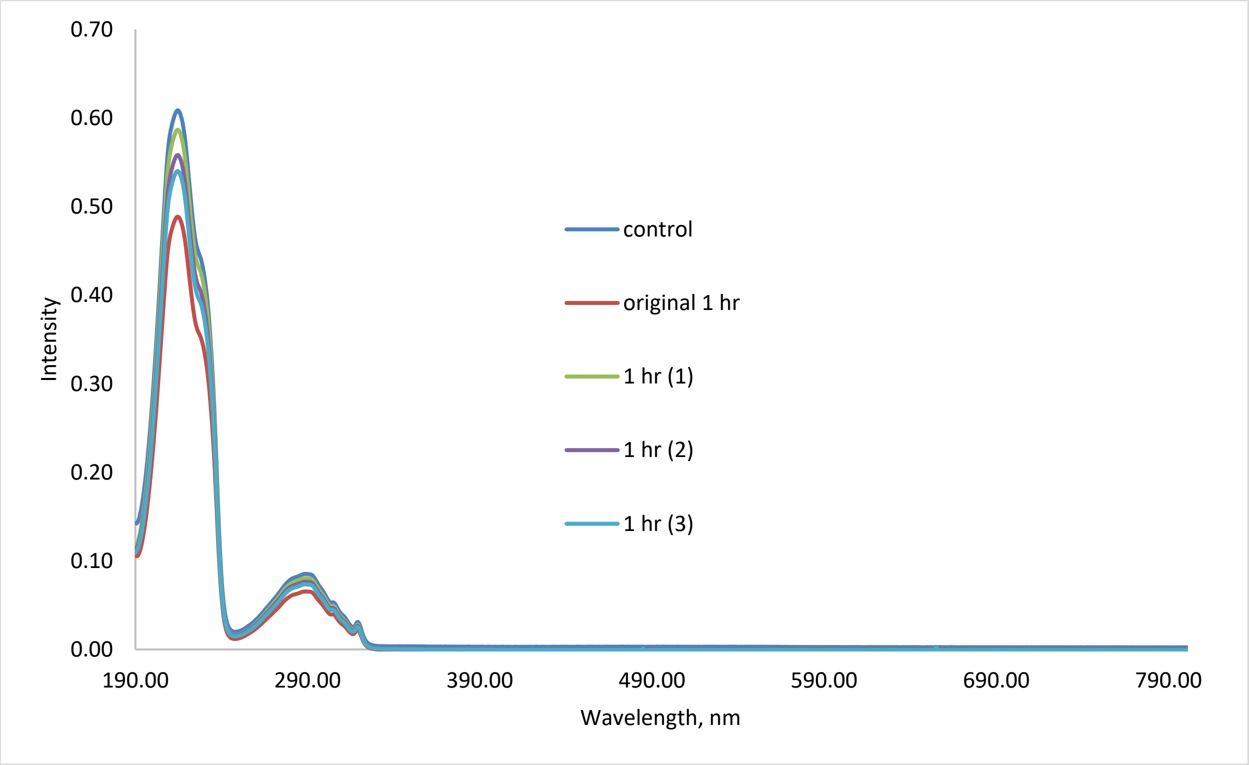


**Figure S41**. UV-Vis spectra for **P(4VP$_2$-EGDMA$_{1.0}$-BMA)** showing three additional trials at 1 hr binding time. Standard deviation: 0.07.

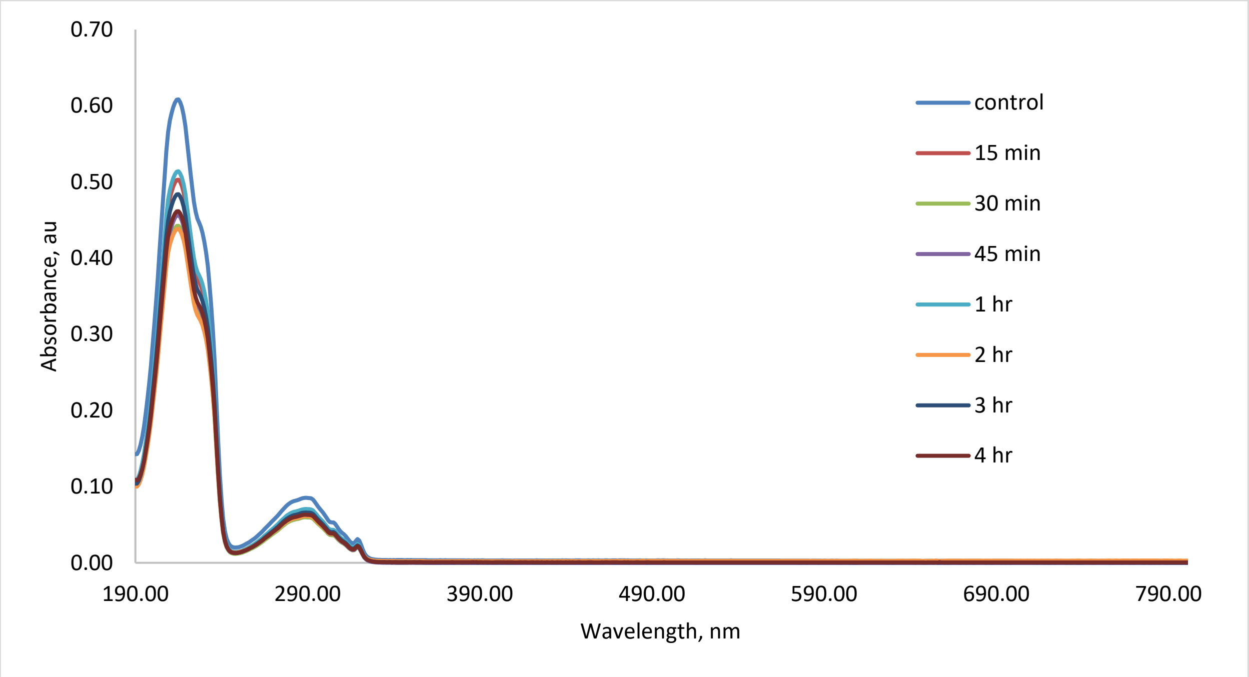


**Figure S42**. UV-Vis spectra of time-dependent binding experiment for **P(4VP$_3$-EGDMA$_{1.0}$-BMA)**.

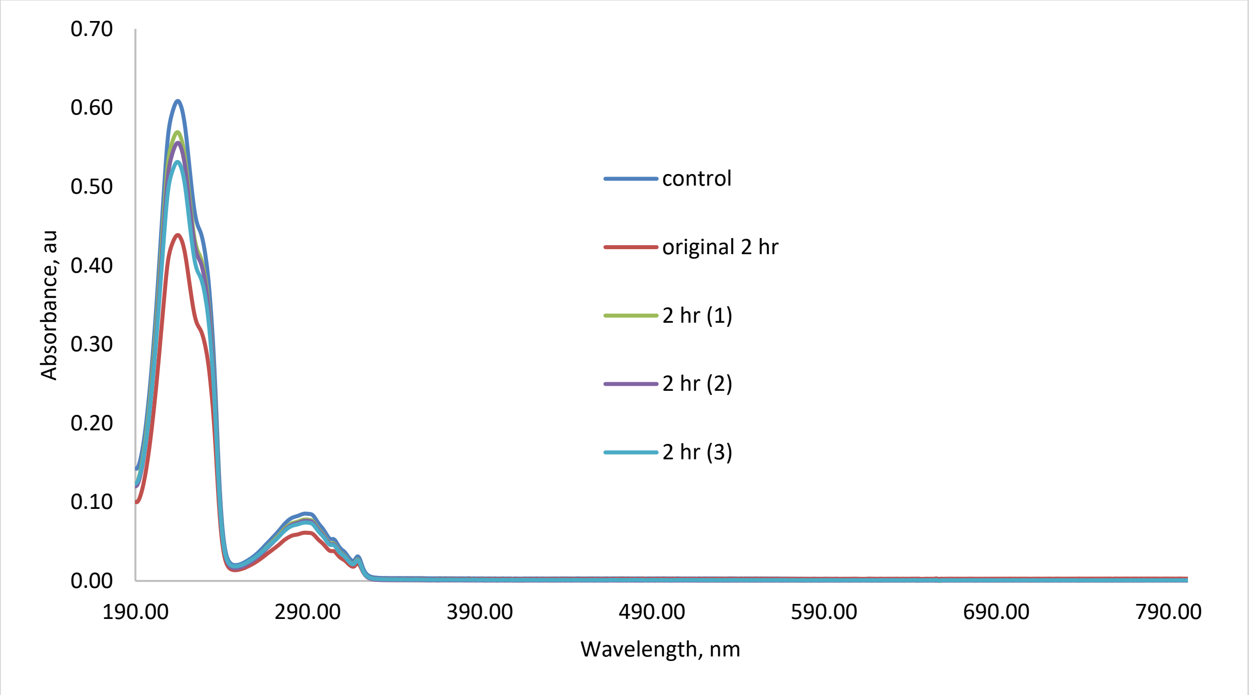


**Figure S43**. UV-Vis spectra for **P(4VP$_3$-EGDMA$_{1.0}$-BMA)** showing three additional trials at 2 hr binding time. Standard deviation: 0.10.

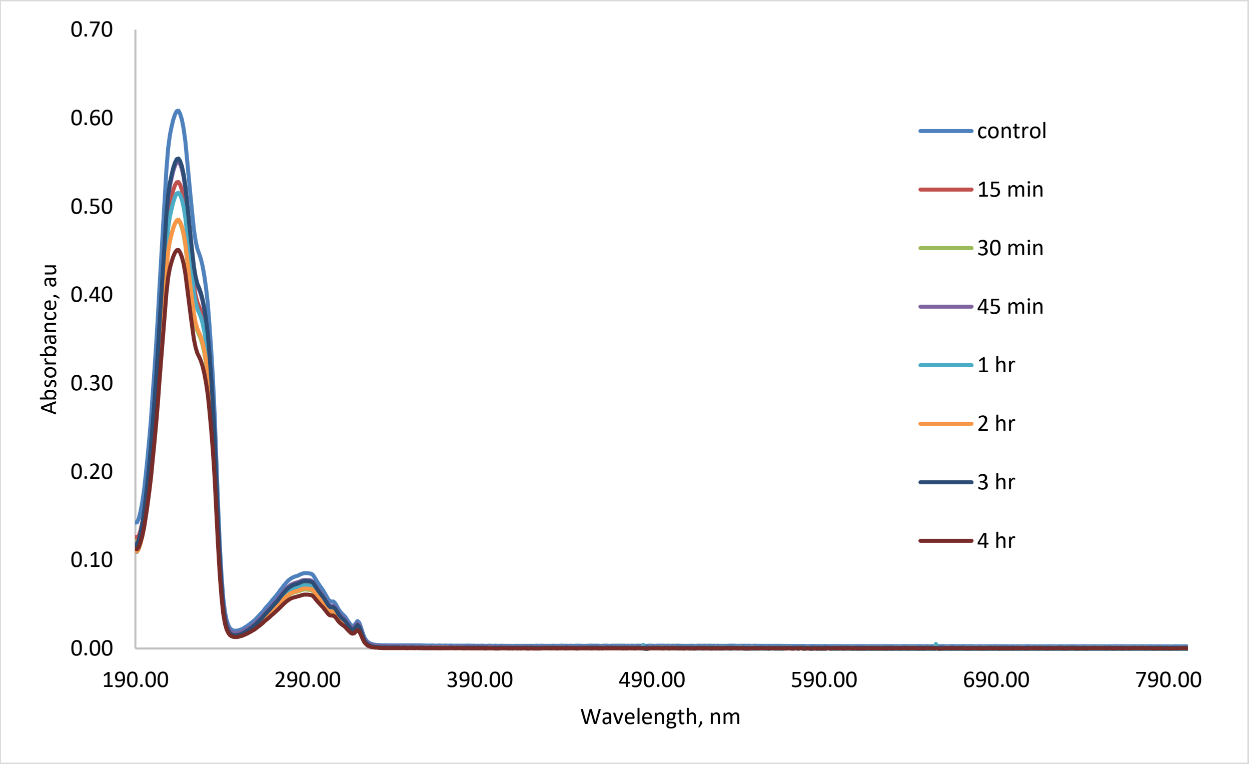


**Figure S44**. UV-Vis spectra of time-dependent binding experiment for **P(4VP$_1$-EGDMA$_{2.0}$-BMA)**.

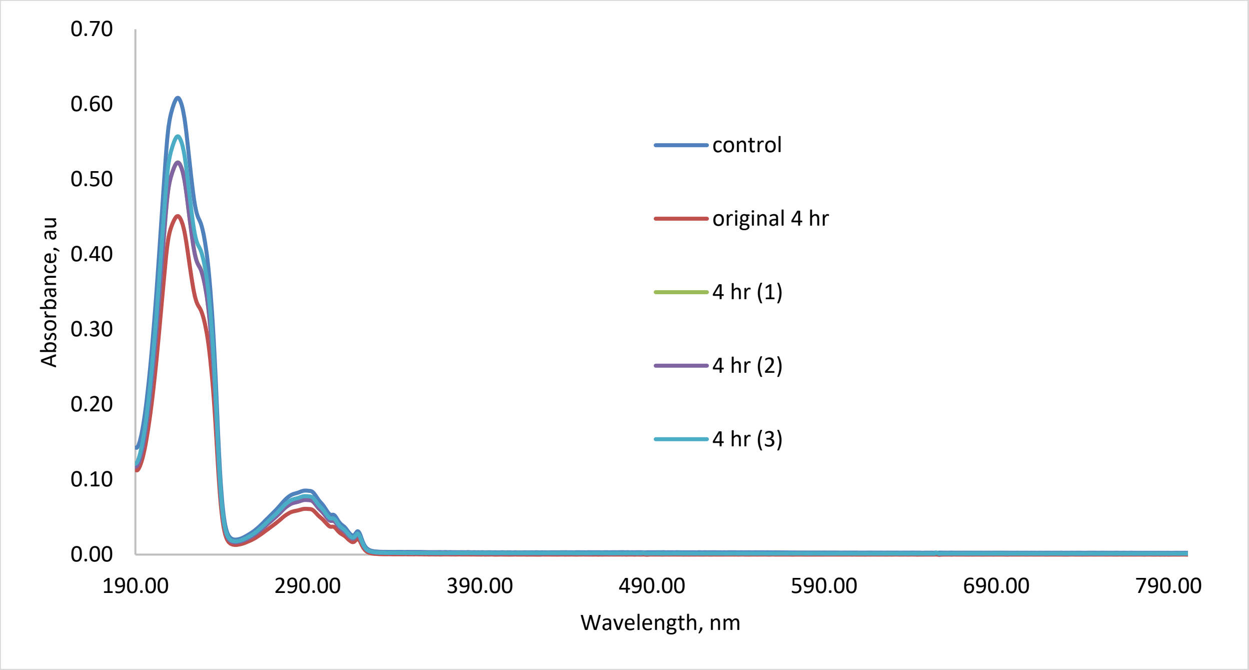


**Figure S45**. UV-Vis spectra for **P(4VP$_1$-EGDMA$_{2.0}$-BMA)** showing three additional trials at 4 hr binding time. Standard deviation: 0.07.

**Binding data for crosslinked polymers with 2-naphthol water solution (Figures S46-S56):**

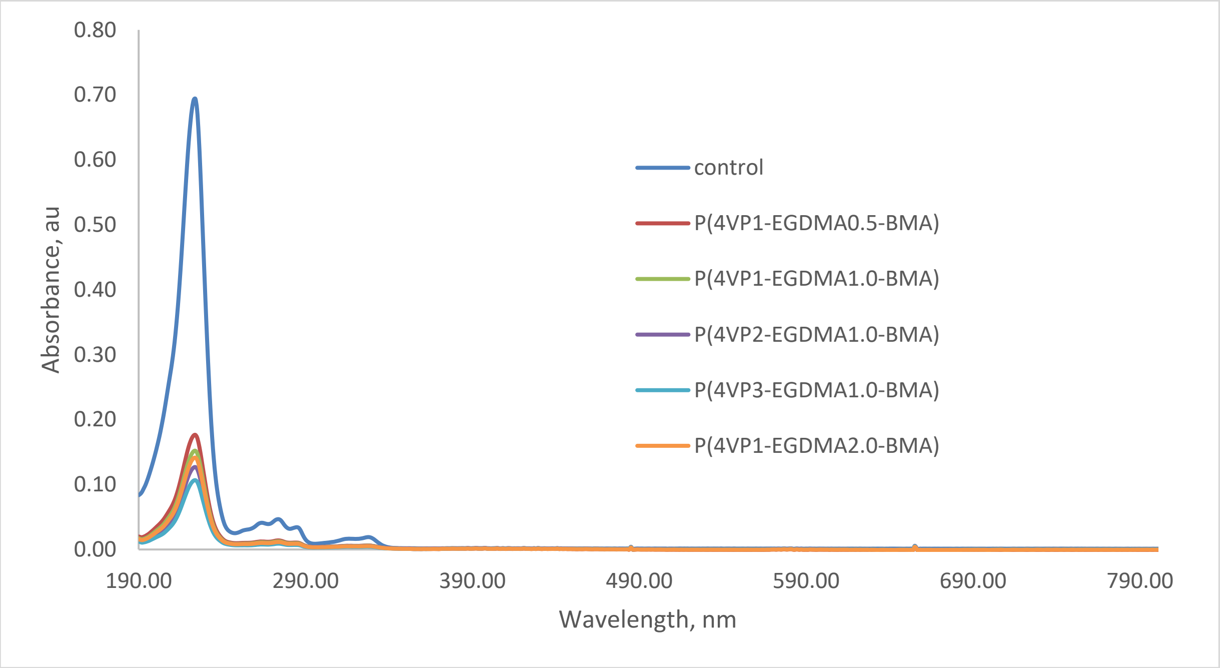


**Figure S46**. UV-Vis spectra of all crosslinked polymer beads following overnight (17 hr) binding experiment.

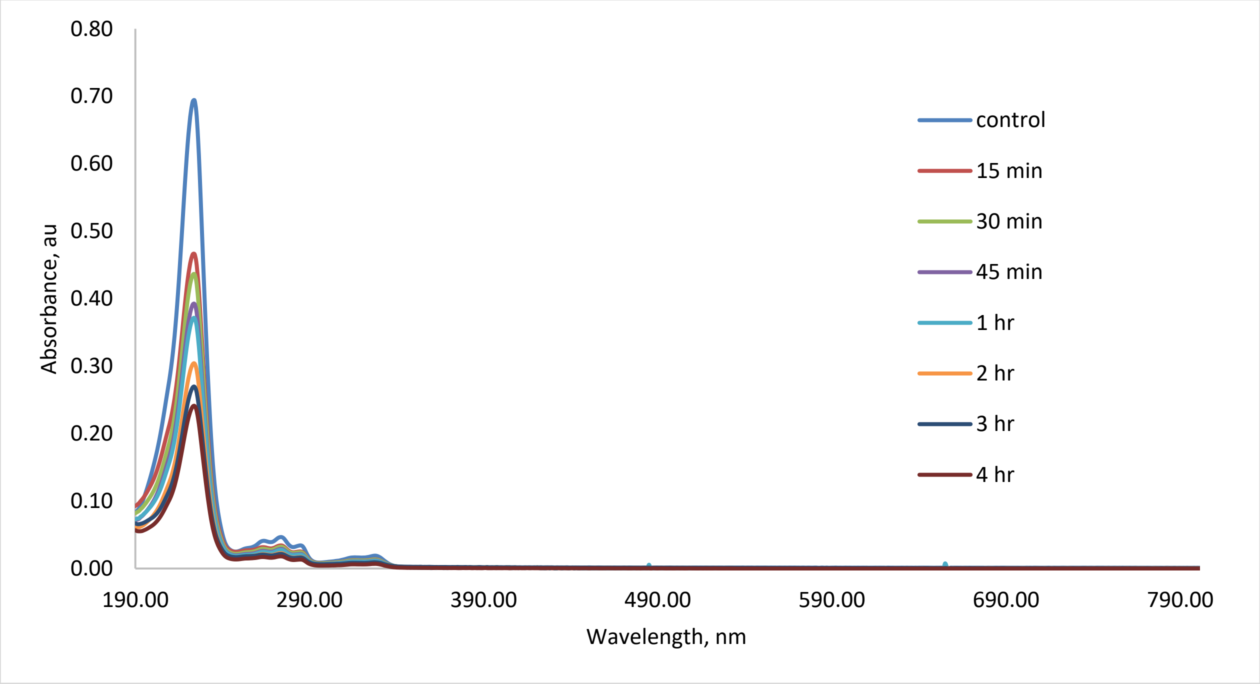


**Figure S47**. UV-Vis spectra of time-dependent binding experiment for **P(4VP$_1$-EGDMA$_{0.5}$-BMA)**.

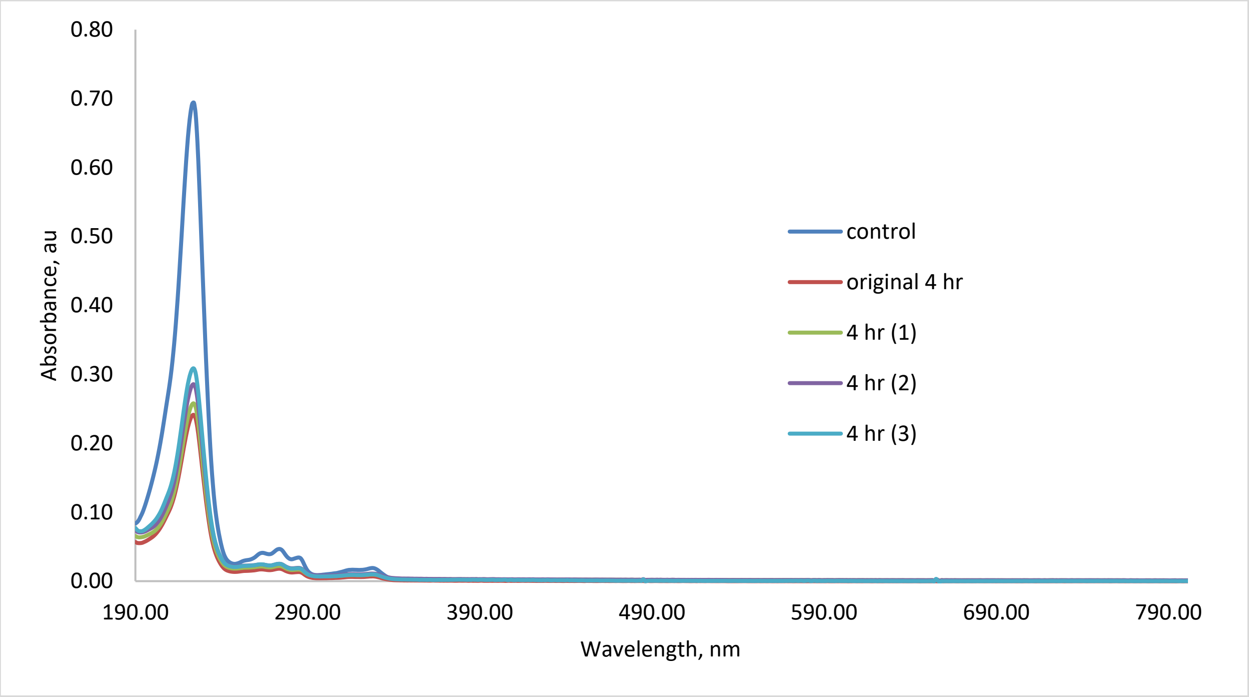


**Figure S48**. UV-Vis spectra for **P(4VP$_1$-EGDMA$_{0.5}$-BMA)** showing three additional trials at 4 hr binding time. Standard deviation: 0.04.

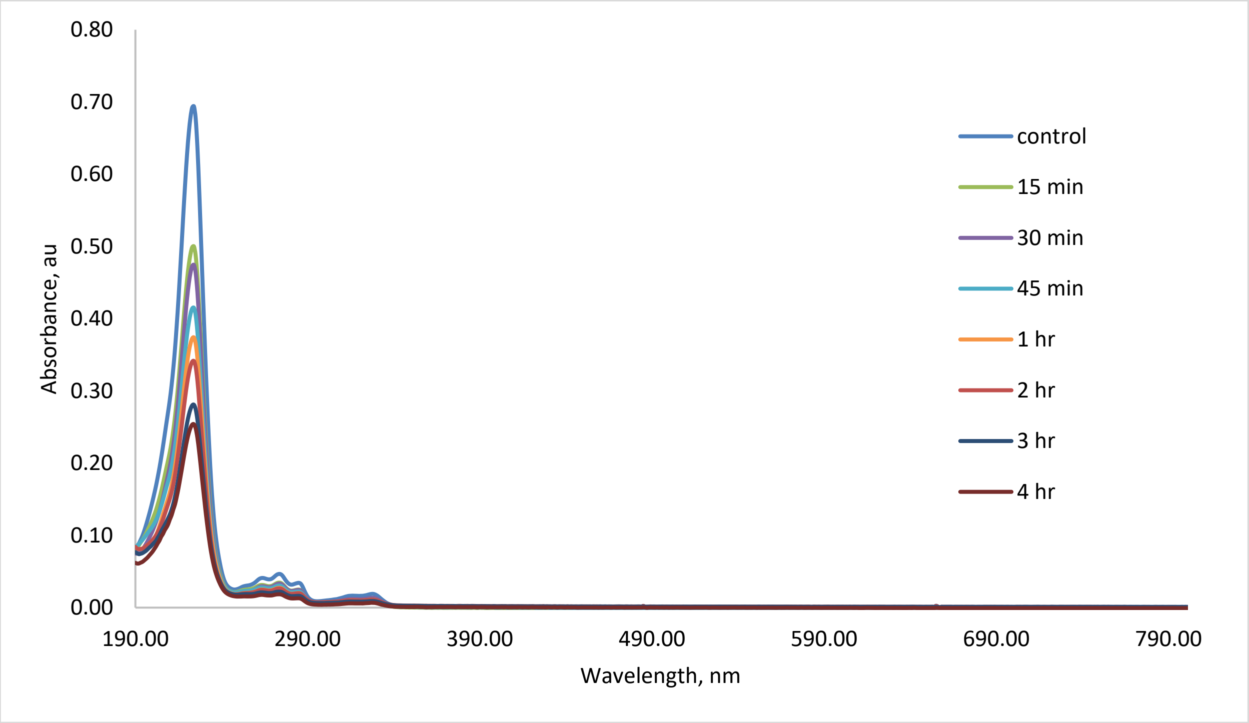


**Figure S49**. UV-Vis spectra of time-dependent binding experiment for **P(4VP$_1$-EGDMA$_{1.0}$-BMA)**.

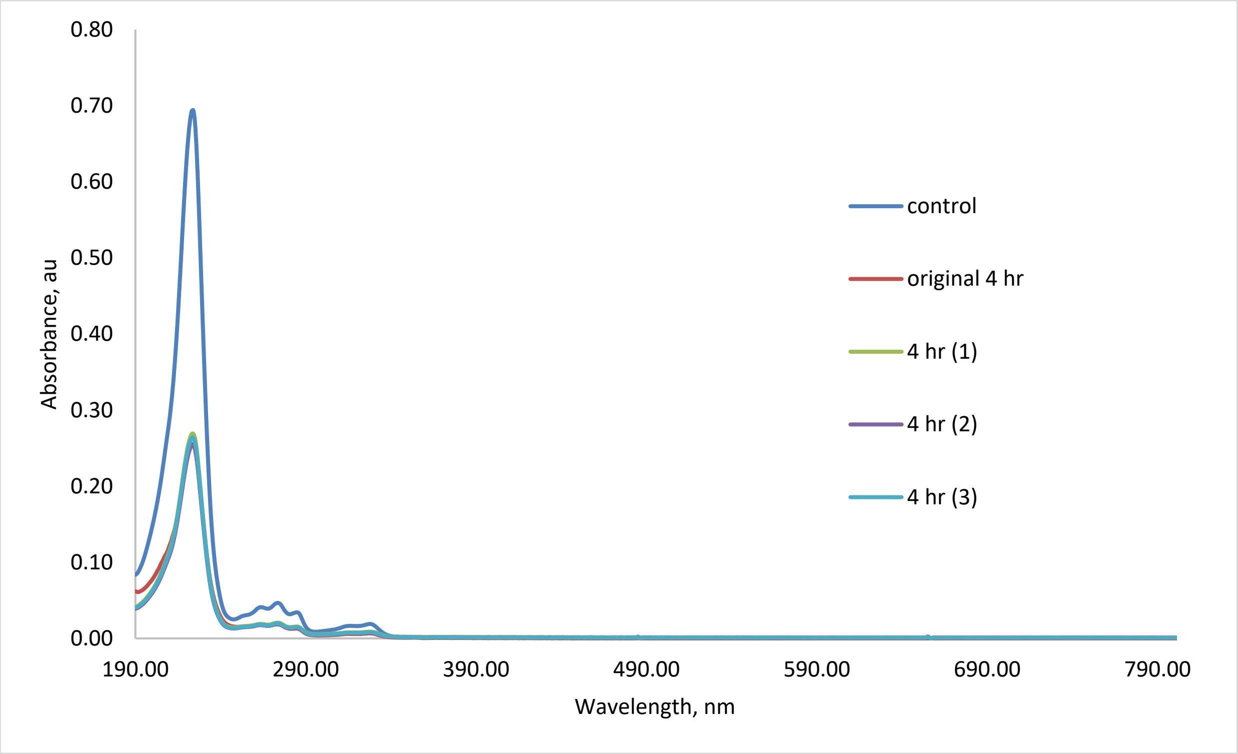


**Figure S50**. UV-Vis spectra for **P(4VP$_1$-EGDMA$_{1.0}$-BMA)** showing three additional trials at 4 hr binding time. Standard deviation: 0.01.

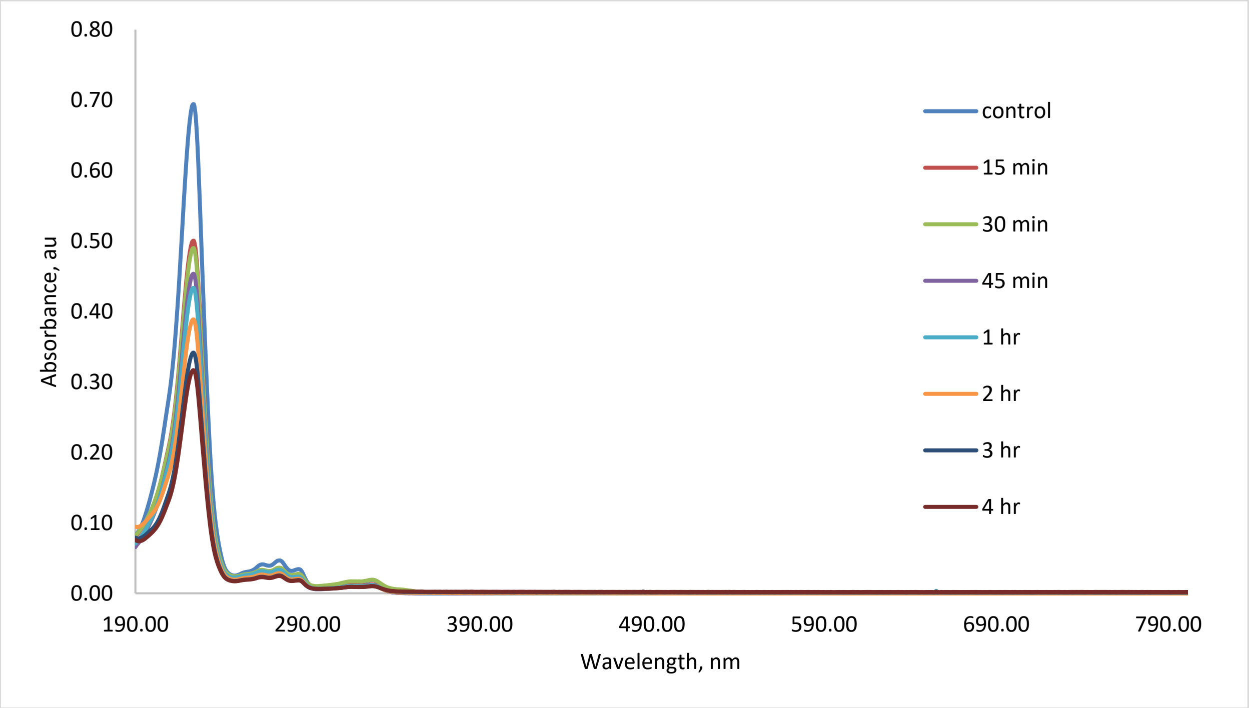


**Figure S51**. UV-Vis spectra of time-dependent binding experiment for **P(4VP$_2$-EGDMA$_{1.0}$-BMA)**.

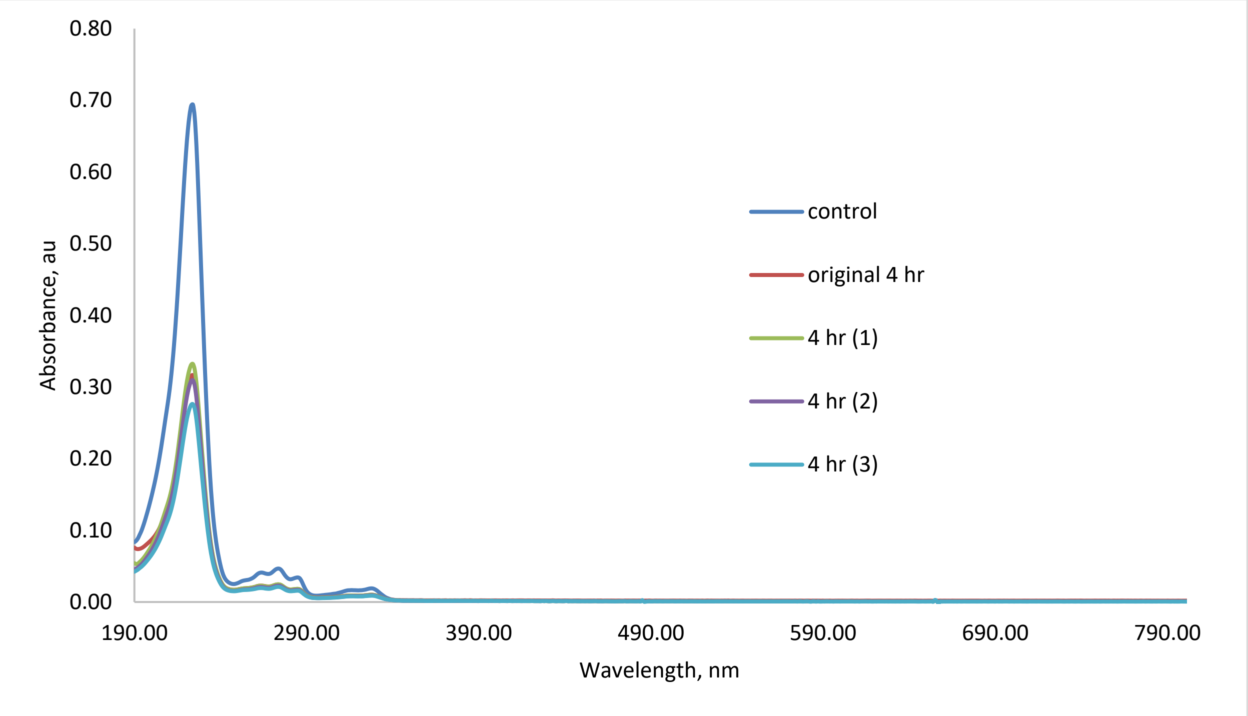


**Figure S52**. UV-Vis spectra for **P(4VP$_2$-EGDMA$_{1.0}$-BMA)** showing three additional trials at 4 hr binding time. Standard deviation: 0.03.

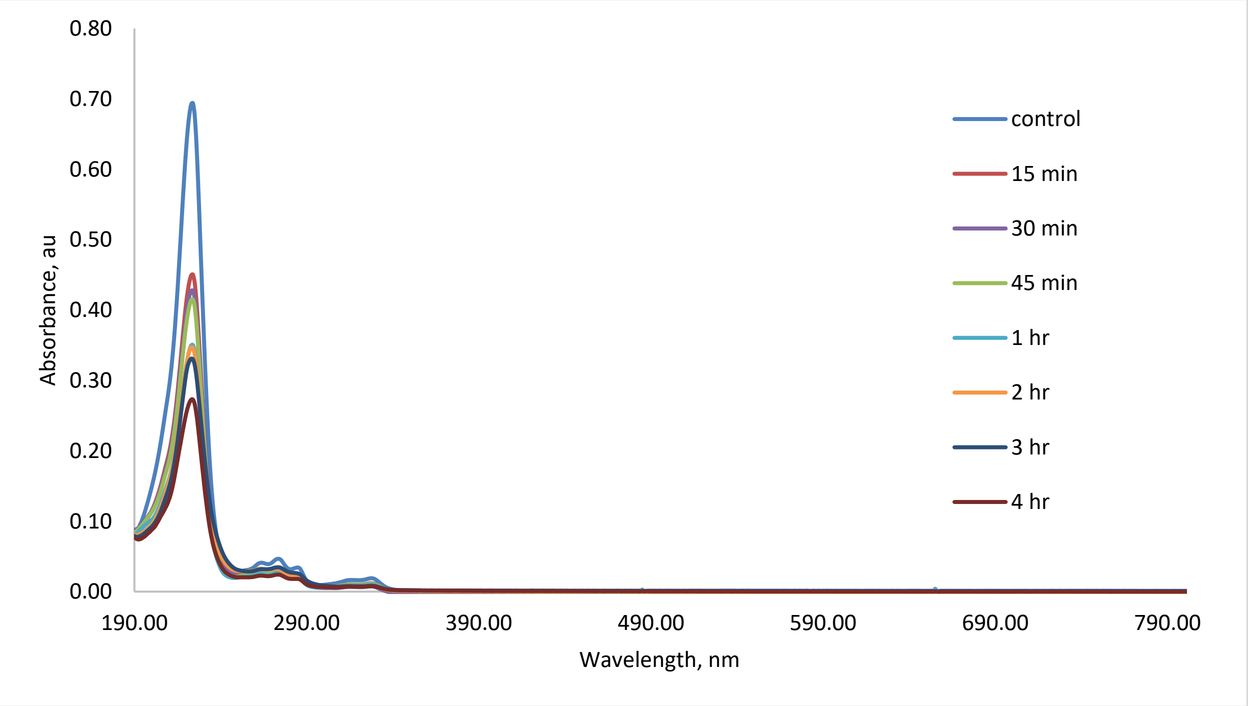


**Figure S53**. UV-Vis spectra of time-dependent binding experiment for **P(4VP$_3$-EGDMA$_{1.0}$-BMA)**.

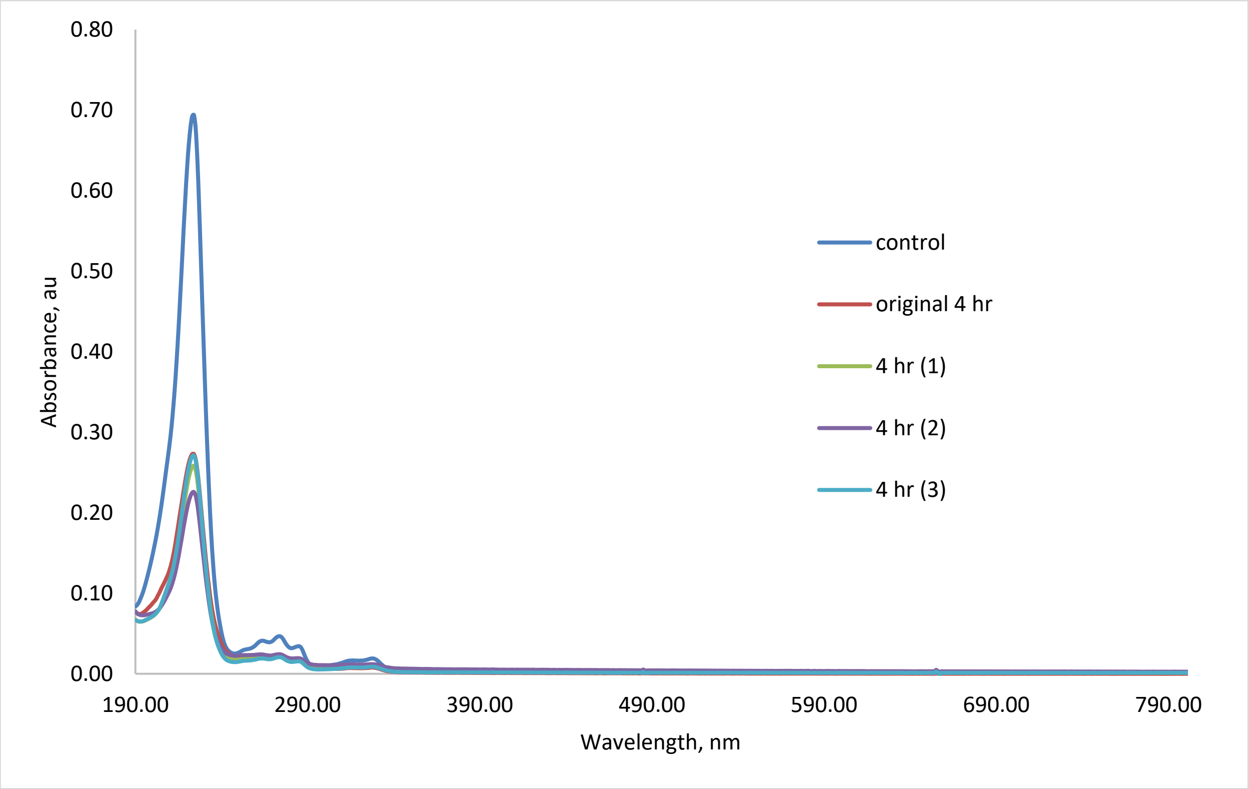


**Figure S54**. UV-Vis spectra for **P(4VP$_3$-EGDMA$_{1.0}$-BMA)** showing three additional trials at 4 hr binding time. Standard deviation: 0.03.

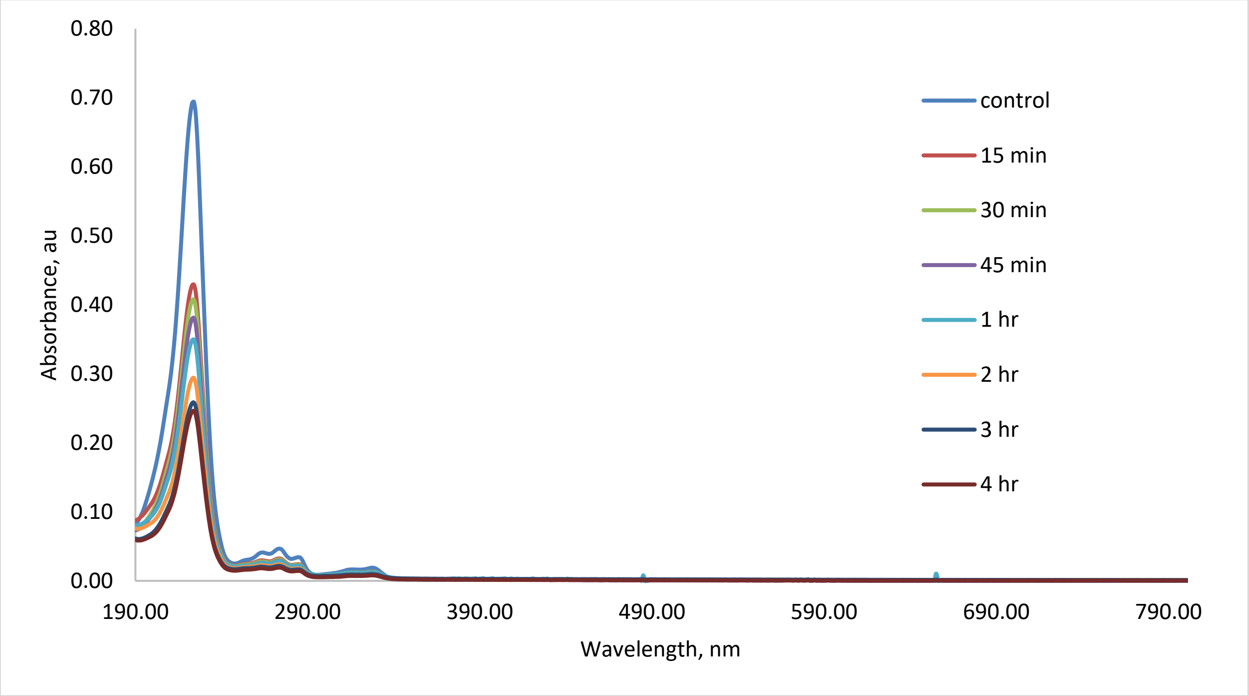


**Figure S55**. UV-Vis spectra of time-dependent binding experiment for **P(4VP$_1$-EGDMA$_{2.0}$-BMA)**.

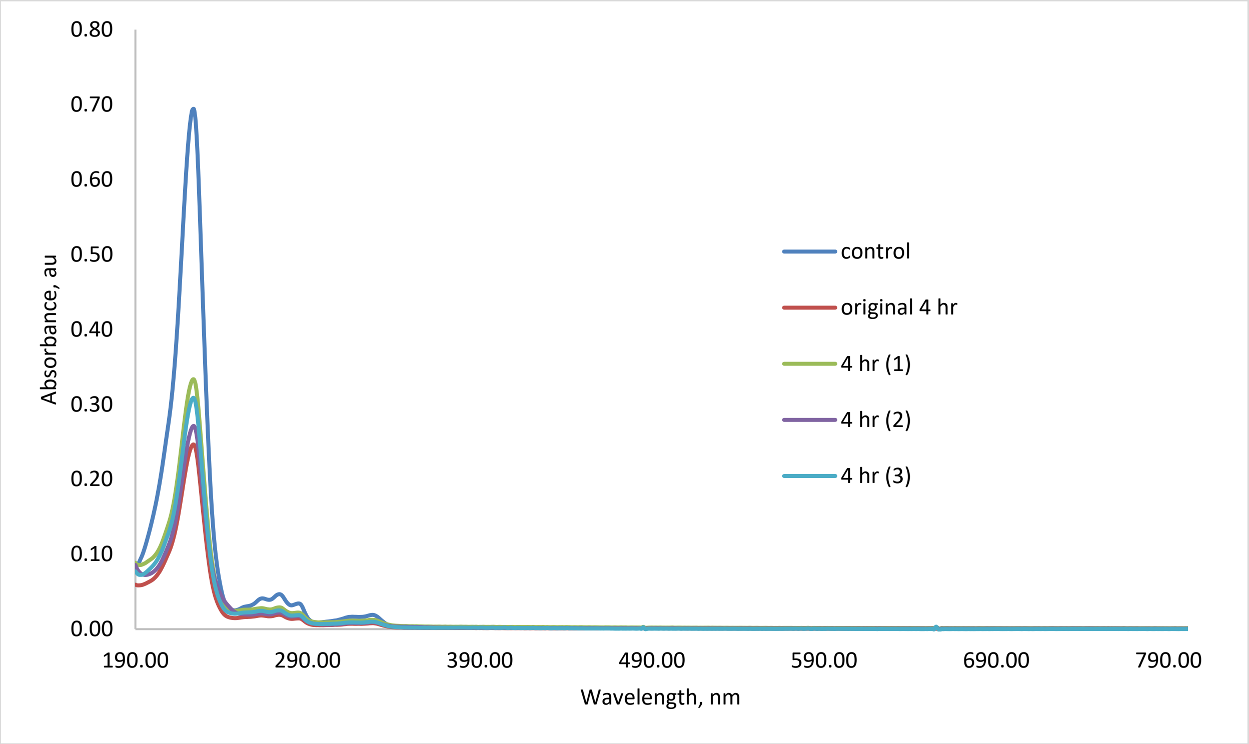


**Figure S56**. UV-Vis spectra for **P(4VP$_1$-EGDMA$_{2.0}$-BMA)** showing three additional trials at 4 hr binding time. Standard deviation: 0.06.

**Binding data for control crosslinked polymer with PPL-HCl and 2-naphthol water solutions**:

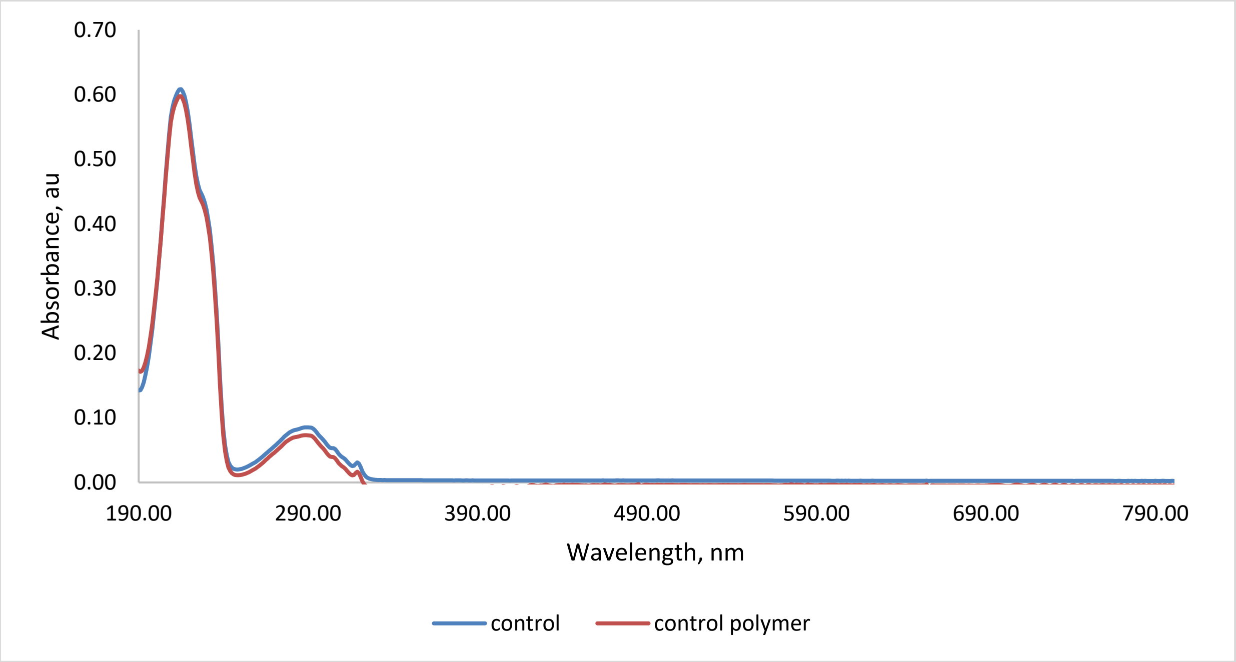


**Figure S57**. UV-Vis spectra for **P(STY$_3$-EGDMA$_{1.0}$-BMA)** binding **PPL-HCl** water solution at 2 hr binding time. The decrease in concentration is 2%.

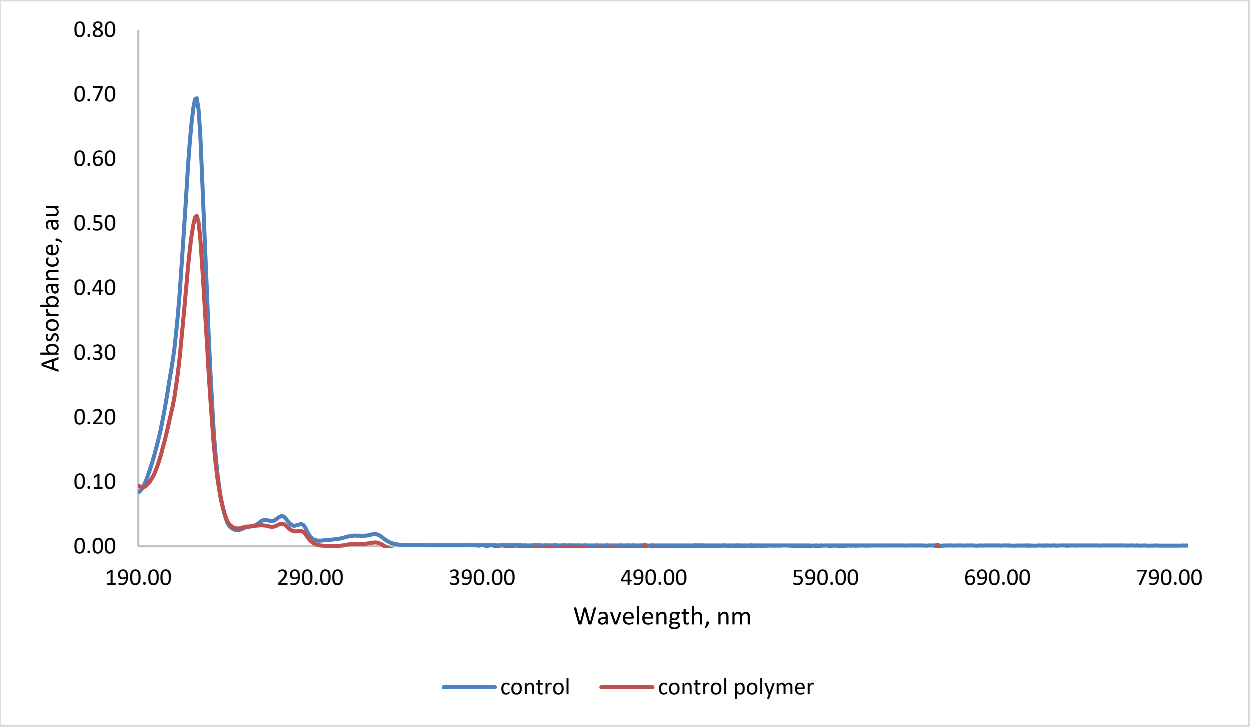


**Figure S58**. UV-Vis spectra for **P(STY$_3$-EGDMA$_{1.0}$-BMA)** binding **2NO** water solution at 4 hr binding time. The decrease in concentration is 26%.

**Summary of binding data**

**Table S2.** Optimal binding time and corresponding decreases in concentrations of contaminants following binding experiments for linear polymers.

| Linear polymer | Optimal binding time for **PPL-HCl** water solution | Concentration decrease (%) (±SD, %) | Optimal binding time for **2NO** water solution | Concentration decrease (%) (±SD, %) |
|---|---|---|---|---|
| **P(4VP$_1$-co-BMA$_1$)** | 4 h | 16 (±4) | 3 h | 62 (±4) |
| **P(4VP$_2$-co-BMA$_1$)** | 45 min | 21 (±5) | 4 h | 88 (±1) |
| **P(4VP$_3$-co-BMA$_1$)** | 4 h | 23 (±4) | 4 h | 88 (±2) |
| **P(4VP$_3$-co-BMA$_1$)$_t$** | 4 h | 16 (±6) | 4 h | 75 (±2) |

**Table S3.** Optimal time and corresponding decreases in concentrations of contaminants following binding experiments for crosslinked polymers.

| Crosslinked polymer | Optimal binding time for **PPL-HCl** water solution | Concentration decrease (%) (±SD, %) | Optimal binding time for **2NO** water solution | Concentration decrease (%) (±SD, %) |
|---|---|---|---|---|
| **P(4VP$_1$-EGDMA$_{0.5}$-BMA)** | 1 h | 30 (±12) | 4 h | 65 (±4) |
| **P(4VP$_1$-EGDMA$_{1.0}$-BMA)** | 1 h | 23 (±9) | 4 h | 64 (±1) |
| **P(4VP$_2$-EGDMA$_{1.0}$-BMA)** | 1 h | 20 (±7) | 4 h | 59 (±3) |
| **P(4VP$_3$-EGDMA$_{1.0}$-BMA)** | 2 h[a] | 28[a] (±10) | 4 h | 67 (±3) |
| **P(4VP$_1$-EGDMA$_{2.0}$-BMA)** | 4 h[b] | 26[b] (±7) | 4 h | 64 (±6) |

[a]A 1 h binding time for **P(4VP$_3$-EGDMA$_{1.0}$-BMA)** afforded a 16% decrease in concentration.
[b]A 1 h binding time for **P(4VP$_1$-EGDMA$_{2.0}$-BMA)** afforded a 15% decrease in concentration.

**Table S4.** Decreases in concentrations of contaminants following overnight binding experiments (17 h) for crosslinked polymers.

| Crosslinked polymer | Concentration decrease for **PPL-HCl** water solution (%) | Concentration decrease for **2NO** water solution (%) |
|---|---|---|
| **P(4VP$_1$-EGDMA$_{0.5}$-BMA)** | 10 | 74 |
| **P(4VP$_1$-EGDMA$_{1.0}$-BMA)** | 23 | 78 |
| **P(4VP$_2$-EGDMA$_{1.0}$-BMA)** | 30 | 81 |
| **P(4VP$_3$-EGDMA$_{1.0}$-BMA)** | 41 | 84 |
| **P(4VP$_1$-EGDMA$_{2.0}$-BMA)** | 13 | 80 |

**7. Reusability of the highest performing crosslinked polymer**

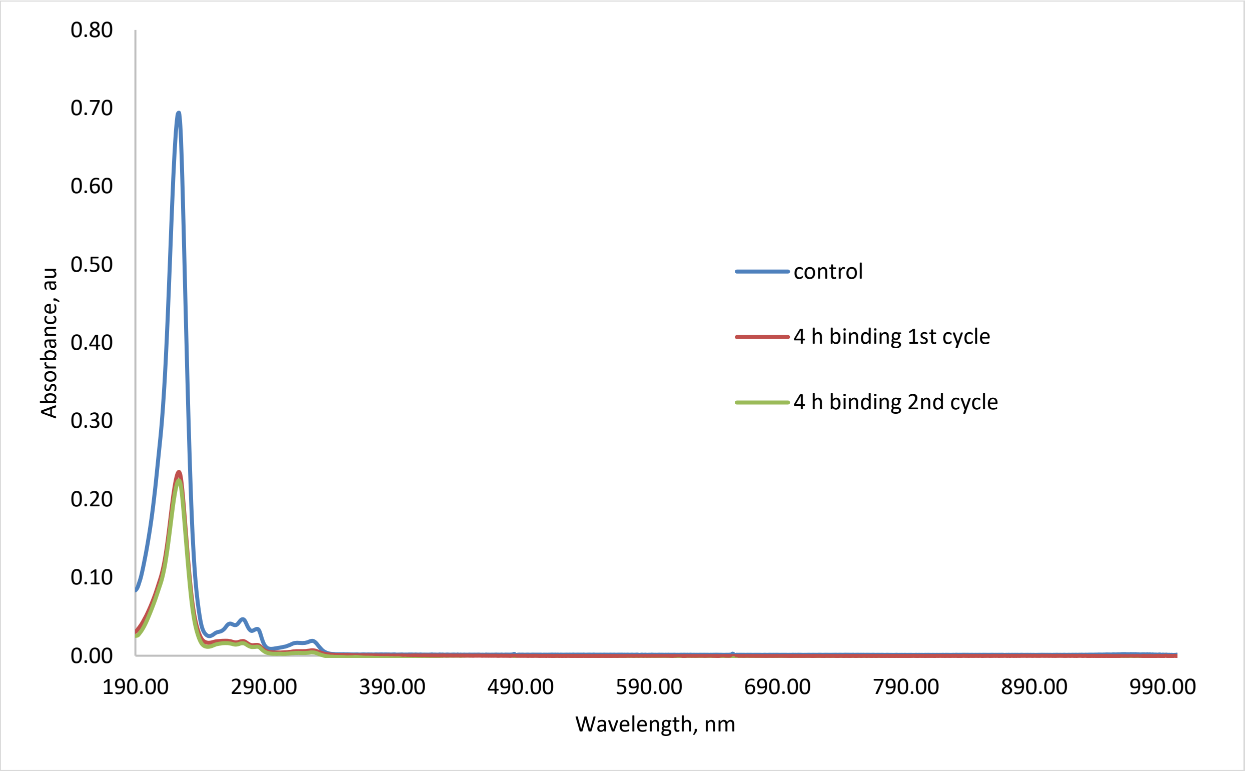


**Figure S59**. UV-Vis spectra showing reusability of **P(4VP$_3$-EGDMA$_{1.0}$-BMA)** with **2NO** water solution.

## 8. HPLC data

To support the UV-Vis studies, an HPLC experiment was conducted. The polymer **P(4VP$_1$-EGDMA$_{1.0}$-BMA)** was used in a binding experiment with the **2NO** water solution for a 4 hr binding time. A Thermo Scientific Utimate 3000 HPLC system with PDA detector at 227 nm was used. An Acclaim 120 C18 3x150 mm column and 38% acetonitrile-water containing 0.1% formic acid mobile phase was used at a flow rate of 0.4 mL/min. Chromeleon 7.2 was used for the instrument control, data acquisition, and processing.

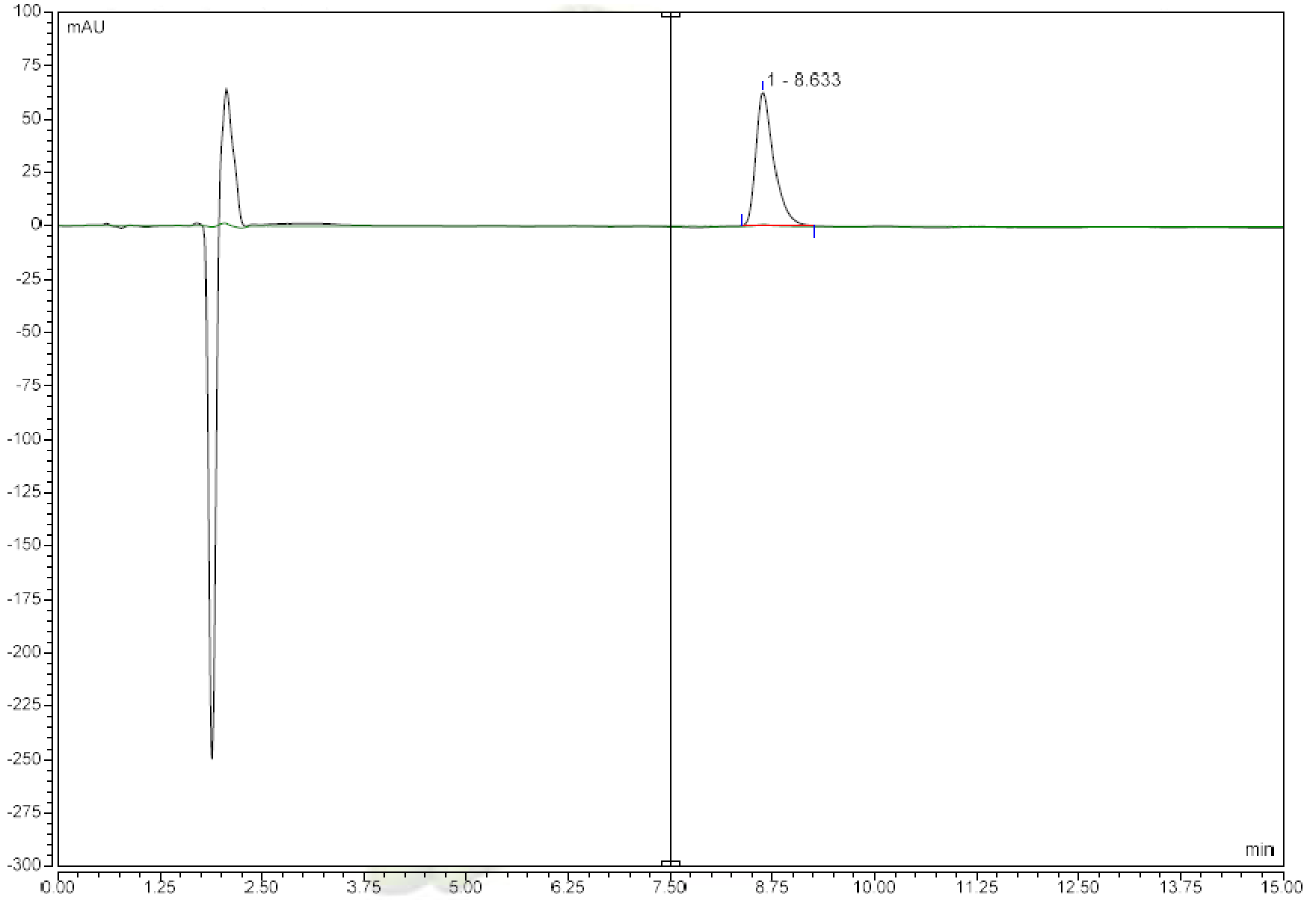


**Figure S60**. HPLC chromatogram of the stock **2NO** water solution.

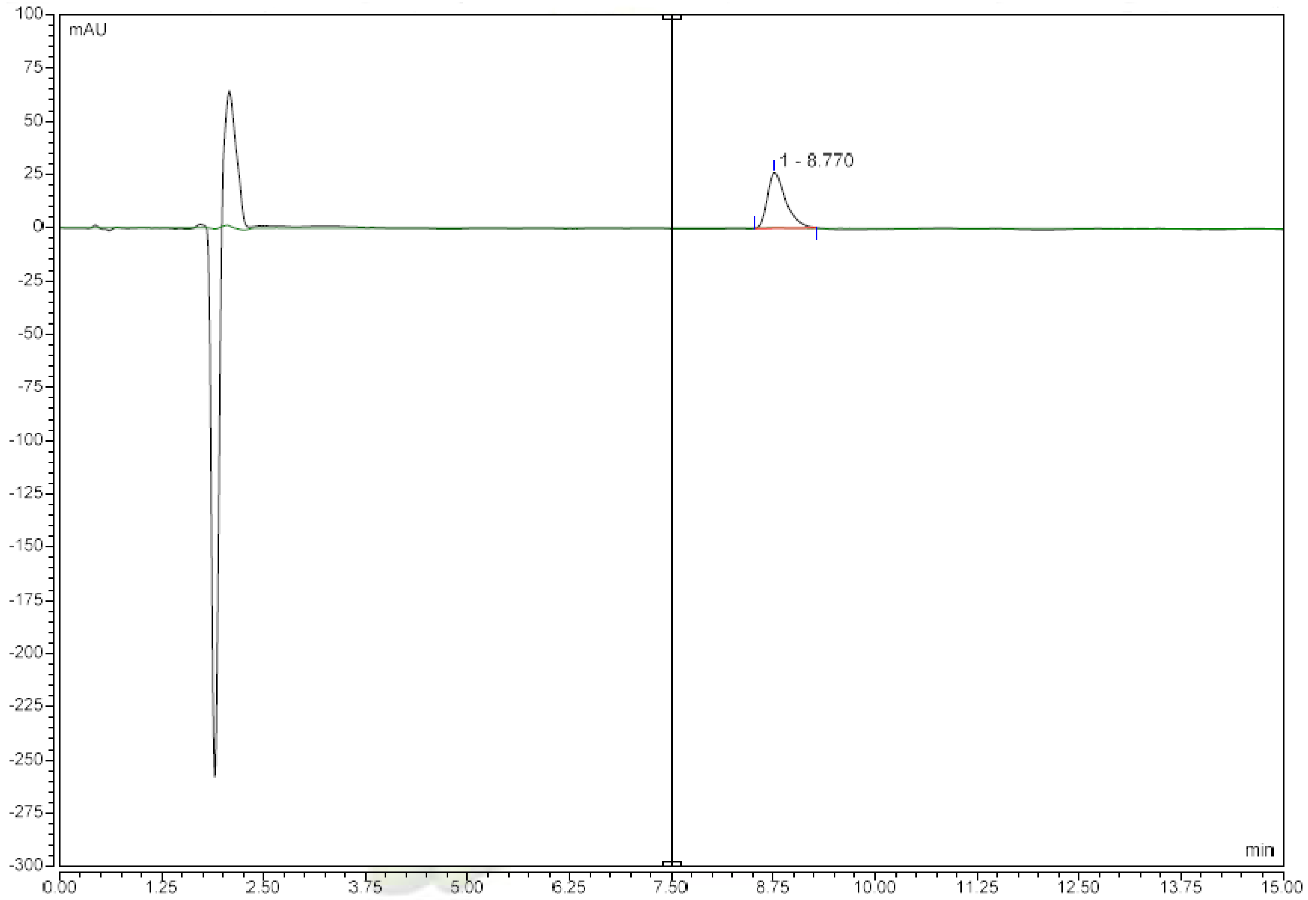


**Figure S61**. HPLC chromatogram of **2NO** water solution after 4 hr binding experiment with **P(4VP$_1$-EGDMA$_{1.0}$-BMA)**.

**Table S5**. HPLC and UV-Vis data for **P(4VP$_1$-EGDMA$_{1.0}$-BMA)** binding **2NO** water solution at 4 hr binding time.

| Sample | HPLC | | | UV-Vis | |
|---|---|---|---|---|---|
| | Retention time (min) | Height (mAU) | Area (mAU*min) | Wavelength (nm) | Absorbance (au) |
| Stock **2NO** solution | 8.633 | 62.5 | 15.9102 | 223 | 0.69 |
| **2NO** solution after binding for 4 hr | 8.770 | 26.02 | 6.7074 | 223 | 0.25 |
| Removal (%) | | 58 | 58 | | 64 |

## 9. Binding experiment results for other micropollutants

Binding experiments for other micropollutants including bisphenol A (phenol), bisphenol S (phenol), atenolol (structurally similar to propranolol but also contains an amide group), rhodamine B (carboxylic acid group), and gabapentin (carboxylic acid and amine groups) were conducted following by the same procedure as those for **PPL-HCl** and **2NO**. A solution of each contaminant in water was prepared and the concentrations are as follows: bisphenol A ($1x10^{-4}$ M), bisphenol S ($1x10^{-4}$ M), atenolol ($4x10^{-4}$ M), rhodamine B ($4x10^{-4}$ M), and gabapentin ($4x10^{-4}$ M). Binding experiments using the highest performing crosslinked pyridine functionalized polymer [**P(4VP$_3$-EGDMA$_{1.0}$-BMA)**] resulted in a decrease in concentration of these five contaminants by less than 5%. Binding experiments using the highest performing linear polymer [**P(4VP$_3$-co-BMA$_1$)**] resulted in a decrease in concentration of bisphenol A, bisphenol S, rhodamine B, and gabapentin by less than 5%.

**10. References**

(1) Goswami, S.; Dutta, A. Conductivity study of solid polyelectrolytes based on hydroiodide salt of poly(4-vinyl pyridine-co-butylmethacrylate), poly(4-vinyl pyridine-co-butylacrylate). *Bull. Mater. Sci.* **2013**, *36*, 635-640.

(2) Sekerak, N. M.; Hutchins K. M.; Luo, B.; Kang, J. G.; Braun, P. V.; Chen, Q.; Moore, J. S. Size control of cross-linked carboxy-functionalized polystyrene particles: Four orders of magnitude of dimensional versatility. *Eur. Polym. J.* **2018**, *101*, 202-210.

(3) CrysAlis$^{Pro}$ (2018) Oxford Diffraction Ltd.

(4) SCALE3 ABSPACK (2005) Oxford Diffraction Ltd.

(5) Sheldrick, G. M. Crystal structure refinement with SHELXL. *Acta Crystallogr*. **2015**, *C71*, 3-8.
